# Straightforward, scalable, solution-phase synthesis of peptide bonds in flow

**DOI:** 10.1007/s41981-025-00347-2

**Published:** 2025-03-12

**Authors:** Zoe E. Wilson, Enol Lopez, Nils J. Flodén, Charis Watkins, Giulia Bianchini, Steven V. Ley

**Affiliations:** 1 Yusuf Hamied Department of Chemistry, University of Cambridge, Lensfield Road, Cambridge, CB2 1EW UK; 2 School of Chemical Sciences, University of Auckland, Auckland, New Zealand; 3 Department of Organic Chemistry, School of Engineering, University of Vallodolid, Valladolid, Spain; 4 Department of Biomolecular Systems, Max-Planck Institute of Colloids and Interfaces, 14476 Potsdam, Germany

**Keywords:** Amide bond, Peptide, Solution phase, Mixed anhydride

## Abstract

We report the development of simple solution-phase flow conditions for the scalable synthesis of peptides using in-situ activation as mixed anhydrides, contributing to the synthetic toolbox of available solution-phase flow reactions. This approach has been used for the preparation of a series of diverse dipeptides in plug flow, and then continuous flow conditions (< 10 mmol) were developed and applied to the gram scale synthesis of the hexapeptide linear precursor for the bioactive cyclic peptide segetalin A as further proof of the utility of this approach.

**Graphical Abstract:**

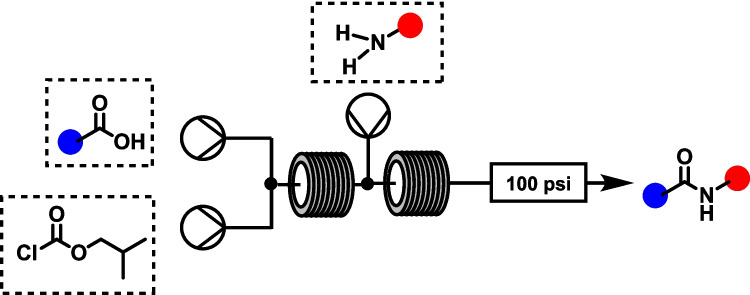

**Supplementary Information:**

The online version contains supplementary material available at 10.1007/s41981-025-00347-2.

## Introduction

The chemical synthesis of peptides has garnered considerable attention due to the wide-reaching potential shown by these often bioactive molecules, which has led to their increasing therapeutic use [1]. To achieve the construction of these short chain fragments, the biomimetic approach of amide bond formation between amino acid monomers has been by far the most convenient and studied strategy [2]. In fact, analysis carried out by Brown and Boström in 2016 shows that amide bond formation was the most frequently carried out reaction in medicinal chemistry programs [3]. Continuous flow approaches offer many benefits when compared with batch chemistry, including efficient transfer of heat and ease of automation [4], which has led to increasing focus on the use of flow chemistry for the syntheses of peptides [5–7]. While fast flow approaches to SPPS have been shown to dramatically accelerate peptide synthesis, even enabling the synthesis of proteins [8–10], the development of solution phase approaches is highly desirable as it avoids the use of solid support resins. Early research into solution phase peptide bond formation in flow focused on coupling to form beta-peptides [11, 12], which lack the challenging epimerizable centre of conventional peptides.

**Fig. 1:**
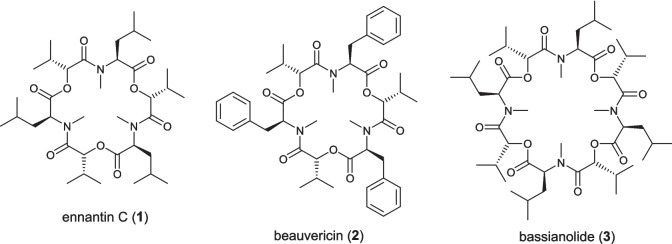
Depsipeptide natural products synthesised in our group using acid chloride mediated peptide couplings in flow [16]

In previous work, we have developed a multi-step synthesis of peptides in flow using solid supported reagents and scavengers [13] and used *N*-carboxybenzyl protected *N*-carboxyanhydrides (Cbz-NCAs) to achieve an automated solution phase flow synthesis of secondary amides [14]. We have additionally used Ghosez’s reagent both in batch [15] and flow [16] to enable to effective coupling of sterically hindered *N*-methyl amino acids via acid chloride intermediates. This approach was successfully applied to the atom efficient synthesis of a series of depsipeptides including natural products enniatin C (**1**), beauvericin (**2**) and bassianolide (**3**) (Fig. 1) [15, 16]. Other groups have additionally shown that flow approaches allow the safe synthesis of peptides via both hazardous reagents such as triphosgene (Fuse, 2014) [17, 18] and hazardous intermediates, such as acyl azides (Hone and Kappe, 2020) [19].

**Scheme 1:**
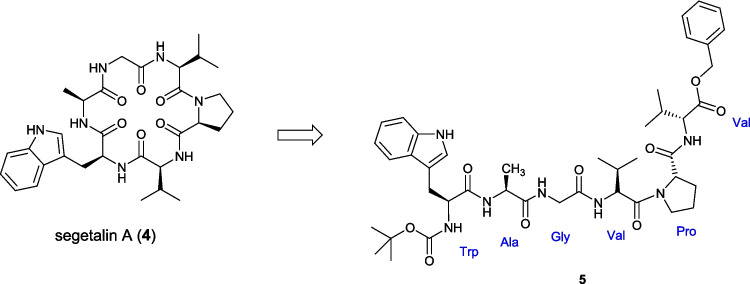
Segetalin A (**4**) and the targeted hexapeptide linear precursor **5**

While this approach proved successful for the synthesis of the tertiary amide bonds found in the cyclooligomeric depsipeptides, it is desirable to develop approaches for the synthesis of secondary amides, which represent the majority of naturally derived amide bonds, directly from amino acids without the use of solid supported reagents. Segetalin A (**4**) was isolated from the seeds of *Viccaria segetalis*, a flower of the carnation family, in 1994 [20] and, as well as several other members of the wider segetalin family, exhibits moderate estrogen-like and slow vasorelaxant properties [21, 22]. We intended to utilise the same Boc-/Benzyl ester protecting group strategy, which had proven successful from our previous depsipeptide synthesis. Herein we report work focused on achieving a scalable flow synthesis of hexapeptide **5**, the linear precursor for segetalin A (4, Scheme 1) as wider proof of concept for the flow enabled synthesis of secondary amides.

### Acid chloride mediated coupling

**Scheme 2:**
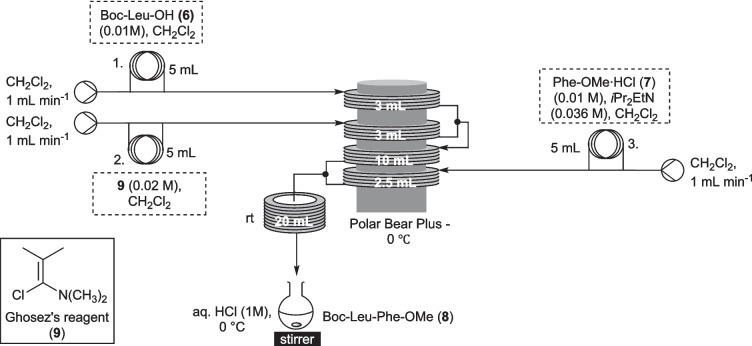
Dipeptide **8** synthesis using previously developed acid chloride mediated flow conditions [16]

During our previous flow integrated depsipeptide synthesis we utilised the coupling of *tert*-butyloxycarbonyl-leucine (Boc-Leu-OH, **6**) and phenylalanine methyl ester⋅HCl (Phe-OMe⋅HCl, **7**) as a test system to develop the key acid chloride mediated coupling step, which was seen to afford the coupled product (**8**) in modest yields in both plug (0.05 mmol, 46%) and continuous (0.6 mmol, 47%) flow (Scheme 2) [16]. However, upon further investigation of scope, it quickly became evident that while some simple combinations of Boc-protected amino acids could be coupled in respectable yields, others (including the protected tryptophan which would be necessary for the synthesis of **5**) could not be, resulting in complex mixtures. Therefore it was necessary to change approach.

## Results & Discussion

### Mixed anhydride mediated peptide coupling

The mixed anhydride mediated peptide coupling is by no means a new reaction, with its first applications to peptide synthesis reported in the 1950’s [23] and 1960’s [24]. This approach to amide bond formation has the advantage of innocuous and easy to remove by-products and reasonable atom efficiency when compared to other peptide coupling agents, although it does require the use of a stoichiometric base as part of the reaction.

As the standard approach to carrying out a mixed anhydride mediated amide bond formation involves the formation of the mixed anhydride at low temperature before warming to rt during the coupling step, this seemed well matched to the flow setup we had already employed for our acid chloride mediated coupling (Scheme 2), and therefore this setup was used as our starting point. Initially Boc-Trp(CHO)-OH (**9**) (0.01 M) and two equivalents of diisopropylethylamine were filled into sample loop 1, ethyl chloroformate (1 equivalent) was employed as the coupling agent in sample loop 2, and a further two equivalents of base and Ala-OBn⋅HCl (**10**) were filled in to loop 3. Streams 1 and 2 were pre-cooled to −10 °C (3 min residence time) before meeting at a Y-piece and reacting at -10 °C for five minutes to form the mixed anhydride coupling partner. Stream 3 was pre-cooled for 2.5 min at −10 °C before it met the mixed anhydride stream at a further Y-piece, after with the reaction stream was warmed to rt for a further 6.67 min. The outflow was quenched by dripping into a stirred flask of aqueous potassium bisulfate (10%) at rt, with aqueous workup and purification being carried out using standard batch processes once the entire plug had been collected (Table 1). These initial conditions, resulted in a 64% yield of dipeptide **11**, which had not been accessible using Ghosez’s reagent (entry 1, Table 1). Changing to using *N*-methylmorpholine (NMM) as the base was seen to increase the yield to 85% (entry 2, Table 1), and a temperature screen using NMM as the base showed that the best yield (99%) was obtained when the mixed anhydride formation was carried out at 20 °C (entries 2–6, Table 1), in contrast to the reduced temperature usually required for the activation step in reported batch conditions [24, 25]. As isobutyl chloroformate (IBCF) has been shown to be a superior to ethyl chloroformate for amide bond formation [23, 24] this was next investigated, with it being seen that using IBCF with anhydride formation at −10 °C, 99% yield of **11** could be obtained (entry 7, Table 1). Next, the effect of reaction stream concentration was analysed, with a series of higher concentrations up to 0.1 mol L^−1^ investigated, however it was found that at the higher concentrations the yields were significantly reduced (entries 7 – 10, Table 1). Interestingly however, it was found that if the flow rate was doubled (and accordingly the respective residence times halved) at 0.1 mol L^−1^ there was no reduction in yield (entry 11, Table 1), indicating that there was scope to further optimise the parameters for the coupling.

**Table 1:**
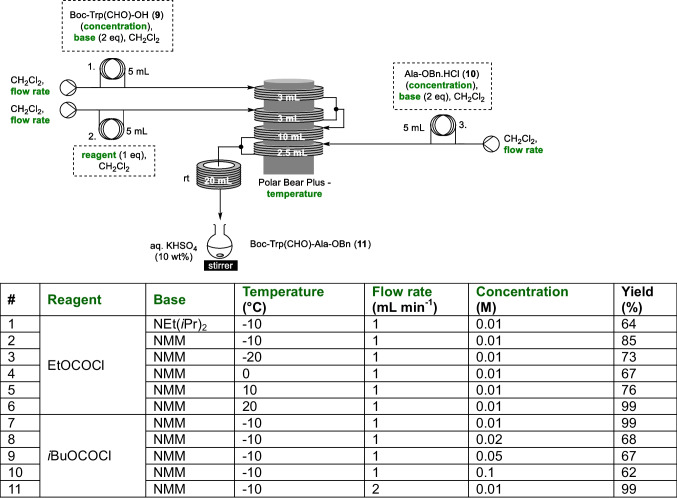
Preliminary conditions scan for mixed anhydride mediated dipeptide synthesis with the optimised parameters shown in green

An inherent advantage offered by flow chemistry is the ability to rapidly and independently optimise the formation of reactive intermediates employing in-line detection. Using the coupling of the more robust amino acids, Cbz-Phe-OH (**12**) and Val-OMe⋅HCl (**13**) to give dipeptide Cbz-Phe-Val-OMe (**14**), it was decided to independently optimise the two steps of the peptide coupling.

**Scheme 3:**
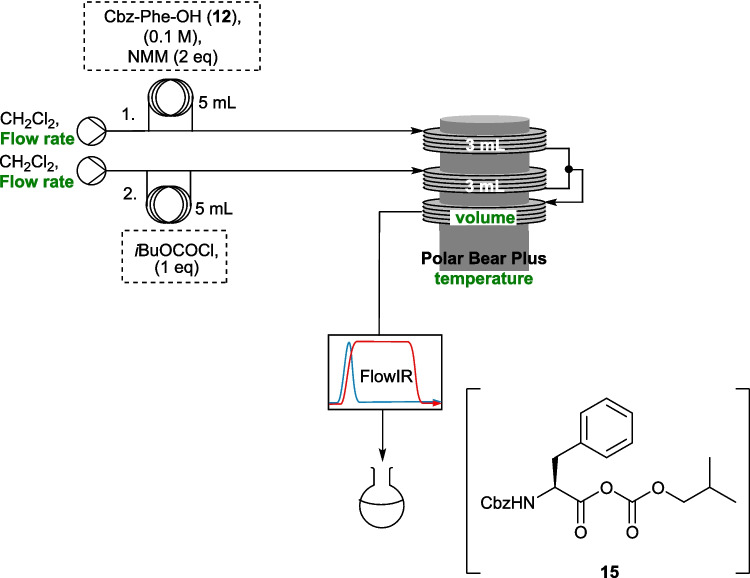
Optimisation of residence time for formation of mixed anhydride **15** using in-line IR detection with optimised variables shown in green

A FlowIR (Mettler-Toledo) was used to record the IR spectrum of the reaction mixture immediately after the third reaction coil, as shown in Scheme 3. The flow rate of each pump was varied in 0.5 mL min^−1^ steps from 0.5 mL min^−1^ to 4 mL min^−1^, monitoring the height of the peak at 1827 cm^−1^ (which was present in mixed anhydride **15** but not in the starting materials). It was observed that there was no significant change in the height of this peak between the different flow rates, which represented residence times for mixed anhydride formation of between 10 min and 1.25 min, indicating that the activation step occurs rapidly. Accordingly, the activation coil was reduced in volume to 2.5 mL during the further optimisation. Next, the temperature for the activation step was evaluated in a similar fashion, with temperatures between −20 °C and 25 °C evaluated in steps. It was observed that formation of **15** was consistent across all temperatures, and therefore it was decided to carry out the reaction at rt, allowing the flow setup to be significantly simplified without the requirement for pre-cooling coils, or active cooling. In batch reactions the yield is reported to decrease with increasing temperature, at least in part due to the competing urethane formation [23–26], however no evidence of this was observed by IR.

With simplified activation conditions established, attention next turned to the optimisation of the coupling step (Table 2). Rather than using an offline aqueous workup, in-line work up was attempted using a column packed with 10 equivalents of immobilised sulfonic acid (QP-SA) to remove any residual base and a column packed with 10 equivalents of immobilised dimethylamine (QP-DMA) to remove any residual IBCF. Comparison between the ^1^H NMR spectrum of the crude system outflow without a quench and when the scavenging columns were used (see Figure ESI3) demonstrated that further purification was necessary if the reaction had not gone to completion, so the resulting crude products were routinely purified by flash column chromatography to give isolated yields. A 40 psi back pressure regulator was included to help prevent cavitation in the dichloromethane reaction stream at rt. Initially, a flow rate of 1 mL min^−1^ per pump was used, to give a residence time for the coupling of 6.67 min, which was seen to result in just 60% yield of **14** (entry 1, Table 2). When the flow rate was reduced to 0.33 mL min^−1^ per pump (residence time of 20 min) it was seen that yield increased further to 83% (entry 2, Table 2), and while a modest increase in yield could be obtained with this residence time by heating the reaction coil to 40 °C (92%), increasing the volume of the reactor coil from 20 to 30 mL (30 min residence time) allowed **14** to be formed in an excellent 98% yield without the requirement of heating (entry 3 and 4, Table 2). It should be noted that when this same coupling has been carried out previously in our group under standard batch conditions using ICBF, a 42% yield of dipeptide **14** was obtained (see ESI Method C).

**Table 2:**
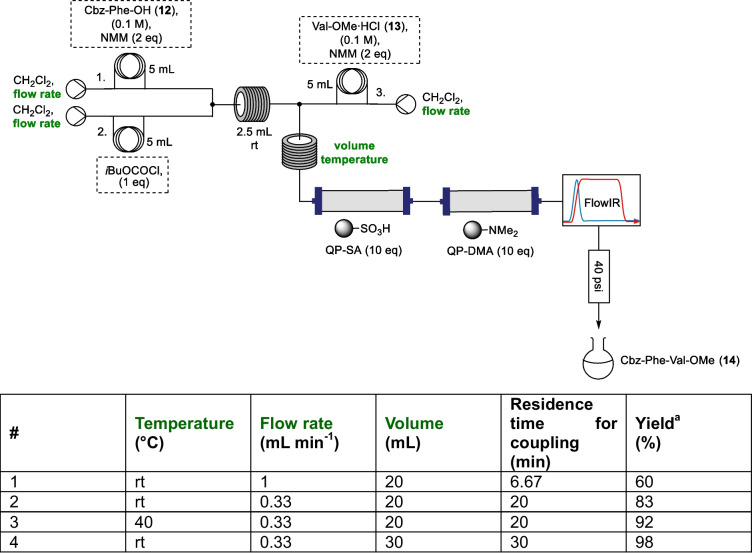
Optimisation of coupling conditions for dipeptide **14** with the optimised parameters shown in green

^a^Isolated yield after flash column chromatography

With optimised conditions in hand. The application of this to peptide couplings with a range of coupling partners was investigated on 0.5 mmol scale (plug flow). As initial tests showed that the in-line quench conditions would not be compatible with acid sensitive Boc protecting groups, for the majority of examples aqueous workup was used in place of the in-line work up with the solid supported reagents (Table 3).

**Table 3:**
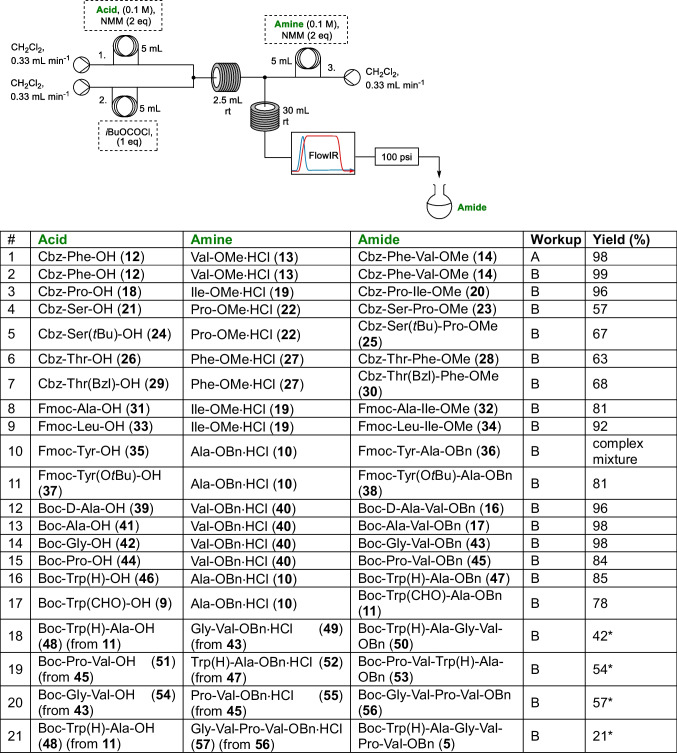
Scope evaluation for mixed anhydride mediated peptide coupling on 0.5 mmol scale using plug flow

Workup:

A-Purified yield after in-line workup with successive columns containing 10 eq of QP-SA and 10 eq of QP-DMA then flash column chromatography (see Table 2)

B-Purified yield after aqueous work up and flash column chromatography

*Yield includes the deprotection of the coupling partners as detailed in the ESI

It was observed that all three amine protecting groups evaluated under these conditions (Cbz, Boc and Fmoc) could be well tolerated. While unsurprisingly for serine (entries 4 vs 5, Table 3), threonine (entries 6 vs 7, Table 3) and tyrosine (entries 10 vs 11, Table 3) residues it was seen that protecting the amino acid side chain resulted in higher yields for the coupling, of particular relevance for the synthesis of segetalin precursor **5**, it was seen that unprotected tryptophan coupled in higher yield than when the indole amine was protected with a formyl group (entries 16 vs 17, Table 3).

The successful synthesis of Boc-D-Ala-Val-OBn (**16**) and Boc-Ala-Val-OBn (**17**) in 96% and 98% yield respectively (entries 12 and 13, Table 3), allowed investigation of whether racemisation was being observed during the process. HPLC comparison of the crude reaction mixtures (after aqueous work up) and purified dipeptides **16** and **17** (after flash column chromatography) with a sample containing both **16** and **17** determined that no evidence of isomerisation products could be seen (see Figure ESI4), which was also supported by comparison of the ^1^H NMR spectra (see Figure ESI5).

**Scheme 4:**
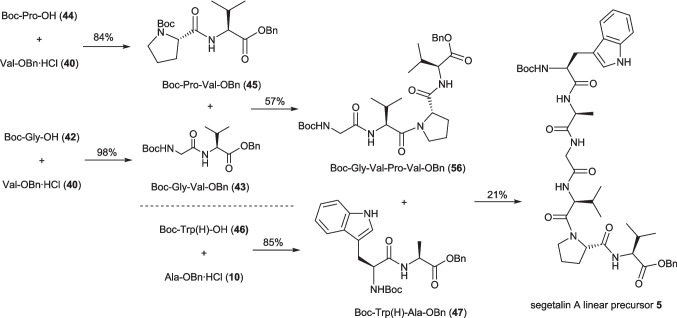
Plug flow (0.5 mmol scale) synthesis of segetalin A linear precursor **5** using the coupling conditions reported in Table 3

With attention next focusing on the synthesis of the desired segetalin A precursor **5**, the plug flow reactions enabled us to rapidly determine an appropriate pathway for this hexapeptide (Scheme 4). As both Boc and benzyl esters were well tolerated in the developed IBCF flow coupling conditions, we were able to employ the same protection strategy as we had in our previous depsipeptide synthesis[16] which allowed for the straightforward removal of the *C*-terminus benzyl ester protection by hydrogenation and anhydrous acid mediated Boc deprotection of the *N*-terminal where required (see ESI). **5** was broken down into three dipeptides, Boc-Gly-Val-OBn (**43**), Boc-Trp(H)-Ala-OBn (**47**) and Boc-Pro-Val-OBn (**45**). The coupling of the three possible combinations of dipeptides was successful to form three tetrapeptides (**50**, **53** and **56**), with the *N*-terminus of Boc-Gly-Val-Pro-Val-OBn (**56**) subsequently deprotected and coupled with *C*-terminus deprotected Boc-Trp(H)-Ala-OBn (**47**) to afford the desired hexapeptide** 5**, albeit in modest 21% yield.

As the yield was observed to decrease significantly for the tetrapeptide synthesis it seemed worthwhile to do some investigation into whether this yield could be improved, with the synthesis of tetrapeptide **56** used to determine whether these yields could be improved by varying the residence times of the two steps of the reaction (Table 4). Initially the volume of the reaction coil for the activation step was increased from 2.5 mL to 10 mL, increasing the residence time for the activation from 3.8 min to 15 min while leaving the coupling step residence time unchanged (entry 2, Table 4). This was seen to decrease the yield to 34% from 57%. When the overall flow rate was decreased to 0.15 mL min^−1^ pump^−1^, increasing the two residence times to 5.6 min and 66.67 min respectively (from 3.8 min and 30 min) minimal change in yield was observed (entries 1 vs 3, Table 4). Finally, it was seen that increasing the volume of the reaction coil for the coupling step only also did not lead to a significant improvement in yield (entry 4, Table 4).

**Table 4:**
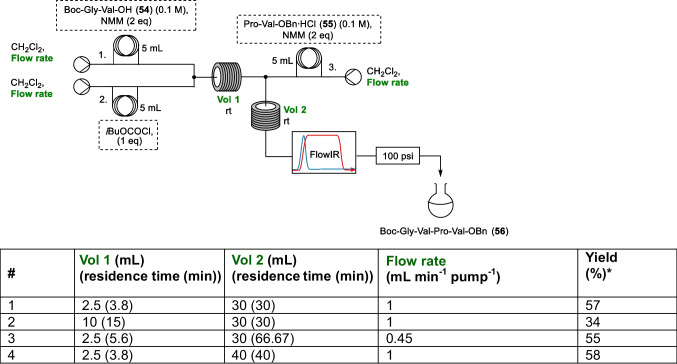
Optimisation of the coupling to afford tetrapeptide **56** with optimised parameters shown in green

* Yield includes the deprotection of the coupling partners as detailed in the ESI

With plug flow conditions established, attention next turned to whether these reactions could be carried out in continuous flow to enable scaling. Three streams were flowed at 0.33 mL min^−1^ using peristaltic pumps, with the timings of moving between solvent (CH_2_Cl_2_) and the reaction streams determined so as that they met at the appropriate Y-pieces. Accordingly, a 0.1 M solution of the acid coupling partner (formed by the hydrogenation of the appropriate benzyl ester precursor where relevant) and 2 equivalents of NMM in CH_2_Cl_2_ met a stream of IBCF (0.1 M) in CH_2_Cl_2_ at a Y-piece, and passed through a 2.5 mL reaction coil at rt before meeting a stream of the amine coupling partner (formed by the acid catalysed deprotection of the appropriate Boc precursor where relevant) which then passed through a 30 mL rt reaction coil, an in-line IR detector and a 100 psi back pressure regulator. For all but the hexapeptide synthesis this reaction was carried out on 10 mmol scale, with the streams switched back to CH_2_Cl_2_ once each solution had passed into the flow machine, allowing collection of the entire plug (guided by FlowIR). Once the full plug was collected, this underwent off-line aqueous workup and purification as carried out for the plug flow reactions (Table 5).

**Table 5:**
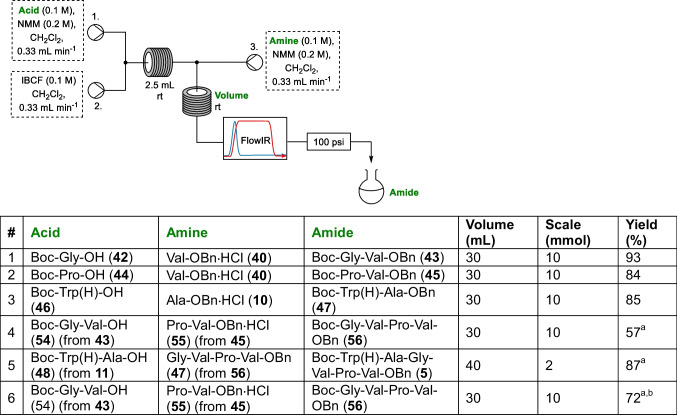
Continuous flow synthesis of segetalin linear precursor **5**

^a^Yield includes the deprotection of the coupling partners as detailed in the ESI

^b^The reaction outflow was collected under an inert atmosphere and stirred at rt for an additional 18 h before aqueous workup

While for the synthesis of the three dipeptides and the tetrapeptide the yield between the plug and continuous flow reactions was very similar, it was noted that the isolated yield for the coupling to form the hexameric linear precursor was dramatically improved from the plug flow yield of 21% to 87% when this reaction was carried out on 2 mmol scale continuously, affording 1.2 g of segetalin linear precursor **5**. While part of this increase in yield could be attributed to the increase in reactor volume for the coupling step between the plug (30 mL) and the continuous flow (40 mL), it should also be noted that the reaction outflow was only being worked up once the whole reaction plug had been collected. When the reaction was carried out on a larger scale this means that the effective residence time is significantly longer for the start of the reaction plug, with the 60 mL reaction (2 mmol with a final concentration of 0.033 M) taking 60 min to pass through the flow setup with the overall flowrate of 1 mL min^−1^. While it appears that this effective increase in reaction time does not significantly improve the yields for dipeptides **43**, **45** and **47** or tetrapeptide **56**, even with the 10 mmol reaction scale (300 mL reaction plug), this could be contributing to the improved yield of **5**. While we had not seen an increase in yield for a modest increase in residence time for the coupling to form tetrapeptide **56** (Table 5), as this could be due to the slow relative rate of reaction with the secondary amine of proline in the coupling step, it seemed sensible to take advantage of this continued reactivity to determine whether we could increase the yield for this key coupling by using a hybrid batch/flow approach to its synthesis. Accordingly, the coupling of **54** and **55** was run on 10 mmol scale, with the outflow collected into a flask under an argon atmosphere and stirred overnight before workup was carried out. This hybrid approach was seen to increase the yield to 72% (entry 6, Table 5).

**Scheme 5:**
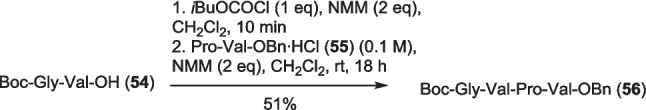
Batch synthesis of tetrapeptide **56** using IBCF

With the success of the flow/batch hybrid approach for this step, it seemed wise to determine whether carrying out this reaction in flow was giving any advantage, or whether the same results could be obtained in batch. Accordingly, the conditions were replicated as closely as possible in batch for a large scale reaction (17.7 mmol), with a solution of IBCF (0.1 M in CH_2_Cl_2_) added via cannula to a stirring solution of Boc-Gly-Val-OH (**54**) (0.1 M) and NMM (0.2 M) in CH_2_Cl_2_ and the reaction mixture stirred at rt for 10 min before a solution of Pro-Val-OBn⋅HCl (**55**) (0.1 M in CH_2_Cl_2_) was added via cannula and the resulting reaction mixture stirred at rt overnight. After the standard workup was carried out, the desired tetrapeptide **56** was isolated in a reduced 51% yield (Scheme 5). The batch reaction on this scale proved to be challenging operationally, with the necessity to transfer large volumes between flasks leading to prolonged addition times, which is especially relevant for the activation step, where we have already demonstrated that prolonged reaction times can lead to reduction in yield (e.g. entry 2, Table 4). Additionally, the greater complexity of the crude reaction mixture (compared to the flow reaction) indicated that under these conditions some racemisation or other side reactions could be occurring, presumably due to poorer heat or stoichiometry control.

Utilising a hybrid approach for this coupling allows us to benefit from with best of both worlds, with the flow machine controlling the combination of the streams so as the ideal stoichiometry is maintained easily, and providing a large relative surface area for heat dissipation, while the desired relative reaction times for the two steps can be achieved without having to use prohibitively long reaction coils or by decreasing the flow rate significantly (and therefore throughput of the system).

## Conclusions & outlook

**Scheme 6:**
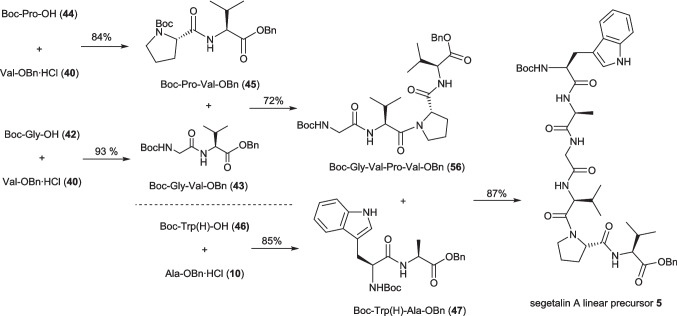
Successful continuous flow synthesis of segetalin A linear precursor **5** using the coupling conditions reported in Table 5

We have established conditions for a solution phase flow amide coupling at rt using IBCF as the coupling agent and successfully applied this to the coupling of a variety of amino acids containing different protecting groups to make a series of dipeptides. Employing a Boc/Benzyl ester protection strategy the developed coupling conditions were then applied to the synthesis of 1.2 g of hexapeptide **5**, the linear precursor for bioactive natural product segetalin A (**4**). The solution phase nature of the reaction enables a highly convergent (3 step longest linear) approach to segetalin A linear hexapeptide precursor **5**. The developed approach was successful both in plug flow and continuous flow modes (< 10 mmol scale), making it suitable for the synthesis of synthetically useful amounts of peptides, with activation times of 3.8 min and coupling times of 30–40 min leading to yields for the coupling step (and any necessary deprotections) between 84 and 98% on scale for couplings involving primary amine containing amino acids. In the case of the synthesis of tetrapeptide **56**, where secondary amine containing proline is the nucleophilic coupling partner, it was found that the slow rate of reaction could be compensated for by using a hybrid flow/batch approach, where the reactor outflow was collected and stirred overnight under an inert atmosphere before quenching to increase the yield (Scheme 6).

While excesses base (NMM) was necessary for the reaction to proceed, stoichiometric amount of both the coupling partners and ICBF were used, minimising waste, affording the target hexapeptide **5** in 52% yield (lowest yielding longest linear route, Scheme 6). Pleasingly, despite carrying out this reaction at rt, no evidence of racemisation was seen with our optimised conditions.

## Supplementary Information

Below is the link to the electronic supplementary material.

Electronic supporting information including full experimental details, characterization of products and supporting NMR spectra is provided. (PDF 3641 KB)
