# Supplementary material for: Straightforward, scalable, solution-phase synthesis of peptide bonds in flow
Source: J Flow Chem. 2025 Mar 12;15(2):67–77. doi: 10.1007/s41981-025-00347-2 (PMC12181218; doi:10.1007/s41981-025-00347-2)
Supplement: Supplementary file 1 — Electronic supporting information including full experimental details, characterization of products and supporting NMR spectra is provided. (PDF 3641 KB) [file 41981_2025_347_MOESM1_ESM.pdf]

Electronic Supporting Information

Straightforward, Scalable, Solution-Phase Synthesis of Peptide Bonds in Flow.

Zoe E. Wilson*, Enol Lopez, Nils J. Flodén, Charis Watkins, Giulia Bianchini and Steven V. Ley.

Department of Chemistry, University of Cambridge, Lensfield Road, Cambridge (United Kingdom).

Table of Contents

General Details 3

Methods 4

Method A: Preliminary Conditions Scan 4

Method B: Optimisation of coupling conditions using the synthesis of dipeptide **14** 5

Method C. Batch synthesis of Cbz-Phe-Val-OMe (**14**) 5

Method D: Plug flow peptide coupling (0.5 mmol scale) 6

Method E: Deprotection of benzyl ester 7

Method F: Deprotection of Boc protected amine 7

Method G: Optimisation of the coupling to afford tetrapeptide **56** 8

Method H: Continuous flow peptide coupling (mmol scale). 8

Method I: Hybrid synthesis of tetrapeptide **56.** 9

Method J: Batch synthesis of tetrapeptide **56.** 10

In-line quench investigation. 10

Evaluation of whether isomerisation is occurring under the conditions. 11

Yields and Characterisation 13

Boc-Trp(CHO)-Ala-OBn (**11**) 13

Cbz-Phe-Val-OMe (**14**) 14

Cbz-Pro-Ile-OMe (**20**) 15

Cbz-Ser-Pro-OMe (**23**) 15

Cbz-Ser(tBu)-Pro-OMe (**25**) 16

Cbz-Thr-Phe-OMe (**28**) 17

Cbz-Thr(Bzl)-Phe-OMe (**30**) 18

Fmoc-Ala-Ile-OMe (**32**) 19

Fmoc-Leu-Ile-OMe (**34**) 20

Fmoc-Tyr(OtBu)-Ala-OBn (**38**) 21
Boc-D-Ala-Val-OBn (**16**) 22
Boc-Ala-Val-OBn (**17**) 22
Boc-Gly-Val-OBn (**43**) 23
Boc-Pro-Val-OBn (**45**) 23
Boc-Trp(H)-Ala-OBn (**47**) 24
Boc-Trp(H)-Ala-Gly-Val-OBn (50) 25
Boc-Pro-Val-Trp(H)-Ala-OBn (**53**) 26
Boc-Gly-Val-Pro-Val-OBn (**56**) 27
Boc-Trp(H)-Ala-Gly-Val-Pro-Val-OBn (**5**) 28
NMR Spectra 29
Boc-Trp(CHO)-Ala-OBn (**11**) 29
Cbz-Phe-Val-OMe (**14**) 31
Cbz-Pro-Ile-OMe (**20**) 32
Cbz-Ser-Pro-OMe (**23**) 32
Cbz-Ser(tBu)-Pro-OMe (**25**) 33
Cbz-Thr-Phe-OMe (**28**) 35
Cbz-Thr(Bzl)-Phe-OMe (**30**) 36
Fmoc-Ala-Ile-OMe (**32**) 38
Fmoc-Leu-Ile-OMe (**34**) 40
Fmoc-Tyr(OtBu)-Ala-OBn (**38**) 42
Boc-D-Ala-Val-OBn (**16**) 42
Boc-Ala-Val-OBn (**17**) 43
Boc-Gly-Val-OBn (**43**) 43
Boc-Pro-Val-OBn (**45**) 44
Boc-Trp(H)-Ala-OBn (**47**) 44
Boc-Trp(H)-Ala-Gly-Val-OBn (**50**) 46
Boc-Pro-Val-Trp(H)-Ala-OBn (**53**) 48
Boc-Gly-Val-Pro-Val-OBn (**56**) 51
Boc-Trp(H)-Ala-Gly-Val-Pro-Val-OBn (**5**) 53
References 56

General Details

All reactions were carried out under argon atmosphere using oven-dried glassware, and were monitored by TLC. Unless otherwise stated, reagents were obtained from commercial sources and used without further purification. Amino acids were all of the natural (L) enantiomeric form unless otherwise stated. Solvents were freshly distilled over calcium hydride and lithium aluminium hydride (tetrahydrofuran and diethyl ether) or calcium hydride (dichloromethane, methanol, toluene, ethyl acetate and 40–60 petroleum ether). Additional anhydrous solvents were obtained from commercial sources and used directly (*N,N*-dimethylformamide).

Flow reactions were carried out using either Vapourtec R2+[1] (stainless steel or PTFE piston pumps used interchangeably) or Vapourtec E-series[2] (peristaltic pumps fitted with blue coded peristaltic tubing) and a Polar Bear Plus cooling unit[3] (Cambridge Reactor Design). In-line detection was carried out using a FlowIR[4] (Mettler Toledo) fitted with a diamond head. Sudan Red dye was utilised to determine the necessary injection times so that the flow streams met in a similar fashion to that previously employed in the Ley group.[5] The flow rates being delivered by the pumps were evaluated periodically by collecting the system out flow in a measuring cylinder over time, with the flow rates being adjusted according to the pump manufacturers instructions if necessary. Flow Tubing used was 1.59mm OD PTFE or PFA and components were connected by flat bottomed flangeless connectors made out of PEEK or EFTE (e.g. IDEX Health and Science part #P255 and #P-259) using standard PEEK or EFTE connectors (e.g. IDEX Health and Science part #P702). PEEK Y-pieces (e.g. IDEX Health and Science part #P-512) were used to connect streams, and back pressure regulators used were fixed pressure BPRs (40 psi - IDEX Health and Sciences part #P-785, 100 psi – IDEX Health and Sciences part #P-787).

Thin layer chromatography (TLC) was carried out using 0.25 mm thick glass backed Merck TLC Silica gel 60 F254 plates which were visualised using ultraviolet radiation and aqueous acidic ammonium molybdate (VII) solution or potassium permanganate solution. Flash column chromatography was carried out using high-purity grade silica gel (Merck grade 9385) with a pore size 60 Å and 230–400 mesh particle size.

Infrared spectroscopy was recorded using a PerkinElmer Spectrum One FT-IR spectrometer using Universal ATR sampling accessories. Absorbance measurements were recorded in the range 4000–650 cm^{-1}.

NMR spectra were recorded using either a 400 MHz DPX-400 Dual spectrometer, a 500 MHz AV III HD Smart Probe spectrometer or a 600 MHz Avance 600 BBI spectrometer as indicated. Unless otherwise stated, all samples were run at room temperature (rt) in deuterated solvent, with chemical shift (δ) reported to the nearest 0.01 (^{1}H)/0.1 ppm (^{13}C), relative to the residual protic solvent; $\delta(CDCl_3)$=7.26 (^{1}H)/77.16 ppm (^{13}C) or δ([D_6]-DMSO) 2.50 (^{1}H)/39.51 ppm (^{13}C). All carbon NMRs were run with broadband proton decoupling. Multiplicity of a signal in ^{1}H NMR spectra is indicated by: s=singlet, d=doublet, t=triplet or, q=quartet, quint=quintet, m=multiplet, or a combination thereof. Multiplets are reported as the range of ppm values covered by the signals, otherwise the centre of the signal is given. Coupling constants (*J*) are quoted in Hz and recorded to the nearest 0.1 Hz.

HRMS was performed using either a Waters Micromass LCT Premier spectrometer or by using a Bruker Bioapex 47e FTICR spectrometer, using positive ESI+. Masses are given in *m*/*z* units.

Melting points (m.p.) were measured using a Stanford Research Systems OptiMelt automated melting point system using a gradient of 1 °C min^{-1}, and are uncorrected. Specific optical rotation

was recorded on a Perkin–Elmer Model 343 digital polarimeter, using a Na/Hal lamp set at 589 nm and with a path-length of 100 mm. All [α_D] values were measured using spectroscopy grade solvent at the specified concentration [g cm^{-3}] and temperature, with units of 10^{-1} cm^2 g^{-1}.

The compound numbering system used is based on IUPAC conventions. Each amino acid is numbered individually, starting from the carboxyl group and proceeding up the side chain. Position labels thus refer first to the individual amino acid, and then to the position on it, with the atom to be defined (C or H) underlined. Non-amino acid derived functionality is numbered based on the position from which it branches. The entire branch is thus given the label of the position from which it begins, appended with a prime, and each position within the chain is further denoted by a lowercase letter. The branched chain in the case of isoleucine has the shorter branch also labelled with a prime according to the position at which it branches.

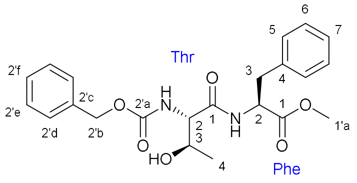

Cbz-Thr-Phe-OMe

Methods

Method A: Preliminary Conditions Scan

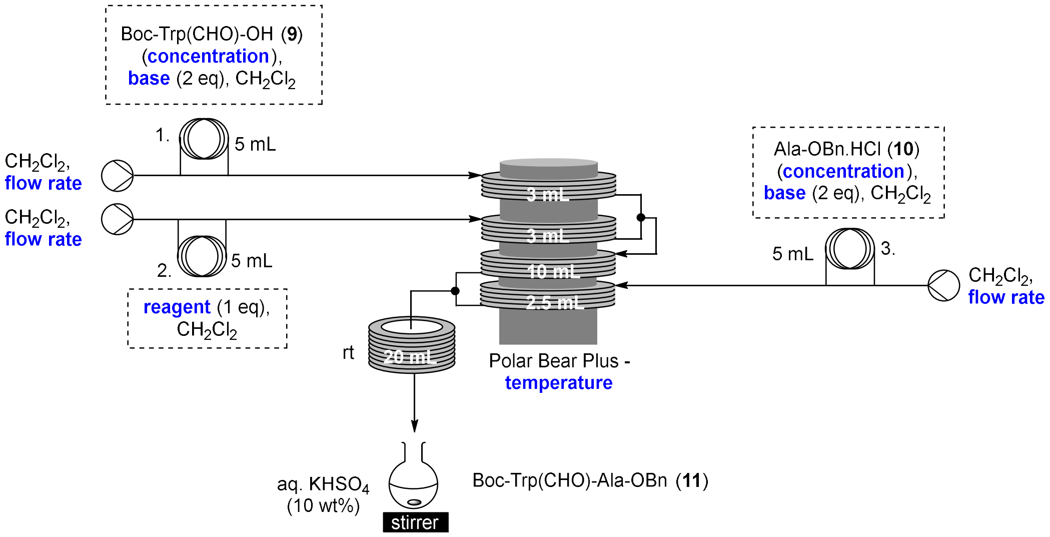

Scheme ESI1. Flow setup for the preliminary conditions scan for the mixed anhydride mediated dipeptide synthesis reported in table 1.

The flow setup employed for Table 1, using Vapourtec R series systems, is detailed in our previous publication.[6] Boc-Trp(CHO)-OH (**9**) (to give the required **concentration**) was taken up in dichloromethane (5 mL) and **base** (2 eq) was added before the resulting solution was filled into loop 1. **Reagent** (1 eq) was taken up in dichloromethane (5 mL) and was filled into loop 2. Ala-OBn·HCl (**10**) (to give the required **concentration**) was taken up in dichloromethane (5 mL) and **base** (2 eq) was added before the resulting solution was filled into loop 3. Pumps 1 – 3 were started pumping dichloromethane at **flow rate**. Loops 1 and 2 were injected so that they met at a Y-piece after passing through two pre-cooling loops (3 mL coils, **temperature**) and were allowed to react in a reactor coil (10 mL coil, **temperature**). Meanwhile, loop 3 was injected so as that it met the outflow after precooling (2.5 mL coil, **temperature**) and the combined streams

were allowed to warm to rt in a final reaction coil (20 mL). The reaction was quenched by dripping the output solution into stirring 10 % aqueous potassium hydrogen sulphate (10 mL). The phases were separated and the aqueous phase extracted with dichloromethane (3 x 10 mL). The combined organic phases were washed with water (10 mL), aqueous sodium hydrogen carbonate (10%, 10 mL) and brine (10 mL), dried over magnesium sulphate and concentrated *in vacuo* before purification of the desired product *via* flash column chromatography.

Method B: Optimisation of coupling conditions using the synthesis of dipeptide 14

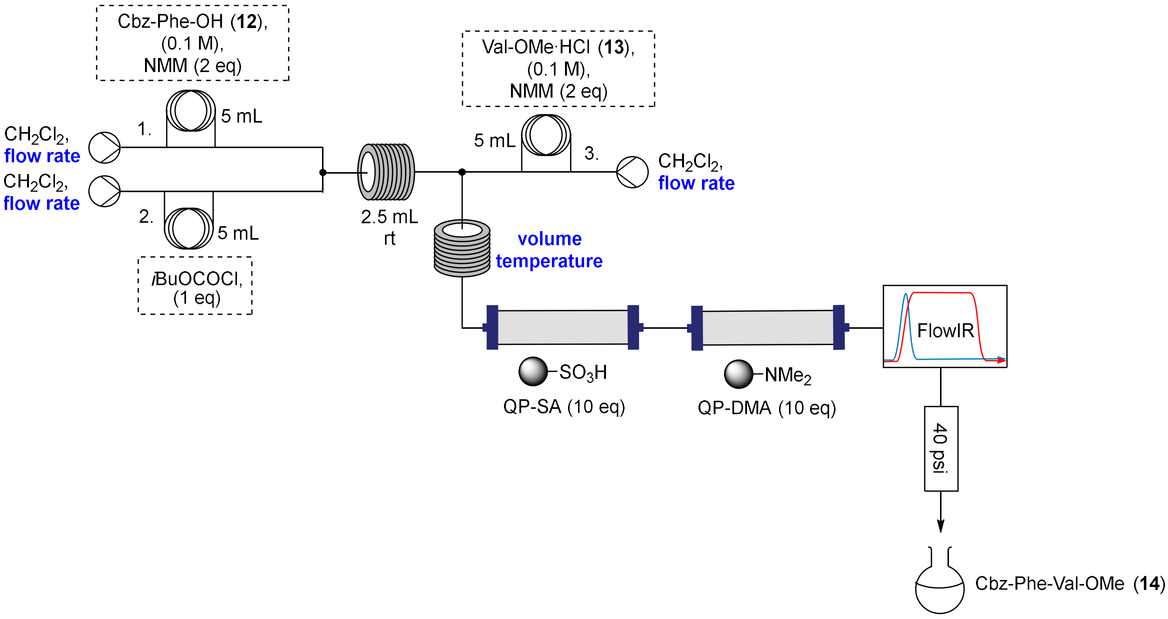

Scheme ESI2. Flow setup for the optimisation of the synthesis of dipeptide 14 reported in table 2.

The flow machine was set up according to Scheme ESI2 using Vapourtec R series systems. A solution of Cbz-Phe-OH (**12**) (0.143 g, 0.48 mmol) and NMM (0.11 mL, 0.96 mmol) in CH_2Cl_2 (5 mL) was loaded into sample loop 1, and a solution of isobutyl chloroformate (0.07 mL, 0.48 mmol) in CH_2Cl_2 (5 mL) was loaded into sample loop 2. The two loops were switched inline at **flow rate** so as they met at a Y-piece and the mixed stream passed through a 2.5 mL coil at rt. Meanwhile a solution of Val-OMe.HCl (**13**) (0.084 g, 0.50 mmol) and NMM (0.11 mL, 0.96 mmol) in CH_2Cl_2 (5 mL) was loaded into sample loop 3, and switched inline at **flow rate** to meet the previously mixed stream at a second T-piece, after which the reaction stream was passed through a reaction coil (**volume**, **temperature**). The stream was then directed through a 10 mm diameter Omnifit column packed with QP-SA (2.1 g), followed by a 6.6 mm diameter Omnifit column packed with QP-DMA (0.8 g). The stream was then passed through a FlowIR and finally through a 100 psi back-pressure regulator before being collected. The Flow IR readings were used to inform the timings for product collection. The collected solvent was removed *in vacuo* before purification of the desired product *via* flash column chromatography.

Method C. Batch synthesis of Cbz-Phe-Val-OMe (14)

A solution of Cbz-Phe-OH (**4**) (0.698 g, 2.33 mmol) and NMM (0.23 mL, 2.5 mmol) in dry THF (10 mL) was cooled to -5 °C, and isobutyl chloroformate (0.35 mL, 2.4 mmol) was added. The mixture was stirred for 5 min, following which a cold suspension of Val-OMe.HCl (**5**) (0.406 g, 2.43 mmol) and NMM (0.23 mL, mmol) in 3:2 THF:DMF (6.25 mL) was added. The mixture was stirred at 0 °C for 2 h and then overnight at rt. Ethyl acetate (30 mL) was added to the resultant yellow

suspension, and the resulting organic layer washed successively with aqueous hydrochloric acid (1 M, 120 mL), aqueous sodium bicarbonate solution (60 mL) and saturated sodium chloride (50 mL). The organic layer was dried over $MgSO_4$ and the solvent removed *in vacuo* to give the crude product as a white solid. Purification by flash column chromatography using 40-60 petroleum ether:ethyl acetate (2:1, R_F = 0.33) afforded the *title compound* **14** (0.408 g, 0.99 mmol, 42%) as a colourless amorphous solid.

Method D: Plug flow peptide coupling (0.5 mmol scale)

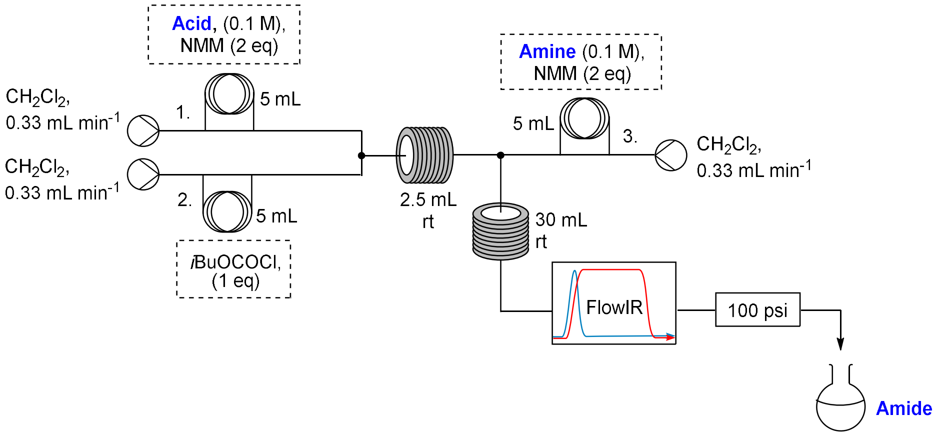

Scheme ESI3. Flow equipment set up for plug flow (0.5 mmol) peptide coupling general method.

The flow machine was set up according to Scheme ESI3 and Figure ESI1 using Vapourtec R series systems. A solution of **acid**[A] (0.5 mmol) and *N*-methyl morpholine (0.11 mL, 1.0 mmol) in CH_2Cl_2 (5 mL) was loaded into sample loop 1 and a solution of isobutyl chloroformate (0.077 mL, 0.6 mmol) in CH_2Cl_2 was loaded into sample loop 2. The two were switched inline at a flow rate of 0.33 mL min^{-1} so that the two streams mixed at a T-piece and the mixed stream passed through a 2.5 mL coil (3.7 min residence time) at rt. Meanwhile a solution of **amine**[B] (0.5 mmol) and *N*-methyl morpholine (0.11 mL, 1.0 mmol) in CH_2Cl_2 (5 mL) was loaded into sample loop 3 and was switched inline at a flow rate of 0.33 mL min^{-1} to meet the previously mixed stream at a second T-piece, after which the reaction stream was passed through a 30 mL (30 min residence time) coil at rt. The stream was then directed through a FlowIR and finally through a 100 psi back-pressure regulator before the plug was collected guided by FlowIR. The collected reaction mixture was washed with aqueous HCl (1M, 30 mL), saturated sodium bicarbonate (30 mL) and water (30 mL) before drying over $MgSO_4$. The solvent was removed *in vacuo* and the resultant crude product purified by flash column chromatography to give the desired amide.

Notes:

A. Where indicated the **acid** was formed by hydrogenation of the benzyl ester in the precursor peptide according to Method E.
B. Where indicated the **amine** was formed by acid catalysed Boc deprotection of the precursor peptide according to Method F.

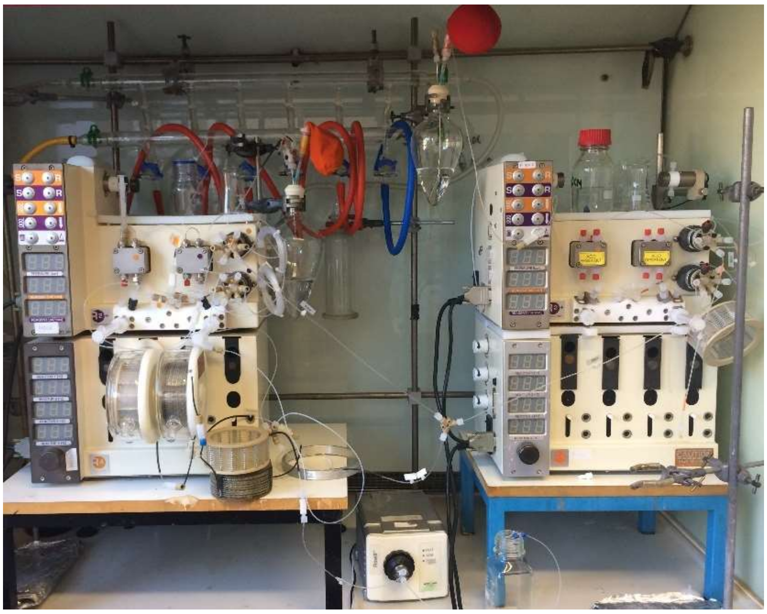

Figure ESI1. Photograph of flow chemistry setup used for Method D.

Method E: Deprotection of benzyl ester

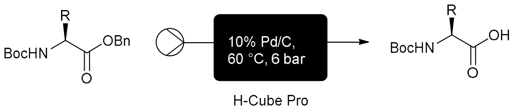

Scheme ESI4. Deprotection of C terminus.

A solution of protected precursor (0.167M in dichloromethane) was hydrogenated using an H-Cube® Pro (ThalesNano) with a $PdOH_2$ (20% on carbon) CatCart®. The pump was run at 1 mL min^{-1} using dichloromethane with the temperature set to 60 °C and the pressure to 6 bar. The solvent was then removed *in vacuo* and the resulting crude **acid** was used directly in the coupling reaction without further purification.

Method F: Deprotection of Boc protected amine

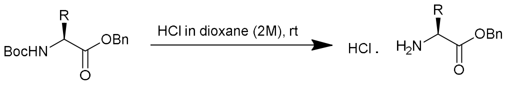

Scheme ESI5. Deprotection of N-terminus.

Anhydrous HCl (4 M in dioxane) was added to a solution of the protected peptide (0.1 M in dioxane) to give an overall 2M HCl solution. The resulting reaction mixture was stirred at rt for 3 h. The solvent was then removed *in vacuo* and the crude oil was taken up in ethanol (2 × 1 mL per mmol peptide) and methanol (1 mL per mmol peptide) successively, removing the solvent *in vacuo* after each addition, to afford the **amine** as the HCl salt, which was used directly in the coupling reaction without further purification.

Method G: Optimisation of the coupling to afford tetrapeptide 56.

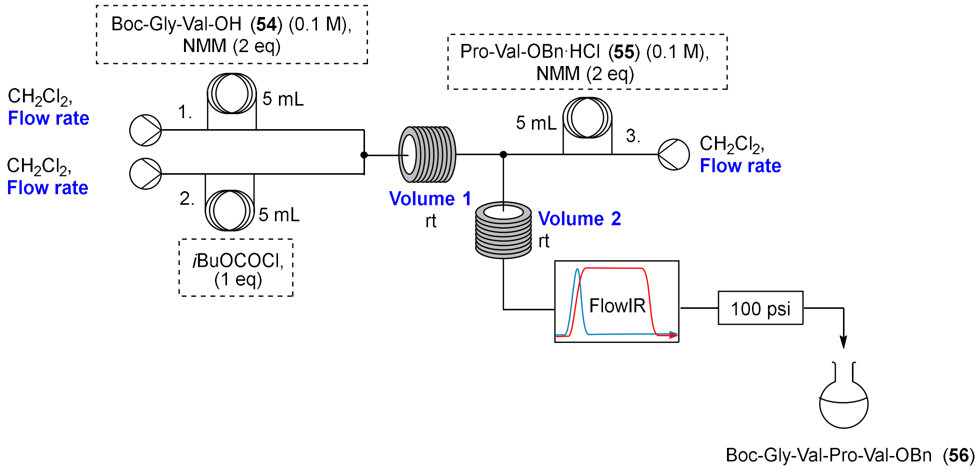

Scheme ESI6. Flow equipment setup for optimisation of coupling to form Boc-Gly-Val-Pro-Val-OBn (**56**).

The flow machine was set up according to Scheme ESI6 using Vapourtec R series systems. A solution of Boc-Gly-Val-OH (0.5 mmol, formed from Boc-Gly-Val-OBn (**43**) *via* Method E) and *N*-methyl morpholine (0.11 mL, 1.0 mmol) in CH_2Cl_2 (5 mL) was loaded into sample loop 1 and a solution of isobutyl chloroformate (0.077 mL, 0.6 mmol) in CH_2Cl_2 was loaded into sample loop 2. The two were switched inline at the stated **flow rate** so that the two streams mixed at a T-piece and the mixed stream passed through a reaction coil of the stated **volume 1** at rt. Meanwhile a solution of Pro-Val-OBn·HCl (0.5 mmol, formed from Boc-Pro-Val-OBn (**45**) *via* Method F) and *N*-methyl morpholine (0.11 mL, 1.0 mmol) in CH_2Cl_2 (5 mL) was loaded into sample loop 3 and was switched inline at the stated **flow rate** to meet the previously mixed stream at a second T-piece, after which the reaction stream was passed through a reaction coil of the stated **volume 2** at rt. The stream was then directed through a FlowIR and finally through a 100 psi back-pressure regulator before the plug was collected guided by FlowIR. The collected reaction mixture was washed with aqueous HCl (1M, 30 mL), saturated sodium bicarbonate (30 mL) and water (30 mL) before drying over $MgSO_4$. The solvent was removed *in vacuo* and the resultant crude product purified by flash column chromatography to afford Boc-Gly-Val-Pro-Val-OBn (**56**).

Method H: Continuous flow peptide coupling (mmol scale).

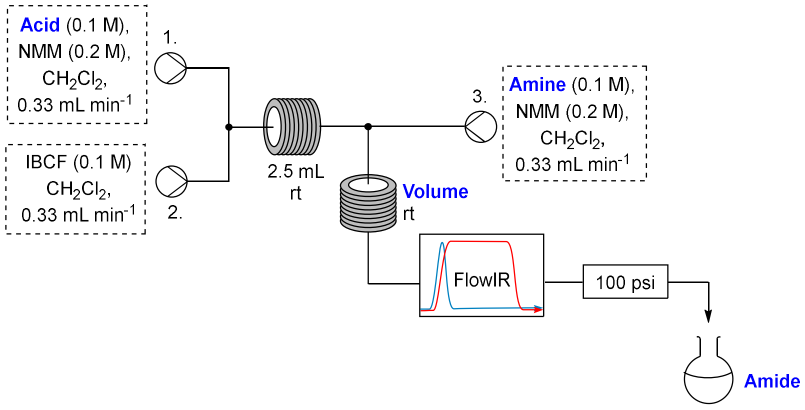

Scheme ESI7. Flow equipment set up for continuous flow peptide coupling general method.

The flow machine was set up according to Scheme ESI7 and Figure ESI2. Three peristaltic pumps (Vapourtec E series) were pumped at 0.33 mL min^{-1} using CH_2Cl_2. Solution 1, a solution of **acid**[A] (0.1 M) and *N*-methylmorpholine (0.2 M) in CH_2Cl_2, and solution 2, a solution of isobutyl chloroformate (0.11 M in CH_2Cl_2) were switched inline at this flow rate so as that the two streams mixed at a T-piece and the mixed stream passed through a 2.5 mL coil at rt. Meanwhile a solution of the corresponding **amine**[B] (0.1 M) and *N*-methylmorpholine (0.2 M) in CH_2Cl_2 was switched inline to meet the previously mixed stream at the second T-piece, after which the reaction stream was passed through a reaction coil of the specified **volume** at rt. The stream was then directed through a FlowIR and finally through a 100 psi back-pressure regulator. Once the reagent reservoirs were empty the pumps were switched back to CH_2Cl_2 to allow collection of the entire plug (guided by FlowIR). The reaction mixture was then washed with a aqueous HCl (1M, 1 x same volume as plug), aqueous saturated sodium bicarbonate (1 x same volume as plug) and finally with water (1 x same volume as the plug). The organic phase was dried over $MgSO_4$ and the solvent removed *in vacuo* before purification by flash column chromatography.

Notes:

A. Where indicated the **acid** was formed by hydrogenation of the benzyl ester in the precursor peptide according to Method E.
B. Where indicated the **amine** was formed by acid catalysed Boc deprotection of the precursor peptide according to Method F.

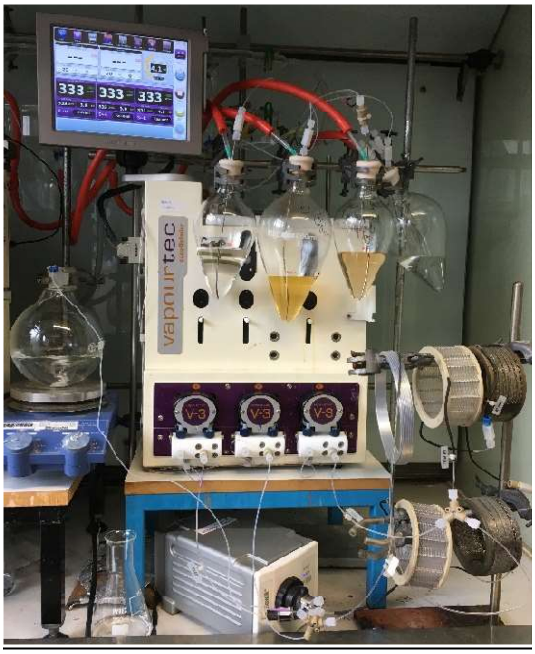

Figure ESI2. Photograph of flow chemistry setup used for Method H.

Method I: Hybrid synthesis of tetrapeptide 56.

Boc-Gly-Val-OH (10 mmol, formed from Boc-Gly-Val-OBn (**43**) *via* Method E) and Pro-Val-OBn·HCl (10 mmol, formed from Boc-Pro-Val-OBn (**45**) *via* Method F) were coupled according to Method H (**volume** = 30 mL) with the reaction outflow being collected in a stirring flask under argon. The reaction mixture was stirred at rt for 18 h after the final portion of the reaction mixture left the reactor before workup was carried out according to Method H.

Method J: Batch synthesis of tetrapeptide 56.

Boc-Gly-Val-OH (17.7 mmol, formed from Boc-Gly-Val-OBn (**43**) *via* Method E) and and stirred at rt. A solution of IBCF (2.75 mL, 21.24 mmol) in CH_2Cl_2 (170 mL) was added *via* cannula and the resulting mixture stirred for ten min after addition was complete before a solution of Pro-Val-OBn·HCl (17.7 mmol, formed from Boc-Pro-Val-OBn (**45**) *via* Method F) and NMM (3.9 mL, 35.4 mmol) in CH_2Cl_2 (170 mL) was added *via* cannula and the resulting solution stirred at rt for 18 h. The reaction mixture was then washed with aqueous HCl (1M, 170 mL), aqueous saturated sodium bicarbonate (170 mL) and finally with water (170 mL). The organic phase was dried over $MgSO_4$ and the solvent removed *in vacuo* before purification by flash column chromatography.

In-line quench investigation.

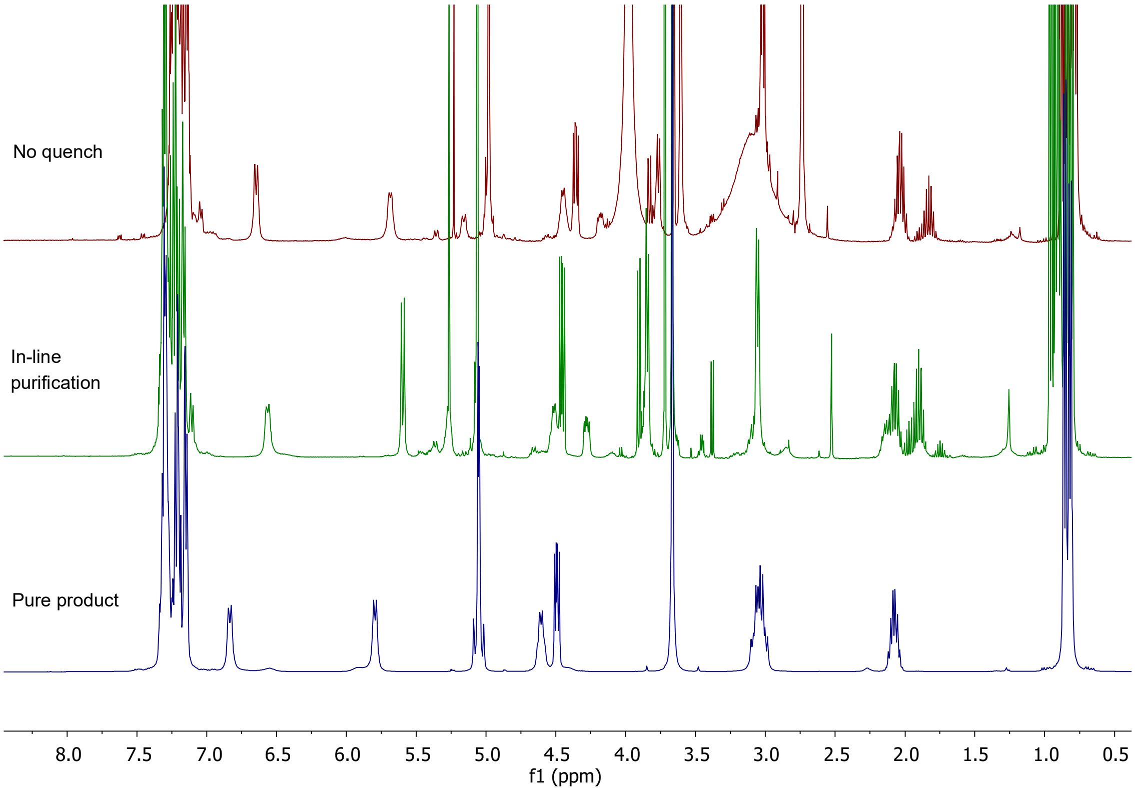

Figure ESI3: Crude 600MHz ^{1}H NMR in $CDCl_3$ showing the effect of in-line purification using QP-SA and QP-DMA columns for the coupling of Cbz-Phe-OH (**12**) and Val-OMe·HCl (**13**) using the conditions shown as entry 1 in Table 2. *Top:* when the outflow is concentrated *in vacuo* with no work-up (no quench), *Middle:* when the column is passed through QP-SA and QP-DMA columns (in-line purification), *Bottom:* the purified product.

Evaluation of whether isomerisation is occurring under the conditions.

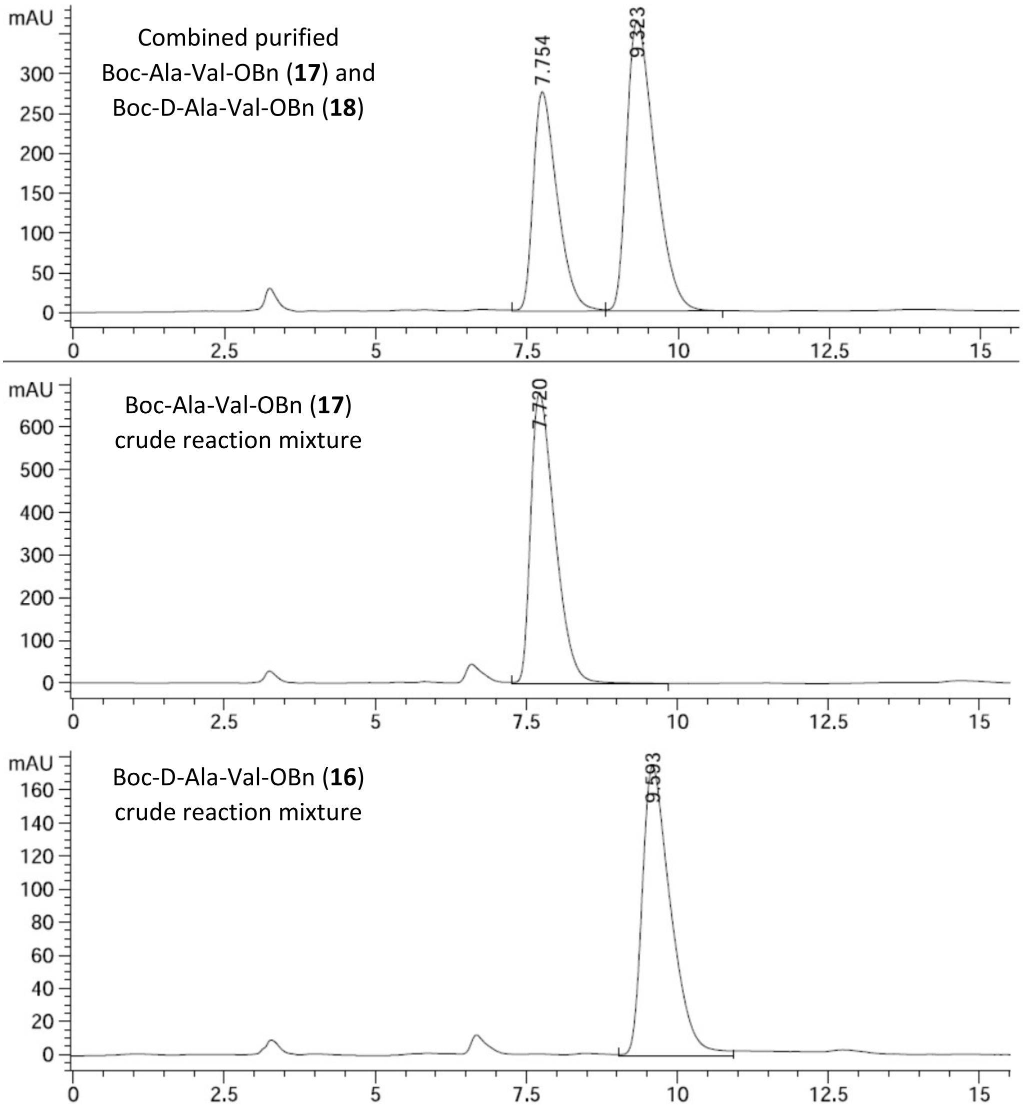

Figure ESI4. HPLC comparison of a mixture of purified Boc-Ala-Val-OBn (**17**) and Boc-D-Ala-Val-OBn (**16**) (*top*) with the crude reaction mixture from the synthesis of Boc-Ala-Val-OBn (**17**) (*middle*) and Boc-D-Ala-Val-OBn (**16**) (*bottom*) using Method D.

High Performance Liquid Chromatography was run on an Agilent Technologies 1100 Series HPLC monitoring at 260 and 254 nm, using a Chiralpack AD-H column at a flow rate of 0.75 mL min^{-1} using 9:1 Hexanes: isopropyl alcohol as eluent.

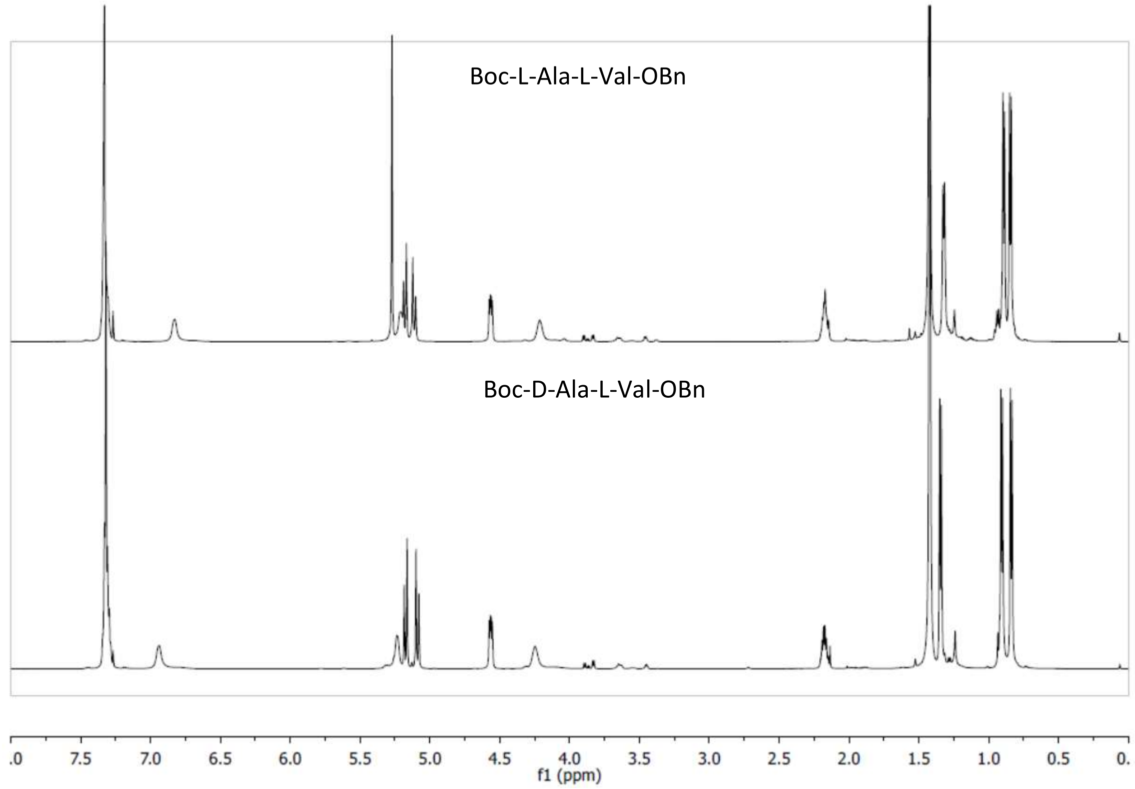

Figure ESI5. Crude ^{1}H NMR of the two dipeptides synthesised before purification.

Yields and Characterisation

Boc-Trp(CHO)-Ala-OBn (11)

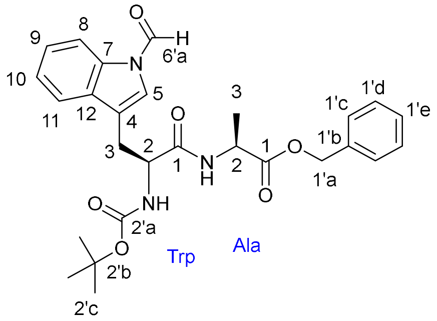

Yield: Method A = See Table 1, Method D = 78%.

Purification: flash column chromatography, 40-60 petroleum ether: ethyl acetate= 3:1, R_F = 0.31.

Description: colourless solid, mp = 137-139 °C.

Characterisation:

[α]$_D$ = -9.2 (*c* 1.00, MeOH).

^{1}H NMR (600 MHz, $CDCl_3$) δ 9.35 (s, 0.3H, Trp-C6’aH), 8.97 (s, 0.6H, Trp-C6’aH), 8.38 (d, *J* = 7.9 Hz, 0.6H, Trp-C8H), 7.65 – 7.60 (m, 1.4H, Trp-C8H and Trp-C11H), 7.40 – 7.28 (m, 7.4H, Trp-C5H, Trp-C9H, Trp-C10H, 2 x Ala-C1’cH, 2 x Ala-C1’dH, Ala-C1’eH), 7.21 (s, 0.6H, Trp-C5H), 6.52 – 6.31 (m, 1H, Trp-NH), 5.20 (s, 1H, Ala-NH), 5.15 – 5.10 (m, 2H, Ala-C1’aH_2), 4.54 (p, *J* = 7.2 Hz, 1H, Ala-C2H), 4.51 – 4.44 (m, 1H, Trp-C2H), 3.23 – 3.12 (m, 2H, Trp-C3H_2), 1.42 (s, 9H, 3 x Trp-C2’cH_3), 1.32 (d, *J* = 7.1 Hz, 3H, Ala-C3H_3).

^{13}C NMR (151 MHz, $CDCl_3$) δ 172.2 (C, Ala-C1), 170.5 (C, Trp-C1), 159.2 (C, Trp-C6’a), 155.3 (C, Trp-C2’a), 135.2 (C, Ala-C1’b), 128.6, 128.5 and 128.1 (5 x CH, 2 x Ala-C1’c, 2 x Ala-C1’d and Ala-C1’e), 125.5 and 124.8 (2 x CH, Trp-C9 and Trp-C10), 123.9 (C, Trp-C7), 121.4 (C, Trp-C12), 119.1 (CH, Trp-C11), 116.1 (CH, Trp-C8), 109.6 (C, Trp-C4), 80.4 (C, Trp-C2’b), 67.2 (CH_2, Ala-C1’a), 54.1 (CH, Trp-C2), 48.2 (CH, Ala-C2), 28.3 (3 x CH_3 + CH_2, Trp-C2’c, Trp-C3), 18.3 (CH_3, Ala-C3).

IR (neat) v/cm^{-1} = 3329 (N-H), 2924 (C-H), 1734 (C=O formyl), 1712 (C=O carbamate), 1683 (C=O amide), 1654 (C=O amide).

HRMS (ESI) = found 494.2307; $[M + H]^+$ $C_{27}H_{32}N_3O_6$ requires 494.2286; Δ = 4.3 ppm.

Cbz-Phe-Val-OMe (14)

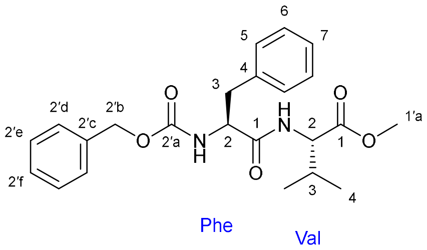

Yield: Method B= See table 2, Method C = 42%, Method D = 98%.

Purification: Flash column chromatography with 40-60 petroleum ether : ethyl acetate = 2:1, R_F = 0.30.

Description: white solid, mp = 106-108 °C, lit = 103-105 °C[7].

Characterisation:

$[\alpha]_D^{23.2}$: +11.8 (*c* 1.00, $CHCl_3$).

^{1}H NMR (600 MHz, $CDCl_3$) δ 7.47 – 7.08 (m, 10H, 10 x ArH), 6.24 (br s, 1H, Phe-NH), 5.33 (br s, 1H, Val-NH), 5.14 – 5.09 (m, 2H, Phe-C2'bH), 4.47 – 4.45 (m, 2H, Val-C2H and Phe-C2H), 3.71 (s, 3H, Val-C1'aH_3), 3.15 – 3.06 (m, 2H, Phe-C3H_2), 2.10 (dd, *J* = 12.4, 6.8 Hz, 1H, Val-C3H), 0.84 (dd, *J* = 22.6, 6.8 Hz, 6H, 2 x Val-C4H_3). The ^{1}H NMR data was in agreement with literature.[7]

Cbz-Pro-Ile-OMe (20)

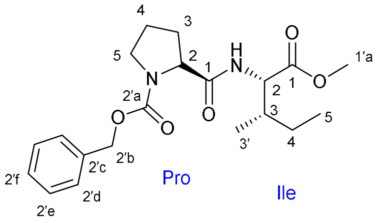

Yield: Method D = 96%.

Purification: flash column chromatography with 40-60 petroleum ether: ethyl acetate = 1:1, R_F = 0.16.

Description: colourless oil.

Characterisation:

$[\alpha]_D$ = -20.4 (*c* 0.80, $CHCl_3$).

^{1}H NMR (500 MHz, DMSO, 373K) δ 7.72 (d, *J* = 7.5 Hz, 1H, Pro-NH), 7.38 – 7.21 (m, 4H, 2 x Pro-C2’dH, 2 x Pro-2’eH and Pro-C2’fH), 5.05 (q, *J* = 12.9 Hz, 2H, Pro-C2’bH_2), 4.37 (dd, *J* = 8.5, 3.1 Hz, 1H, Ile-C2H), 4.25 (dd, *J* = 8.1, 6.2 Hz, 1H, Pro-C2H), 3.62 (s, 3H, Ile-C1’aH_3), 3.49 – 3.37 (m, 2H, Pro-C5H_2), 2.22 – 2.08 (m, 1H, Pro-C3H), 2.00 – 1.68 (m, 4H, Pro-C4H_2, Ile-C3H and Pro-C3H), 1.50 – 1.29 (m, 1H, Ile-C5H), 1.25 – 1.10 (m, 1H, Ile-C5H), 0.82 (t, *J* = 6.8 Hz, 6H, Ile-C4H_3 and Ile-C6H_3). The ^{1}H NMR data was in agreement with literature.[8]

Cbz-Ser-Pro-OMe (23)

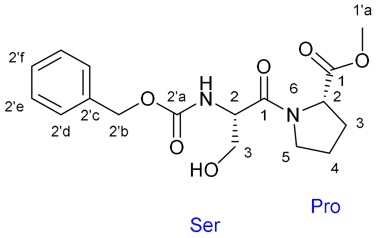

Yield: Method D = 57%.

Purification: flash column chromatography in ethyl acetate, R_F = 0.23.

Description: colourless solid, mp = 112-115 °C, lit = 113.0-115.0 °C[9]).

Characterisation:

$[\alpha]_D$ = -62.7 (*c* 1.66, MeOH), lit = -79.15 (*c* 1.66, MeOH)[9]

^{1}H NMR (600 MHz, $CDCl_3$) δ 7.49 – 7.24 (m, 5H, 2 x Ser-C2’dH, 2 x Ser-C2’eH and Ser-C2’fH), 5.83 (s, 1H, Ser-NH), 5.11 (s, 2H, Ser-C2’bH_2), 4.76 – 4.51 (m, 2H, Pro-C2H and Ser-C2H), 4.02 – 3.63 (m, 7H, Pro-C1’aH_3, Pro-C5H_2 and Ser-C3H_2), 3.25 (s, 1H, Ser-OH), 2.26 (s, 1H, Pro-C3H*H*), 2.01 (s, 3H, Pro-C4H_2 and Pro-C3H*H*). The ^{1}H NMR data was in agreement with literature.[9]

Cbz-Ser(tBu)-Pro-OMe (25)

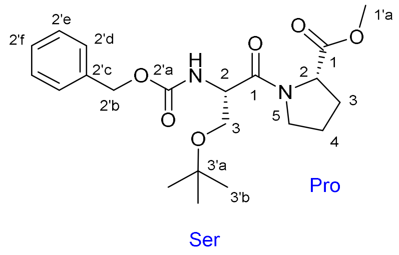

Yield: Method D = 67%.

Purification: flash column chromatography using 40-60 petroleum ether : ethyl acetate = 3:1.

Description: colourless oil.

Characterisation:

[α]$_D$ = -45.1 (*c* 1.00 M, MeOH).

^{1}H NMR (500 MHz, DMSO, 393 K) δ 7.43 – 7.22 (m, 5H, 2 x Ser-2'dH, 2 x Ser-2'eH and Ser-2'fH), 6.56 (s, 1H, Ser-NH), 5.12 – 4.94 (m, 2H, Ser-C2'bH_2), 4.41 (s, 1H, Ser-C2H), 3.62 (s, 3H, Pro-C1'aH_3), 3.51 – 3.41 (m, 3H, Pro-C2H and Ser-C3H_2), 2.87 (s, 2H, Pro-C5H_2), 2.19 – 2.11 (m, 1H, Pro-C3H*H*), 2.03 – 1.77 (m, 3H, Pro-C4H_2 and Pro-C3HH), 1.14 (s, 9H, 3 x Ser-C3'bH_3).

^{13}C NMR (151 MHz, DMSO) *δ* = 171.3 (Pro-C1), 168.4 (Ser-C1), 154.8 (Ser-C2'a), 136.4 (Ser-C2'c), 127.5 (2 x Ser-C2'e), 126.9 (Ser-C2'f), 126.8 (2 x Ser-C2'd), 72.2 (Ser-C3'a), 70.0 (Ser-C2), 65.1 (Ser-C2'b), 58.0 (Ser-C3), 52.7 (Pro-C2), 50.8 (Pro-C1'a), 45.8 (Pro-C5), 26.9 (Pro-C3), 26.5 (Ser-C3'b), 17.9 (Pro-C4).

IR (neat) v/cm^{-1} = 3293 (N-H), 2972 (C-H), 1712 (C=O carbamate), 1640 (C=O amide).

HRMS (ESI) = found 407.2163; $[M + H]^+$ $C_{21}H_{31}N_2O_6$ requires 407.2176; Δ = -3.1 ppm.

Cbz-Thr-Phe-OMe (28)

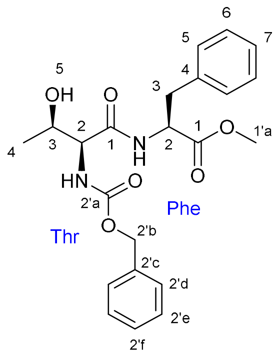

Yield: Method D = 63%.

Purification: flash column chromatography using 40-60 petroleum ether : ethyl acetate = 3:1, R_F = 0.37.

Description : colourless solid, mp = 100-101 °C, lit = 99-101 °C[10].

Characterisation:

$[\alpha]_D$ = -5.4 (*c* 1.00, MeOH), lit = -8.2 (*c* 1.00, MeOH)[10].

^{1}**H NMR** (600 MHz, $CDCl_3$) δ = 7.29-7.21 (m, 5H, Thr-ArH), 7.17-7.13 (m, 2H, 2 x Phe-C6H), 7.11 (m, 1H, Phe-C7H), 7.0 (d, *J* = 7.1 Hz, 2H, 2 x Phe-C5H), 6.94 (d, *J* = 7.7 Hz, 1H, NH), 5.65 (d, *J* = 7.7 Hz, 1H, NH), 5.00 (q, *J* = 12.1 Hz, 2H, Thr-C2'bH_2), 4.76 (dd, *J* = 13.2, 7.2 Hz, 1H, Phe-C2H), 4.19 (d, *J* = 4.9 Hz, 1H, Thr-C3H), 4.05 (m, 1H, Thr-C2H), 3.63 (s, 3H, Phe-C1'aH_3), 3.06 (dd, *J* = 13.9, 5.4 Hz, 2H, Phe-C3H*H* and Thr-OH), 2.92 (dd, *J* = 14.0, 7.1 Hz, 1H, Phe-C3H*H*), 1.04 (d, *J* = 6.5 Hz, 3H, Thr-C4H_3). The ^{1}H NMR data was in agreement with the literature.[9]

Cbz-Thr(Bzl)-Phe-OMe (30)

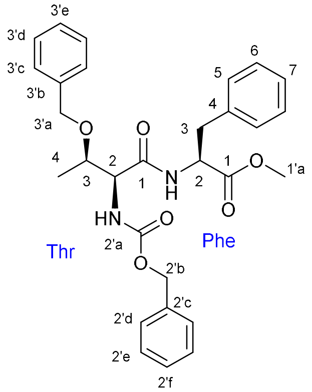

Yield: Method D = 68%.

Purification: flash column chromatography using 40-60 petroleum ether: ethyl acetate= 3:1.

Description: colourless solid, mp = 113-114 °C.

Characterisation:

[α]$_D$ = +7.7 (*c* 1.00 M, MeOH).

^{1}H NMR (600 MHz, $CDCl_3$) *δ* = 7.42-7.27 (m, 10H, 2 x Thr-C2'dH, 2 x Thr-C2'eH, Thr-C2'fH, 2 x Thr-C3'cH, 2 x Thr-C3'dH and Thr-C3'eH), Phe-C7H and Phe-NH), 7.18-7.13 (m, 4H, 2 x Phe-C6H, 7.03-6.95 (m, 2H, 2 x Phe-C5H), 5.76 (d, *J* = 5.8 Hz, 1H, Thr-NH), 5.21-5.01 (m, 2H, Thr-C2'bH_2), 4.81 (dd, *J* = 12.9, 6.6 Hz, 1H, Phe-C2H), 4.63-4.52 (m, 2H, Thr-C3'aH_2), 4.35 (d, *J* = 2.8 Hz, 1H, Thr-C2H), 4.14 (s, 1H, Thr-C3H), 3.68 (s, 3H, Phe-C1'aH_3), 3.04 (dd, *J* = 13.8, 6.0 Hz, 2H, Phe-C3H_2), 1.19 (d, *J* = 6.4 Hz, 3H, Thr-C4H_3).

^{13}C NMR (151 MHz, $CDCl_3$) *δ* = 171.6 (Thr-C1), 169.2 (Phe-C1), 156.3 (Thr-C2'a), 137.9 (Thr-C3'b), 136.2 (Phe-C4), 135.8 (Thr-C2'c), 129.2 (2 x Phe-C5), 128.7 (2 x Thr-C3'd), 128.7 (2 x Phe-C6), 128.5 (2 x Thr-C2'e), 128.3 (2 x Thr-C2'd), 128.1 (2 x Thr-C3'c), 127.9 (Thr-C2'f), 127.9 (Thr-C3'e), 127.2 (Phe-C7), 74.6 (Thr-C3), 71.5 (Thr-C3'a), 67.2 (Thr-C2'b), 57.5 (Thr-C2), 53.6 (Phe-C2), 52.3 (Phe-C1'a), 37.9 (Phe-C3), 14.7 (Thr-C4),

IR (neat) v/cm^{-1} = 3435 (N-H), 3238 (N-H), 2925 (C-H), 1746 (C=O ester), 1716 (C=O carbamate), 1663 (C=O amide).

HRMS (ESI) = found 505.2320; $[M + H]^+$ $C_{29}H_{33}N_2O_6$ requires 505.2333; Δ = -2.5 ppm.

Fmoc-Ala-Ile-OMe (32)

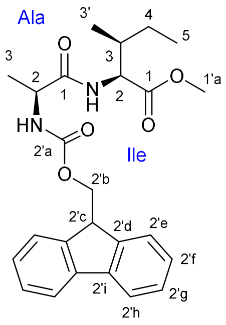

Yield: Method D = 81%.

Purification: flash column chromatography using 40-60 petroleum ether: ethyl acetate= 3:1.

Description: white solid, mp = 100-104 °C.

Characterisation:

[α]$_D$ = -20.0 (*c* 1.00, MeOH).

^{1}H NMR (600 MHz, $CDCl_3$) *δ* = 7.76 (d, *J* = 7.5 Hz, 2H, 2 x Ala-C2’hH), 7.58 (d, *J* = 7.3 Hz, 2H, 2 x Ala-C2’eH), 7.40 (t, *J* = 7.4 Hz, 2H, 2 x Ala-C2’gH), 7.31 (t, *J* = 7.4 Hz, 2H, 2 x Ala-C2’fH), 6.57 (s, *J* = 6.7 Hz, 1H, Ile-NH), 5.46 (d, *J* = 6.0 Hz, 1H, Ala-NH), 4.58 (dd, *J* = 8.6, 4.9 Hz, 1H, Ile-C2H), 4.39 (d, *J* = 6.6 Hz, 2H, Ala-C2’bH_2), 4.32 (s, 1H, Ala-C2H), 4.22 (t, *J* = 7.1 Hz, 1H, Ala-C2’cH), 3.73 (s, 3H, Ile-C1’aH_3), 1.89 (s, 1H, Ile-C3H), 1.49-1.33 (m, 4H, Ile-C4H*H* and Ala-C3H_3), 1.16 (dd, J = 13.9, 7.6 Hz, 1H, Ile-C4H*H*), 0.94-0.84 (m, 6H, Ile-C3’H_3 and Ile-C5H_3).

^{13}C NMR (151 MHz, $CDCl_3$) *δ* = 172.3 (Ala-C1), 172.1 (Ile-C1), 156.1 (Ala-C2’a), 15.6 (Ile-C3’), 143.9 (Ala-C2’d), 141.4 (Ala-C2’i), 127.9 (Ala-C2’f), 127.2 (Ala-C2’g), 125.2 (Ala-C2’e), 120.1 (Ala-C2’h), 67.3 (Ala-C2’b), 56.6 (Ile-C2), 52.3 (Ile-C1’a), 50.6 (Ala-C2), 47.2 (Ala-C2’c), 38.0 (Ile-C3), 25.2 (Ile-C4), 18.7 (Ala-C3), 11.7 (Ile-C5).

IR (neat) v/cm^{-1} = 3299 (N-H), 2975 (C-H), 1733 (C=O ester), 1689 (C=O carbamate), 1653 (C=O amide).

HRMS (ESI) = found 439.2231; $[M + H]^+$ $C_{25}H_{31}N_2O_5$ requires 439.2231; Δ = 0.9 ppm.

Fmoc-Leu-Ile-OMe (34)

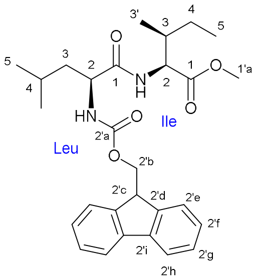

Yield: Method D = 92%.

Purification: flash column chromatography using 40-60 petroleum ether: ethyl acetate= 3:1.

Description: white solid, mp = 61-63 °C.

Characterisation:

[α]D = -23.1 (*c* 1.00, MeOH).

^{1}H NMR (600 MHz, $CDCl_3$) *δ* = 7.76 (d, *J* = 7.6 Hz, 2H, 2 x Leu-C2’hH), 7.58 (d, *J* = 7.4 Hz, 2H, 2 x Leu-C2’eH), 7.40 (t, *J* = 7.5 Hz, 2H, 2 x Leu-C2’gH), 7.31 (t, *J* = 7.5 Hz, 2H, 2 x Leu-C2’fH), 6.44 (d, *J* = 7.9 Hz, 1H, Ile-NH), 5.22 (d, *J* = 7.9 Hz, 1H, Leu-NH), 4.58 (dd, *J* = 8.6, 4.9 Hz, 1H, Ile-C2H), 4.40 (p, *J* = 10.6 Hz, 2H, Leu-C2’bH_2), 4.22 (t, *J* = 7.0 Hz, 2H, Leu-C2H and Leu-C2’cH), 3.73 (s, 3H, Ile-C1’aH_3), 1.89 (s, 1H, Ile-C3H), 1.71-1.63 (m, 2H, Leu-C3H_2), 1.55 (m, 1H, Leu-C4H), 1.41 (m, 1H, Ile-C4H*H*), 1.16 (m, 1H, Ile-C4H*H*), 0.95 (s, 6H, Leu-C5H_3 and Leu-C6H_3), 0.94-0.87 (m, 6H, Ile-C3’H_3 and Ile-C5H_3).

^{13}C NMR (151 MHz, $CDCl_3$) *δ* = 172.2 (Ile-C1), 172.0 (Leu-C1), 156.3 (Leu-C2’a), 144.0 (Leu-C2’d), 143.9 (Leu-C’2’d), 141.4 (2 x Leu-C2’i), 127.9 (2 x Leu-C2’g), 127.2 (2 x Leu-C2’f), 125.2 (2 x Leu-C2’e), 120.1 (2 x Leu-C2’h), 67.2 (Leu-C2’b), 56.6 (Ile-C2), 53.6 (Leu-C2), 52.3 (Ile-C1’a), 47.3 (Leu-C2’c), 41.5 (Leu-C4), 38.0 (Ile-C3), 25.2 (Ile-C4), 24.8 (Leu-C3), 22.2 and 23.0 (Leu-C5 and Leu-C4), 15.6 (Ile-C5), 11.7 (Ile-C3’).

IR (neat) v/cm^{-1} = 3307 (N-H), 2957 (C-H), 1743 (C=O ester), 1697 (C=O carbamate), 1656 (C=O amide).

HRMS (ESI) = found 481.2703; $[M + H]^+$ $C_{28}H_{37}N_2O_5$ requires 481.2697; Δ = 1.2 ppm.

Fmoc-Tyr(OtBu)-Ala-OBn (38)

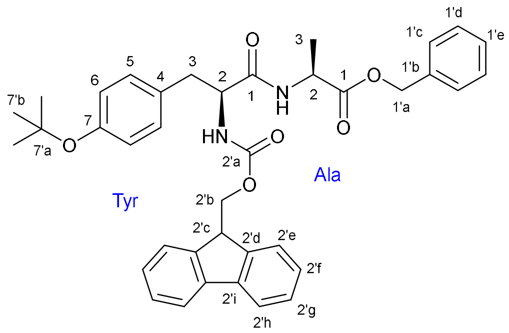

Yield: Method D = 81%.

Purification: flash column chromatography using 40-60 petroleum ether: ethyl acetate= 3:1.

Description: white solid, mp = 132-133 °C.

Characterisation:

[α]$_D$ = -7.9 (*c* 1.00, MeOH).

^{1}H NMR (600 MHz, $CDCl_3$) *δ* = 7.76 (dd, *J* = 7.4 Hz, 2H, 2 x Tyr-C2'hH), 7.56 (t, *J* = 7.6 Hz, 2H, 2 x Tyr-C2'gH), 7.44-7.28 (m, 9H, 2 x Tyr-C2'fH, 2 x Tyr-C2'eH, 2 x Ala-C1'cH, 2 x Ala-C1'dH and Ala-1'eH), 7.08 (s, 2H, 2 x Tyr-C5H), 6.89 (d, *J* = 8.0 Hz, 2H, 2 x 2 x Tyr-C6H), 6.44 (s, 1H, Ala-NH), 5.48 (d, *J* = 5.1 Hz, 1H, Tyr-NH), 5.15 (s, 2H, Ala-C1'aH_2), 4.55 (m, 1H, Ala-C2H), 4.45-4.39 (m, 2H, and Tyr-C2'bH_2), 4.32 (s, 1H, Tyr-C2H), 4.18 (t, *J* = 6.9 Hz, 1H, Tyr-C2'cH), 3.07-2.99 (m, 2H, Tyr-C3H_2), 1.35-1.31 (m, 12H, 3 x Tyr-C7'bH_3 and Ala-C3H_3). The ^{1}H NMR data was in agreement with the literature.[11]

Boc-D-Ala-Val-OBn (16)

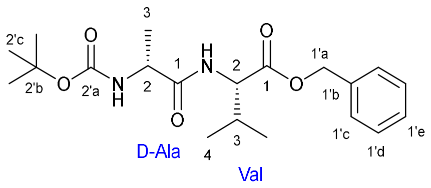

Yield: Method D = 96%.

Purification: flash column chromatography using 40-60 petroleum ether: ethyl acetate= 3:1.

Description: colourless solid, mp = 53-55 °C.

Characterisation:

[α]$_D$ = +10.9 (*c* 1.44, MeOH), lit = +4.4 (*c* 1.44, MeOH)[12]

^{1}H NMR (600 MHz, $CDCl_3$) *δ* = 7.44-7.28 (m, 5H, 2 x Val-C1'cH, 2 x Val-C1'dH and Val-C1'eH), 6.78 (s, 1H, Val-NH), 5.19 (dd, *J* = 12.2 Hz, 1H, Val-C1'aH*H*), 5.11 (dd, *J* = 12.2 Hz, 1H, Val-C1'aH*H*), 5.00 (s, 1H, Ala-NH), 4.57 (dd, *J* = 8.8, 4.7 Hz, 1H, Val-C2H), 4.22 (s, 1H, Ala-C2H), 2.19 (m, 1H, Val-C3H), 1.44 (s, 9H, 3 x Ala-C2'cH_3), 1.36 (d, *J* = 7.1 Hz, 3H, Ala-C3H_3), 0.92 (d, *J* = 6.9 Hz, 3H, Val-C4H_3), 0.85 (d, *J* = 6.9 Hz, 3H, Val-C4H_3). The ^{1}H NMR data was in agreement with that reported in the literature.[12]

Boc-Ala-Val-OBn (17)

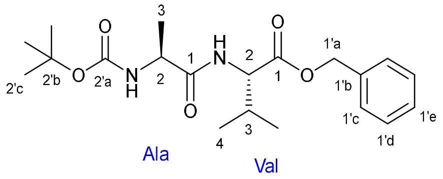

Yield: Method D = 98%.

Purification: flash column chromatography using 40-60 petroleum ether: ethyl acetate= 3:1.

Description: colourless oil.

Characterisation:

[α]$_D$ = -5.3 (*c* 0.67, $CHCl_3$), lit = α]$_D$ = -36.6 (*c* 0.67, $CHCl_3$).[12]

^{1}H NMR (600 MHz, $CDCl_3$) *δ* = 7.43-7.20 (m, 5H, 2 x Val-C1'cH, 2 x Val-C1'dH and Val-C1'eH), 6.82 (s, 1H, Val-NH), 5.35-5.00 (m, 3H, Ala-NH and Val-C1'aH_2), 4.56 (dd, *J* = 8.7, 4.8 Hz, 1H, Val-C2H), 4.21 (s, 1H, Ala-C2H), 2.17 (dd, *J* = 12.2, 6.5 Hz, 1H, Val-C3H), 1.42 (s, 9H, 3 x Ala-C2'cH_3), 1.32 (d, *J* = 6.9 Hz, 3H, Ala-C3H_3), 0.89 (d, *J* = 6.9 Hz, 3H, Val-C4H_3), 0.84 (d, *J* = 6.9 Hz, 3H, Val-C4H_3). The ^{1}H NMR data was in agreement with that reported in the literature.[12]

Boc-Gly-Val-OBn (43)

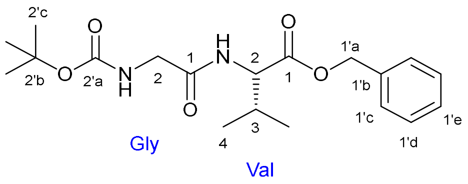

Yield: Method D = 98%, Method H = 93%.

Purification: flash column chromatography using 40-60 petroleum ether: ethyl acetate= 7:3, R_F = 0.31.

Description: colourless oil.

Characterisation:

[α]$_D$ = +11 (*c* 0.72, $CHCl_3$), lit = **[α]$_D$** = +11 (*c* 0.72, $CHCl_3$)[13].

^{1}H NMR (600 MHz, $CDCl_3$) *δ* = 7.27-7.33 (m, 5H, 2 x Val-C1'cH, 2 x Val-C1'dH and Val-C1'eH), 7.01 (s, 1H, Gly-NH), 5.61 (s, 1H, Val-NH), 5.15 (d, *J* = 12.2 Hz, 1H, Val-C1'aH*H*), 5.08 (d, *J* = 12.3 Hz, 1H, Val-C1'aHH), 4.57 (dd, *J* = 8.4, 4.8 Hz, 1H, Val-C2H), 3.80 (s, 2H, Gly-C2H_2), 2.15 (dd, *J* = 12.0, 6.8 Hz, 1H, Val-C3H), 1.41 (s, 9H, 3 x Gly-C2'cH_3), 0.88 (d, *J* = 6.9 Hz, 3H, Val-C4H_3), 0.82 (d, *J* = 7.0 Hz, 3H, Val-C4H_3). The ^{1}H NMR data was in agreement with that reported in the literature.[13]

Boc-Pro-Val-OBn (45)

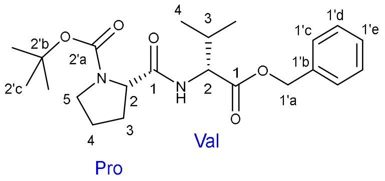

Yield: Method D = 84%, Method H = 84%.

Purification: flash column chromatography using 40-60 petroleum ether: ethyl acetate= 3:1.

Description: colourless oil.

Characterisation:

[α]$_D$ = -63.4 (*c* 1.00, MeOH).

^{1}H NMR (600 MHz, $CDCl_3$) *δ* = 7.53 (s, 1H, Val-NH), 7.27-7.34 (m, 5H, 2 x Val-C1'cH, 2 x Val-C1'dH and Val-C1'eH), 5.12-5.18 (m, 2H, Val-C1'aH_2), 5.51-4.57 (m, 1H, Pro-C2H), 4.34-4.24 (m, 1H, Val-C2H), 3.47-3.31 (m, 2H, Pro-C5H_2), 2.16 (s, 2H, Pro-C3H_2), 1.92-1.86 (m, 3H, Val-C3H and Pro-C4H_2), 1.45 (s, 9H, 3 x Pro-C2'cH_3), 0.87 (d, *J* = 6.9 Hz, 3H, Val-C4H_3), 0.83 (d, *J* = 5.7 Hz, 3H, Val-C4H_3). The ^{1}H NMR was in agreement with that published in the literature.[14]

Boc-Trp(H)-Ala-OBn (47)

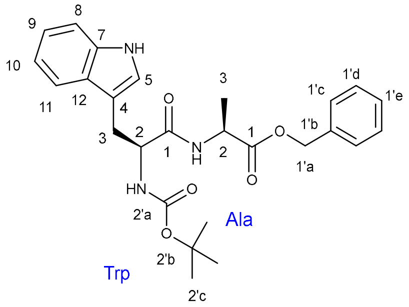

Yield: Method D = 85%, Method H = 85%.

Purification: flash column chromatography using 40-60 petroleum ether: ethyl acetate = 7:3, R_F = 0.30.

Characterisation: colourless solid, mp = 104-106 °C.

[α]$_D$ = -21.4 (*c* 1.00, MeOH).

^{1}H NMR (600 MHz, $CDCl_3$) *δ* = 8.14 (bs, 1H, Trp-N6H), 7.65 (d, *J* = 7.7 Hz, 1H, Trp-C11H), 7.41-7.28 (m, 6H, Trp-C8H, 2 x Ala-C1'cH, 2 x Ala-C1'dH, Ala-C1'eH), 7.18 (t, *J* = 7.4 Hz, 1H, Trp-C9H), 7.11 (t, *J* = 7.4 Hz, 1H, Trp-C10H), 7.02 (s, 1H, Trp-C5H), 6.36 (d, *J* = 7.1 Hz, 1H, Ala-NH), 5.21 (s, 1H, Trp-NH), 5.08 (s, 2H, Ala-C1'aH_2), 4.52-4.45 (m, 2H, Trp-C2H and Ala-C2H), 3.21 (m, 2H, Trp-C3H_2), 1.43 (s, 9H, 3 x Trp-C2'cH_3), 1.27 (d, *J* = 6.9 Hz, 3H, Ala-C3H_3).

^{13}C NMR (151 MHz, $CDCl_3$) *δ* = 172.2 (Trp-C1), 171.2 (Ala-C1), 155.4 (Trp-C1'a), 136.2 (Trp-C7), 135.4 (Ala-C1'b), 128.6 (Ala-C1'd), 128.5 (Ala-C1'e), 128.1 (Ala-C1'c), 123.3 (Trp-C5), 122.2 (Trp-C9), 120.0 (Trp-C10), 118.9 (Trp-C11), 111.2 (Trp-C8), 110.5 (Trp-C4), 80.0 (Ala-C2'b), 67.0 (Ala-C1'a), 55.1 (Trp-C2), 48.2 (Ala-C2), 28.3 (Trp-C1'c), 18.3 (Ala-C3).

IR (neat) v/cm^{-1} = 3418 (N-H), 3331 (N-H), 2964 (C-H), 1739 (C=O ester), 1668 (C=O carbamate), 1652 (C=O amide).

HRMS (ESI) = found 466.2332; $[M + H]^+$ $C_{26}H_{32}N_3O_5$ requires 466.2336; Δ = -0.9 ppm.

Boc-Trp(H)-Ala-Gly-Val-OBn (50)

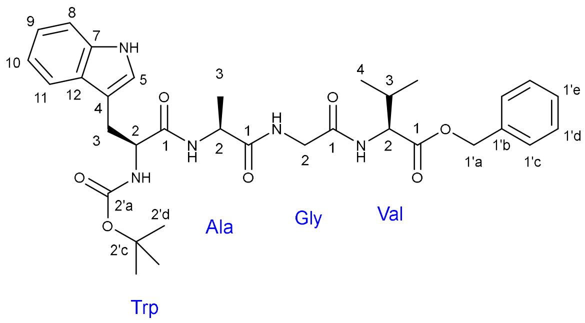

Yield: Method D = 42%.

Purification: flash column chromatography using 8% methanol in dichloromethane.

Description: yellow solid, mp = 97-99 °C.

Characterisation:

[α]$_D$ = -12.5 (*c* 1.00, MeOH).

^{1}H NMR (500 MHz, DMSO, T = 100 °C) *δ* = 10.5 (s, 1H, Trp-N6H), 7.77 (s, 1H, Gly-NH), 7.73-7.63 (m, 2H, Val-NH and Ala-NH), 7.58 (d, *J* = 7.8 Hz, 1H, Trp-C11H), 7.43-7.26 (m, 6H, Val-ArH and Trp-C8H), 7.12 (s, 1H, Trp-C5H), 7.06 (t, *J* = 7.5 Hz, 1H, Trp-C9H), 6.98 (t, *J* = 7.4 Hz, 1H, Trp-C10H), 6.28 (s, 1H, Trp-NH), 5.16 (s, 2H, Val-C1'aH_2), 4.42-4.22 (m, 3H, Ala-C2H, Trp-C2H and Val-C2H), 3.89-3.71 (m, 2H, Gly-C2H_2), 3.18 (dd, *J* = 14.7, 5.0 Hz, 1H, Trp-C3H), 2.99 (m, 1H, Trp-C3H), 2.11 (dd, *J* = 13.5, 6.7 Hz, 1H, Val-C3H), 1.33 (s, 9H, 3 x Trp-C2'cH_3), 1.26 (d, *J* = 7.0 Hz, 3H, Ala-C3H_3), 0.90 (d, *J* = 6.8 Hz, 6H, 2 x Val-C4H_3).

^{13}C NMR (151 MHz, DMSO, T = 100 °C) *δ* = 171.9 (Val-C1), 171.1 (Ala-C1), 170.5 (Trp-C1), 168.4 (Gly-C1), 154.6 (Trp-C2'a), 135.9 (Trp-C7), 135.5 (Val-C1'b), 127.8 (2 x Val-C1'd), 127.5 (Val-C1'e), 127.4 (2 x Val-C1'c), 127.2 (Trp-C12), 123.1 (Trp-C5), 120.3 (Trp-C9), 117.9 (Trp-C11), 117.7 (Trp-C10), 110.7 (Trp-C8), 109.8 (Trp-C4), 65.5 (Val-C1'a), 57.2 (Val-C2), 55.1 (Trp-C2), 48.1 (Ala-C2), 41.8 (Gly-C2), 29.6 (Val-C3), 27.7 (3 x Trp-C2'd), 27.3 (Trp-C3), 18.3 (Ala-C3), 17.5 (2 x Val-C4).

IR (neat) v/cm^{-1} = 3311 (N-H), 2976 (C-H), 1733 (C=O ester), 1689 (C=O carbamate), 1653 (C=O amide).

HRMS (ESI) = found 622.3239; $[M + H]^+$ $C_{33}H_{44}N_5O_7$ requires 622.3239; Δ = -0.6 ppm.

Boc-Pro-Val-Trp(H)-Ala-OBn (53)

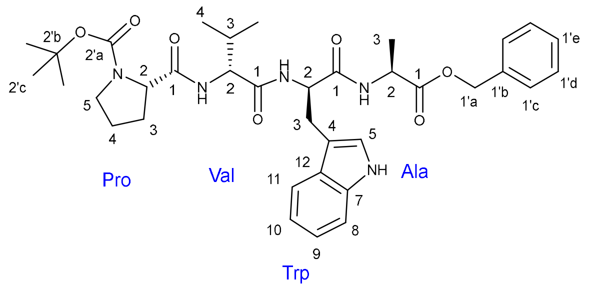

Yield: Method D = 42%.

Purification: flash column chromatography using 5% methanol in dichloromethane.

Description: Pink pale solid, mp = 94-96 °C.

Characterisation:

[α]$_D$ = -61.0 (*c* 1.00, MeOH).

^{1}**H NMR** (600 MHz, DMSO, T = 120 °C) δ = 10.41 (s, 1H, Trp-N6H), 7.75 (s, 1H, Trp-C11H), 7.52 (d, *J* = 7.4 Hz, 2H, 2 x Ala-C1'cH), 7.34-7.27 (m, 6H, Trp-C8H, Trp-C11H, 2 x Ala-C1'dH, Ala-C1'eH and NH), 7.08 (s, 1H, Trp-C5H), 7.04 (t, *J* = 7.3 Hz, 1H, Trp-C9H), 6.95 (t, *J* = 7.3 Hz, 1H, Trp-C10H), 5.11 (s, 2H, Ala-C1'aH_2), 4.64 (dd, *J* = 7.4, 13.6 Hz, 1H, Trp-C2H), 4.38 (m, 1H, Pro-C2H), 4.19 (m, 1H, Ala-C2H), 4.12 (m, 1H, Val-C2H), 3.33 (t, *J* = 6.6 Hz, 2H, Pro-C5H_2), 3.15 (dd, *J* = 5.6, 14.8 Hz, 1H, Trp-C3H), 3.01 (dd, *J* = 7.6, 14.8 Hz, 1H, Trp-C3H), 1.98 (m, 2H, Val-C3H and Pro-C3H), 1.77 (m, 3H, Pro-C4H_2 and Pro-C3H), 1.37 (s, 9H, 3 x Pro-C2'cH_3), 1.27 (d, *J* = 7.0 Hz, 3H, Ala-C3H_3), 0.83 (s, 6H, 2 x Val-C4H_3).

^{13}**C NMR** (151 MHz, DMSO, T = 120 °C) δ = 171.5 (Pro-C1), 171.2, 170.4 and 169.9 (Trp-C1, Val-C1 and Ala-C1), 153.3 (Pro-C2'a), 135.8 (Trp-C7), 135.5 (Ala-C1'b), 127.6 (2 x Ala-C1'c), 127.2 (Ala-C1'e), 127.0 (Trp-C12), 126.9 (2 x Ala-C1'd), 122.8 (Trp-C5), 120.2 (Trp-C9), 117.6 (Trp-C11), 117.6 (Trp-C10), 110.6 (Trp-C8), 109.4 (Trp-C4), 78.3 (Pro-C2'b), 65.3 (Ala-C1'a), 59.1 (Pro-C2), 57.5 (Val-C2), 52.6 (Trp-C2), 47.3 (Ala-C2), 46.0 (Pro-C5), 29.8 (Val-C3), 29.3 (Trp-C3), 27.5 (3 x Pro-C2'c), 27.1 (Pro-C3), 22.8 (Pro-C4), 18.4 (Ala-C3), 17.2 (Val-C4), 16.3 (Val-C4).

IR (neat) v/cm^{-1} = 3298 (N-H), 2976 (C-H), 1733 (C=O ester), 1689 (C=O carbamate), 1653 (C=O amide).

HRMS (ESI) = found 662.3538; [M + H]$^+$ $C_{36}H_{48}N_5O_7$ requires 661.3548 ; Δ = 1.5 ppm.

Boc-Gly-Val-Pro-Val-OBn (56)

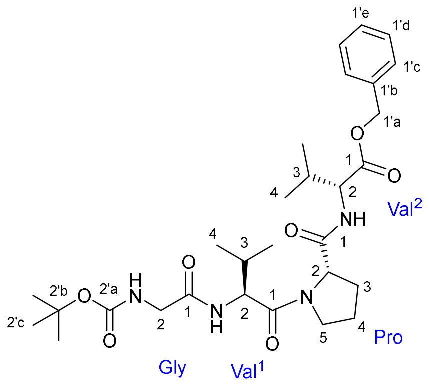

Yield: Method D = 57%, Method G = see Table 4, Method H = 57%, Method I = 72%, Method J = 51%.

Purification: flash column chromatography using 40-60 petroleum ether: ethyl acetate = 1:4, R_F = 0.34.

Description: amorphous yellow solid.

Characterisation:

[α]D = -41.6 (*c* 1.00, MeOH).

^{1}H NMR (500 MHz, DMSO, T = 120 °C) δ = 7.66 (s, 1H, NH), 7.48-7.20 (m, 5H, Val-ArH), 6.50 (s, 1H, NH), 5.13 (q, *J* = 12.5 Hz, 2H, Val-C1'aH_2), 4.53-4.51 (m, 1H, Pro-C2H), 4.44-4.41 (m, 1H, Val-C2H), 4.26-4.23 (m, 1H, Val-C2H), 3.74-3.54 (m, 4H, Pro-C5H_2, Gly-C2H_2), 2.12-1.83 (m, 6H, 2 x Val-C3H, Pro-C4H_2 and Pro-C3H_2), 1.40 (s, 9H, 3 x Gly-C2'cH_3), 0.93-0.86 (m, 12H, 4 x Val-C4H_3).

^{13}C NMR (151 MHz, DMSO, T = 120°C) δ = 171.6 (Val-C1), 171.1 (Val-C1), 170.1 (Pro-C1), 169.1 (Gly-C1), 155.7 (Gly-C2'a), 136.1 (Val-C1'b), 128.4 (2 x Val-C1'd), 128.0 (Val-C1'e), 127.9 (2 x Val-C1'c), 78.5 (Gly-C2'b), 66.0 (Val-C1'a), 59.3 (Pro-C2), 57.8 (Val-C2), 55.4 (Val-C2), 47.2 (Gly-C2), 44.1 (Pro-C5), 30.7 (2 x Val-C3), 30.1 (Pro-C3), 28.3 (Gly-C2'c), 24.4 (Pro-C4), 19.2 (Val-C4), 18.8 (Val-C4), 18.2 (Val-C4), 17.8 (Val-C4).

IR (neat) v/cm^{-1} = 3308 (N-H), 2967 (C-H), 1719 (C=O ester), 1671 (C=O carbamate), 1626 (C=O amide).

HRMS (ESI) = found 583.3135; $[M + Na]^+$ $C_{29}H_{44}N_4O_7Na$ requires 583.3108; Δ = 4.6 ppm.

Boc-Trp(H)-Ala-Gly-Val-Pro-Val-OBn (5)

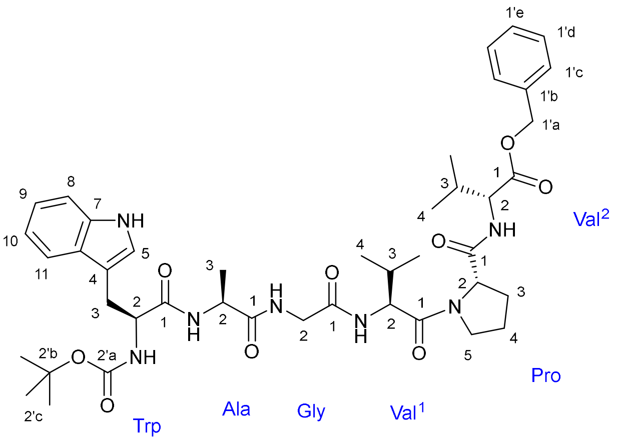

Yield: Method D = 21%, Method H = 87%.

Purification: flash column chromatography using 5% methanol in dichloromethane.

Description: pink pale solid, mp =145-147 °C.

Characterisation:

$[\alpha]_D$ = -35.3 (*c* 1.00, MeOH).

^{1}H NMR (500 MHz, DMSO, T = 120°C) δ = 10.44 (s, 1H, Trp-N6H), 7.69-7.57 (m, 3H, Trp-C8H, Trp-C11H and NH), 7.37-7.33 (m, 5H, Val-ArH), 7.12 (s, 1H, Trp-C5H), 7.06 (t, *J* = 7.5 Hz, 1H, Trp-C9H), 6.97 (t, *J* = 7.4 Hz, 1H, Trp-C10H), 6.21 (s, 1H, NH), 5.15 (q, *J* = 12.5 Hz, 2H, Val-C1'aH_2), 4.52 (m, 1H, Pro-C2H), 4.46-4.40 (m, 1H, Val-C2H), 4.38-4.33 (m, 1H, Ala-C2H), 4.31-4.25 (m, 2H, Trp-C2H and Val-C2H), 3.80-3.54 (m, 5H, Pro-C5H_2, Gly-C2H_2 and NH), 3.18 (dd, *J* = 14.6, 5.0 Hz, 1H, Trp-C3H*H*), 3.00 (dd, *J* = 14.7, 8.3 Hz, 1H, Trp-C3H*H*), 2.12-1.80 (m, 6H, 2 x Val-C3H, Pro-C4H_2 and Pro-C3H_2), 1.34 (s, 9H, 3 x Trp-C2'cH_3) 1.29-1.26 (m, 3H, Ala-C3H_3), 0.95-0.89 (m, 12H, 4 x Val-C4H_3.

^{13}C NMR (151 MHz, DMSO, T = 120°C) δ = 172.8 (Val-C1), 171.9 (Trp-C1), 171.4 (Ala-C1), 170.5 (Val-C1), 169.1 (Pro-C1), 168.8 (Gly-C1), 155.5 (Trp-C2'a), 136.9 (Trp-C7), 136.5 (Val-C1'b), 128.7 (2 x Val-C1'd), 128.4 (Val-C1'e), 128.3 (2 x Val-C1'c), 128.2 (Trp-C12), 124.0 (Trp-C5), 121.2 (Trp-C9), 118.8 (Trp-C11), 118.7 (Trp-C10), 111.7 (Trp-C8), 110.8 (Trp-C4), 78.9 (Trp-C2'b), 70.7 (Val-C2), 66.4 (Val-C1'a), 59.7 (Pro-C2), 58.2 (Trp-C2), 56.0 (Val-C2), 49.0 (Ala-C2), 47.6 (Gly-C2), 42.9 (Pro-C5), 30.9 (Val-C3), 30.5 (Val-C3), 28.9 (Pro-C3), 28.6 (Trp-C2'c), 28.3 (Trp-C3), 24.8 (Pro-C4), 19.5 (Val-C4), 19.2 (Val-C4), 18.6 (Val-C4), 18.5 (Val-C4), 18.4 (Ala-C3).

IR (neat) v/cm^{-1} = 3293 (N-H), 2967 (C-H), 1624 (broad, C=O peaks combined).

HRMS (ESI) = found 818.4420; $[M + H]^+$ $C_{43}H_{60}N_7O_9$ requires 818.4453; Δ = 4.03 ppm.

NMR Spectra

Boc-Trp(CHO)-Ala-OBn (11)

Boc-Trp(CHO)-Ala-OBn, ^{1}H NMR, $CDCl_3$, 600 MHz

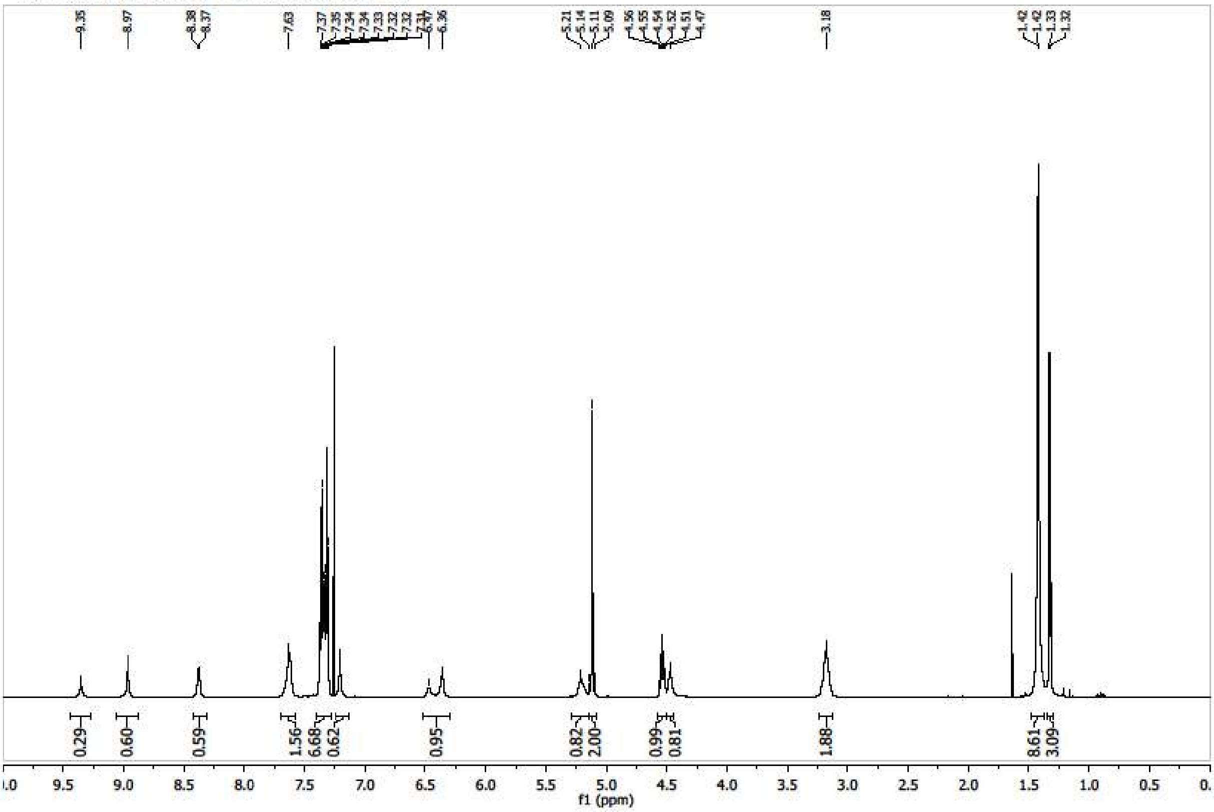

Boc-Trp(CHO)-Ala-OBn, ^{13}C NMR, $CDCl_3$, 151 MHz

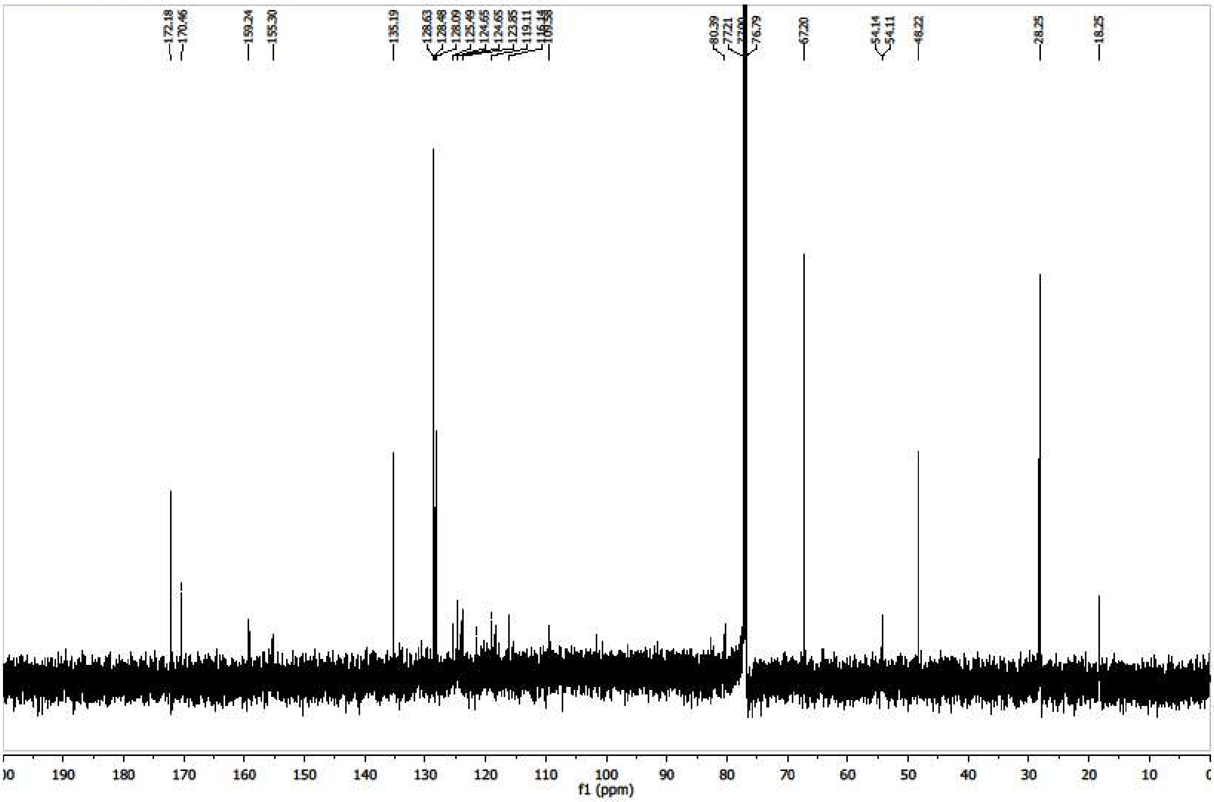

Boc-Trp(CHO)-Ala-OBn, COSY

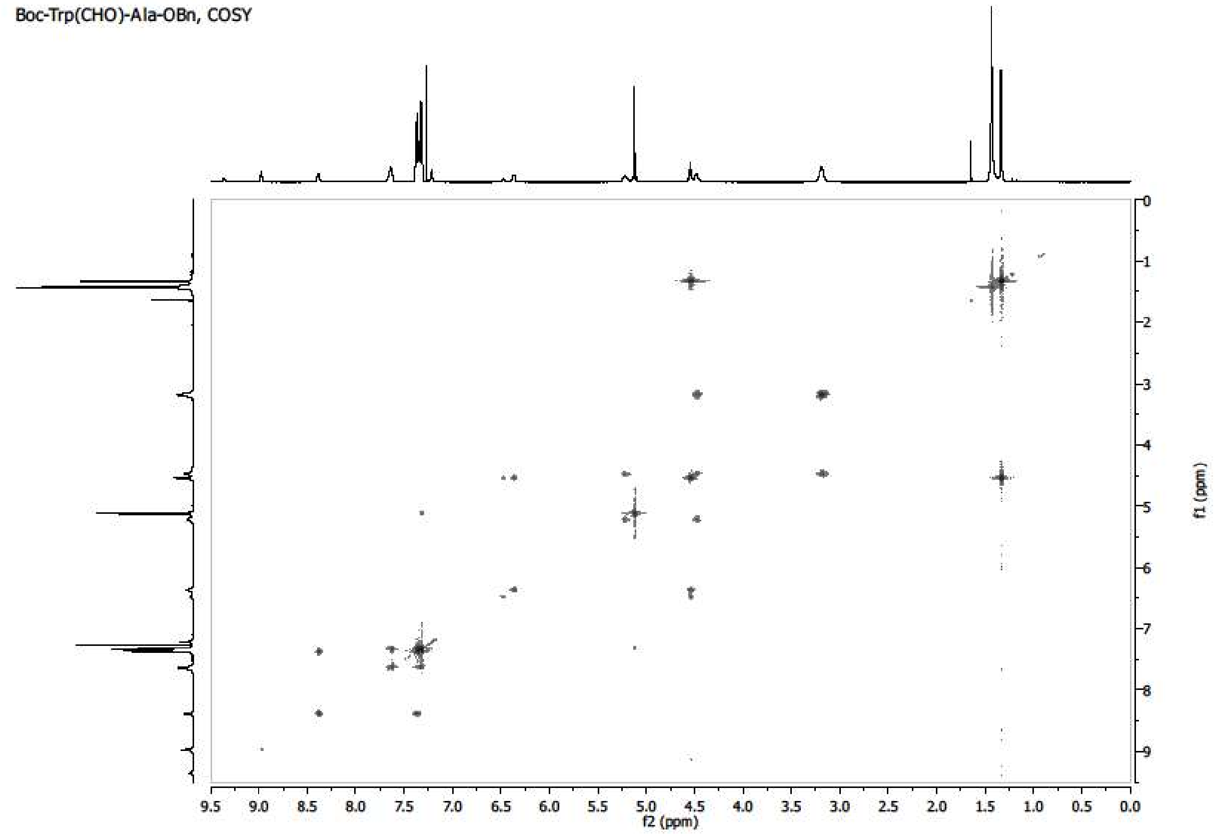

Boc-Trp(CHO)-Ala-OBn, HSQC

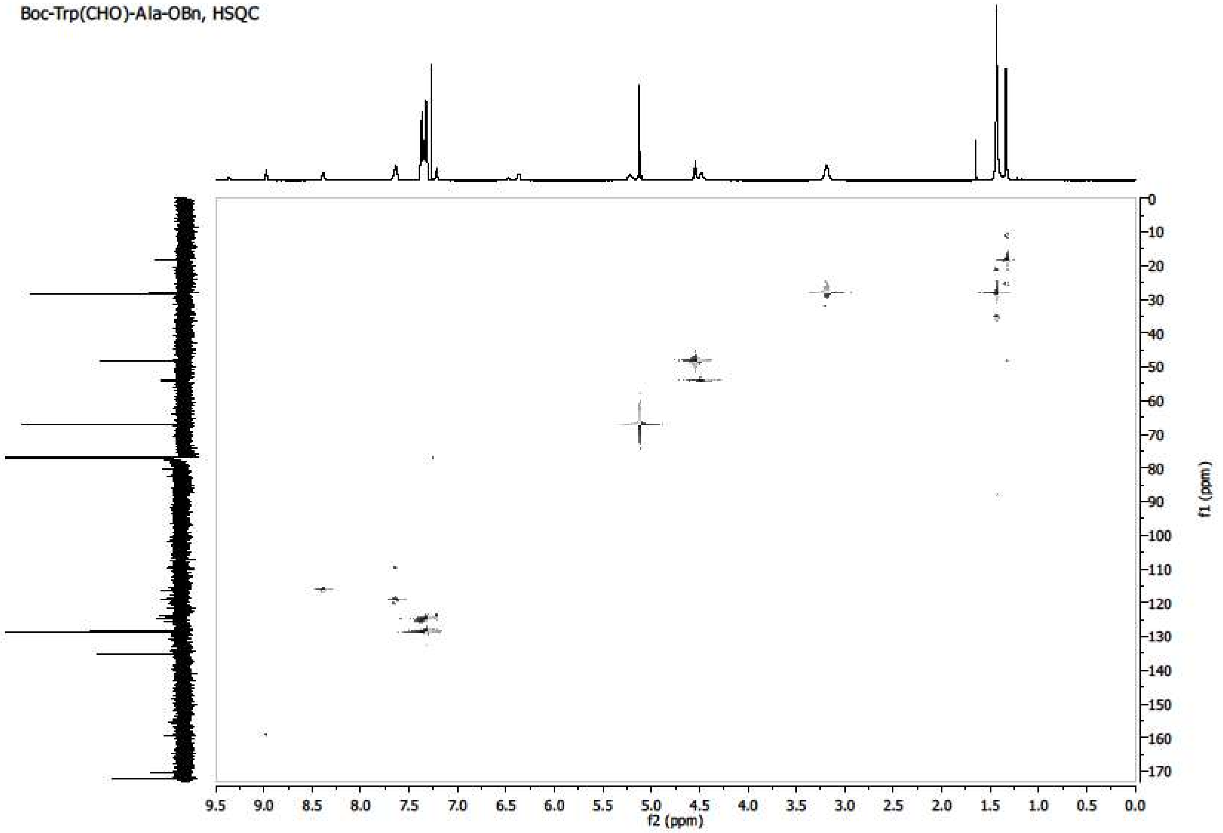

Boc-Trp(CHO)-Ala-OBn, HMBC

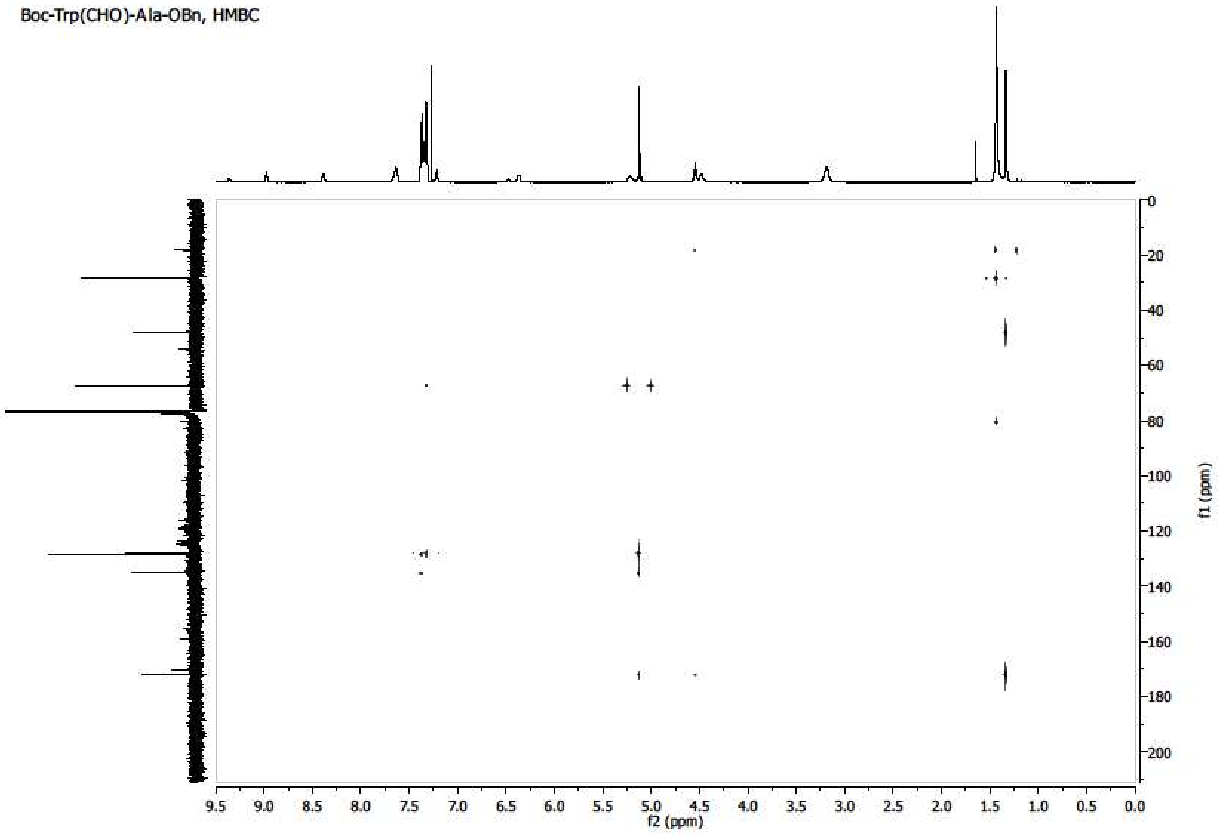

Cbz-Phe-Val-OMe (14)

Cbz-Phe-Val-OMe, ^{1}H NMR, $CDCl_3$, 600 MHz

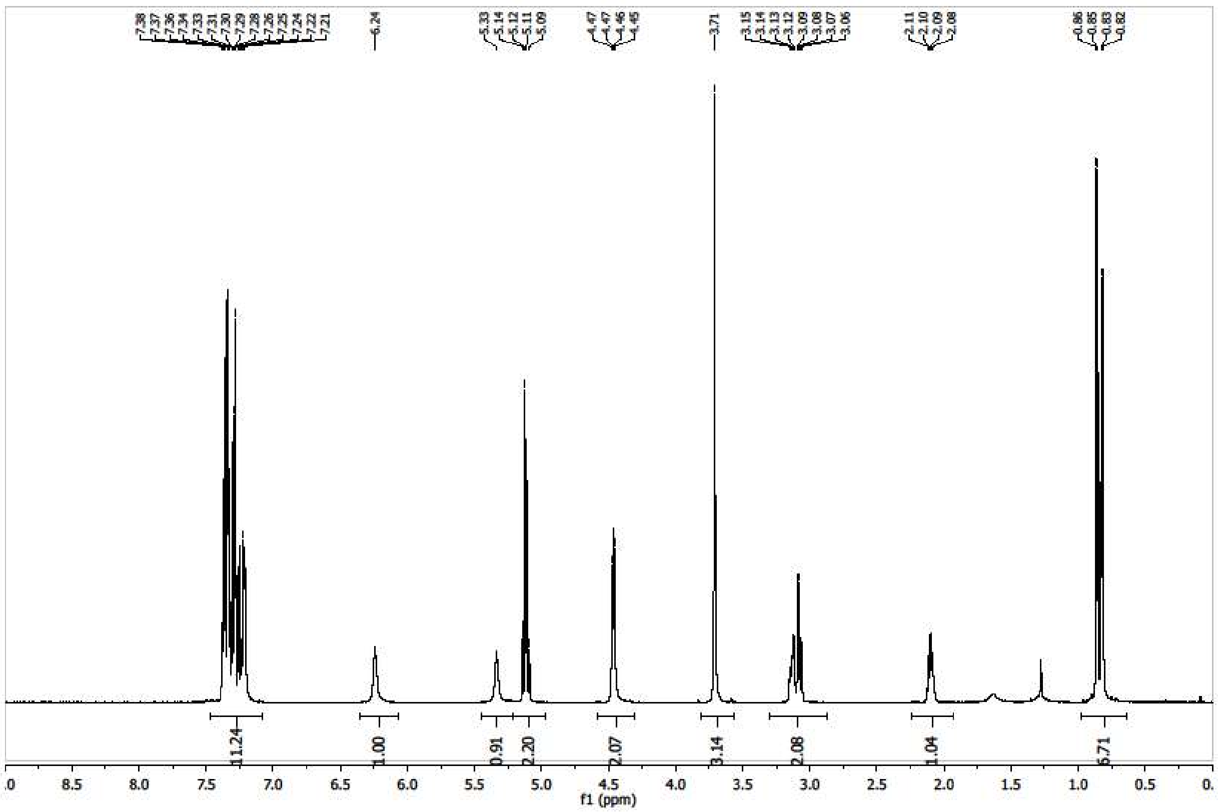

Cbz-Pro-Ile-OMe (20)

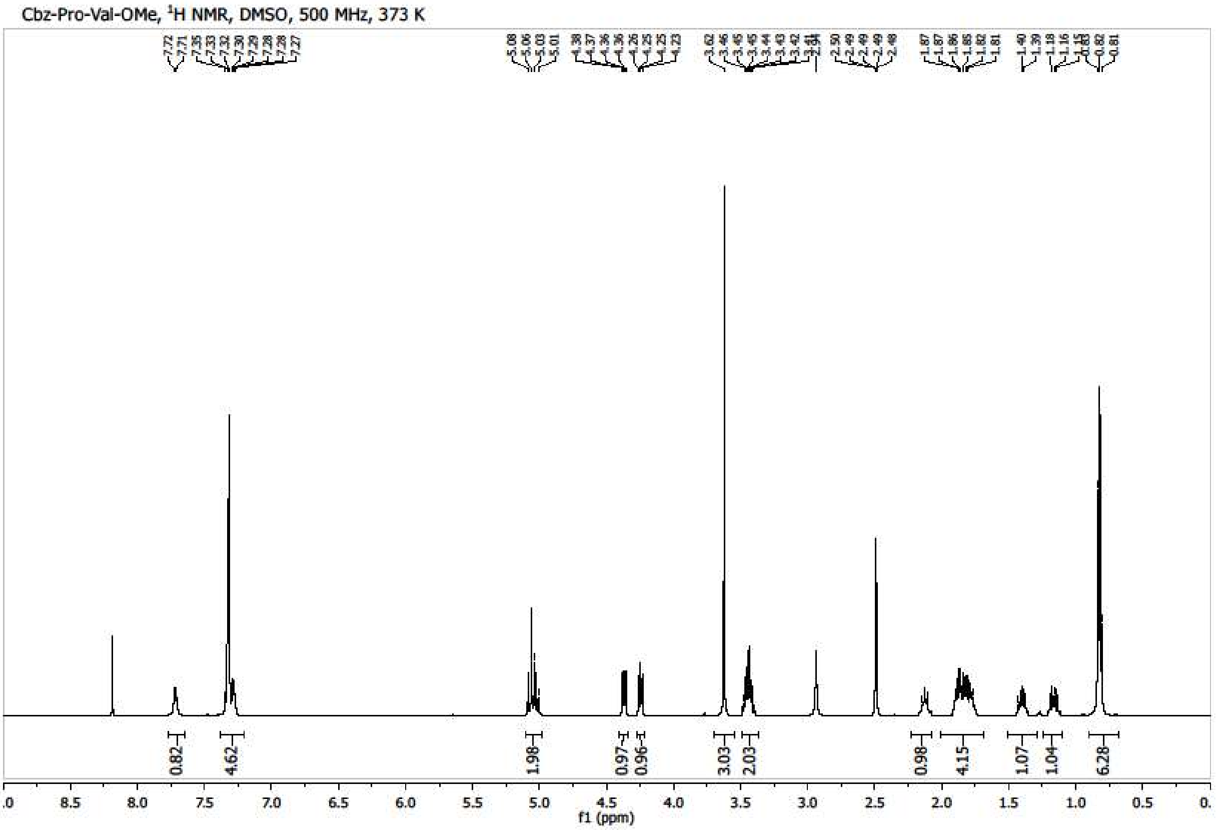

Cbz-Ser-Pro-OMe (23)

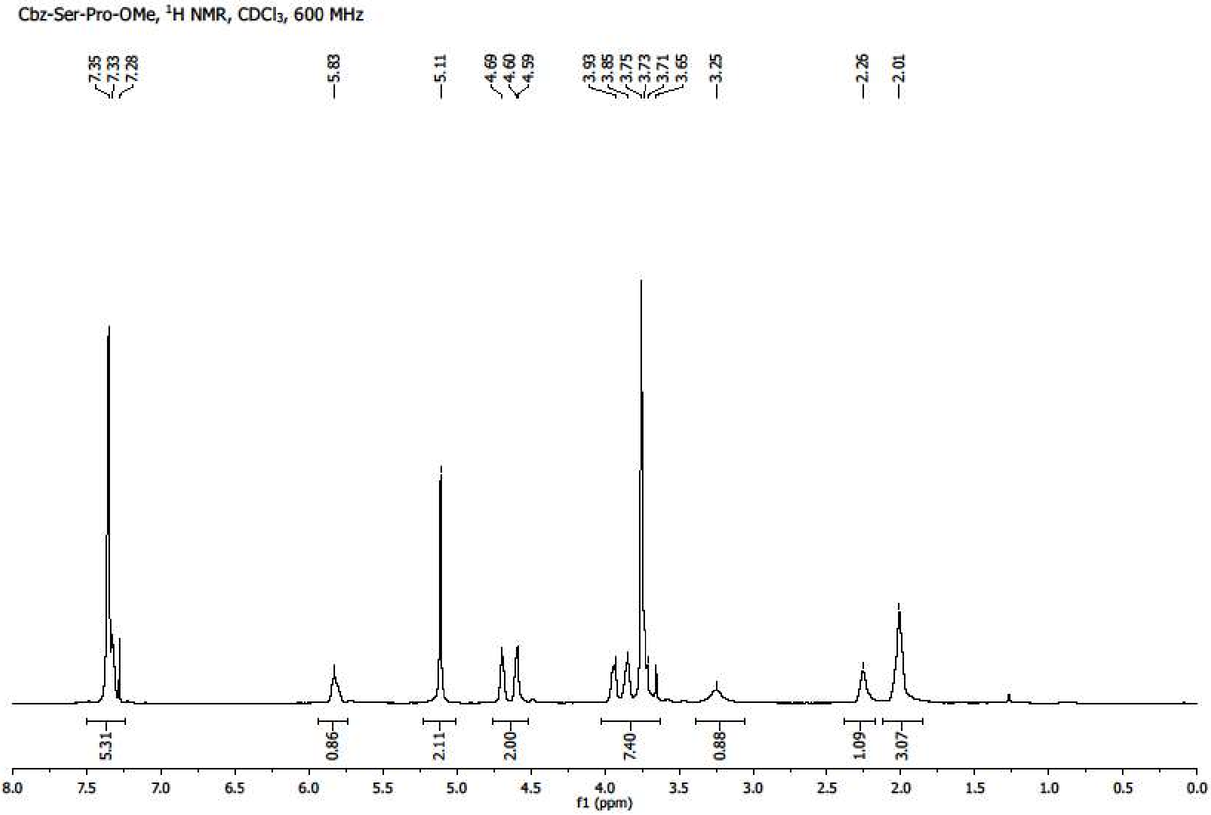

Cbz-Ser(tBu)-Pro-OMe (25)

Cbz-Ser(tBu)-Pro-OMe, ^{1}H NMR, DMSO, 500 MHz, 393 K

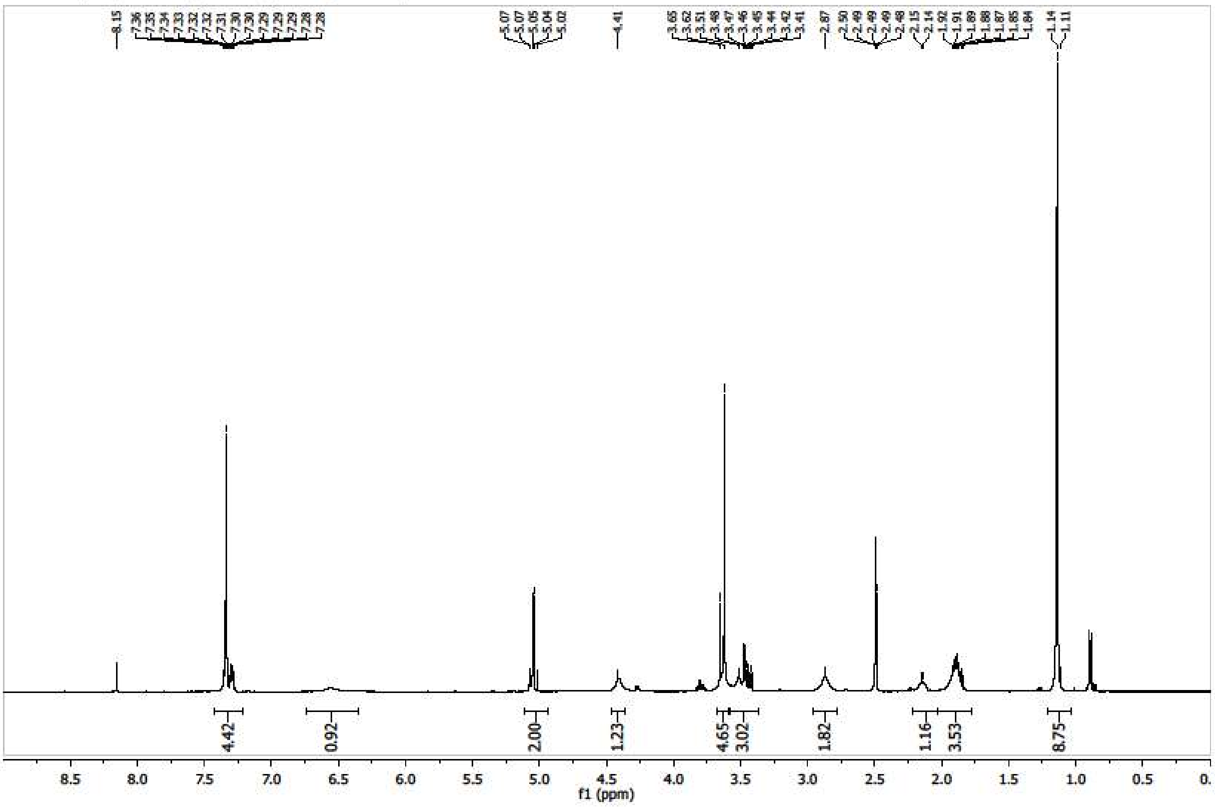

Cbz-Ser(tBu)-Pro-OMe, ^{13}C NMR, DMSO, 126 MHz, 393 K

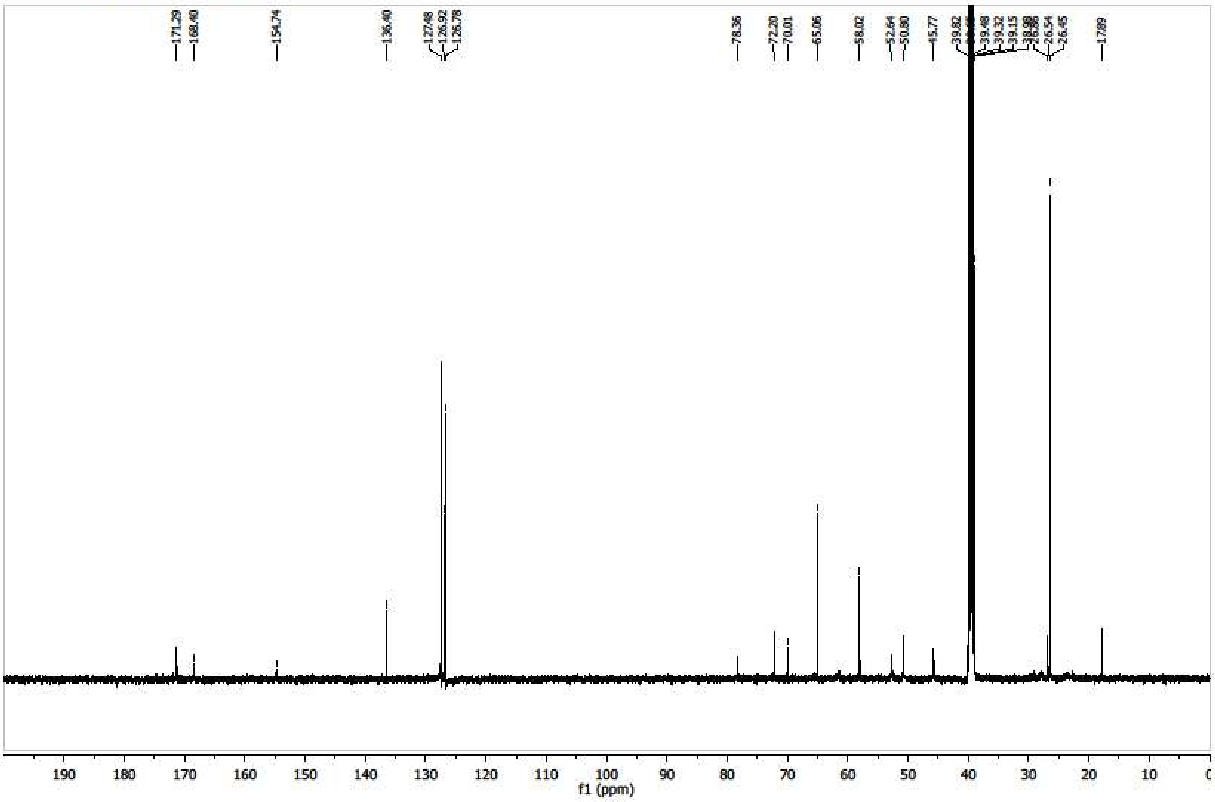

Cbz-Ser(tBu)-Pro-OMe, COSY

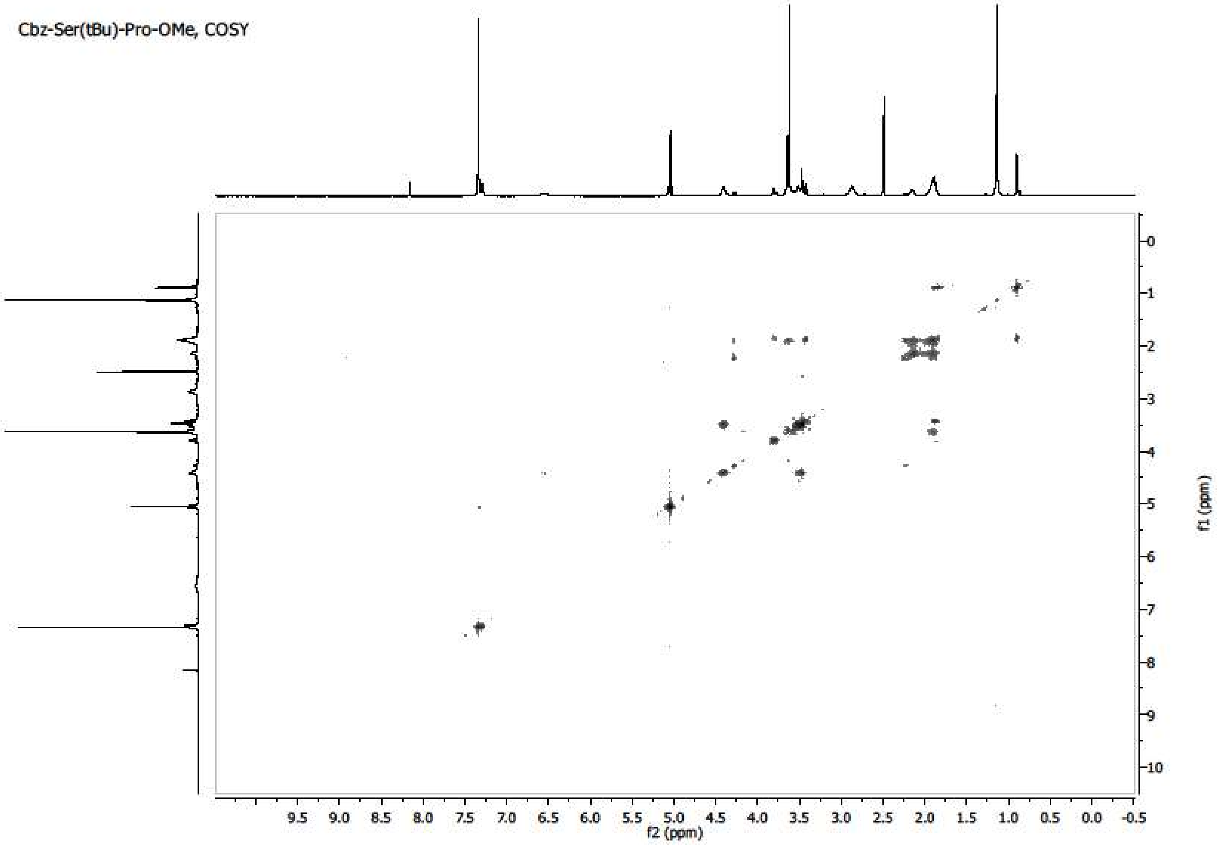

Cbz-Ser(tBu)-Pro-OMe, HSQC

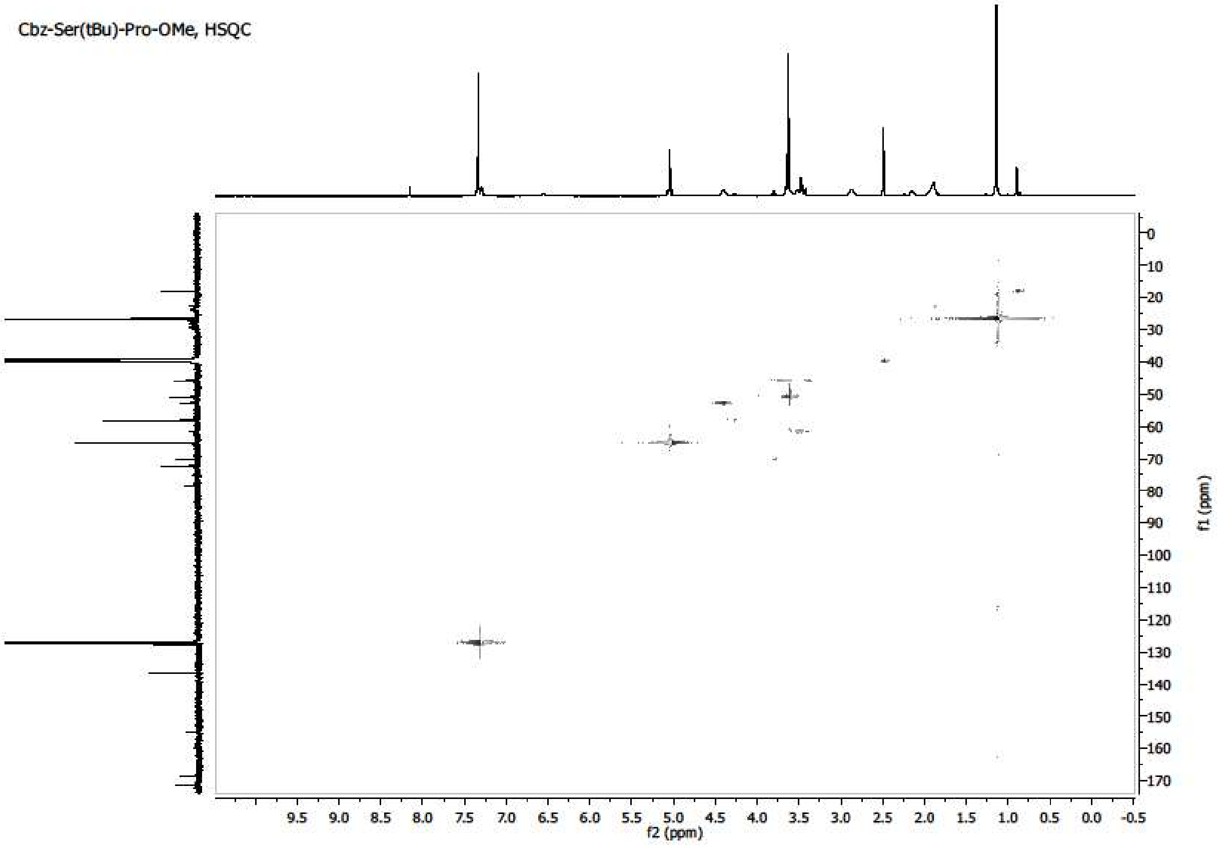

Cbz-Ser(tBu)-Pro-OMe, HMBC

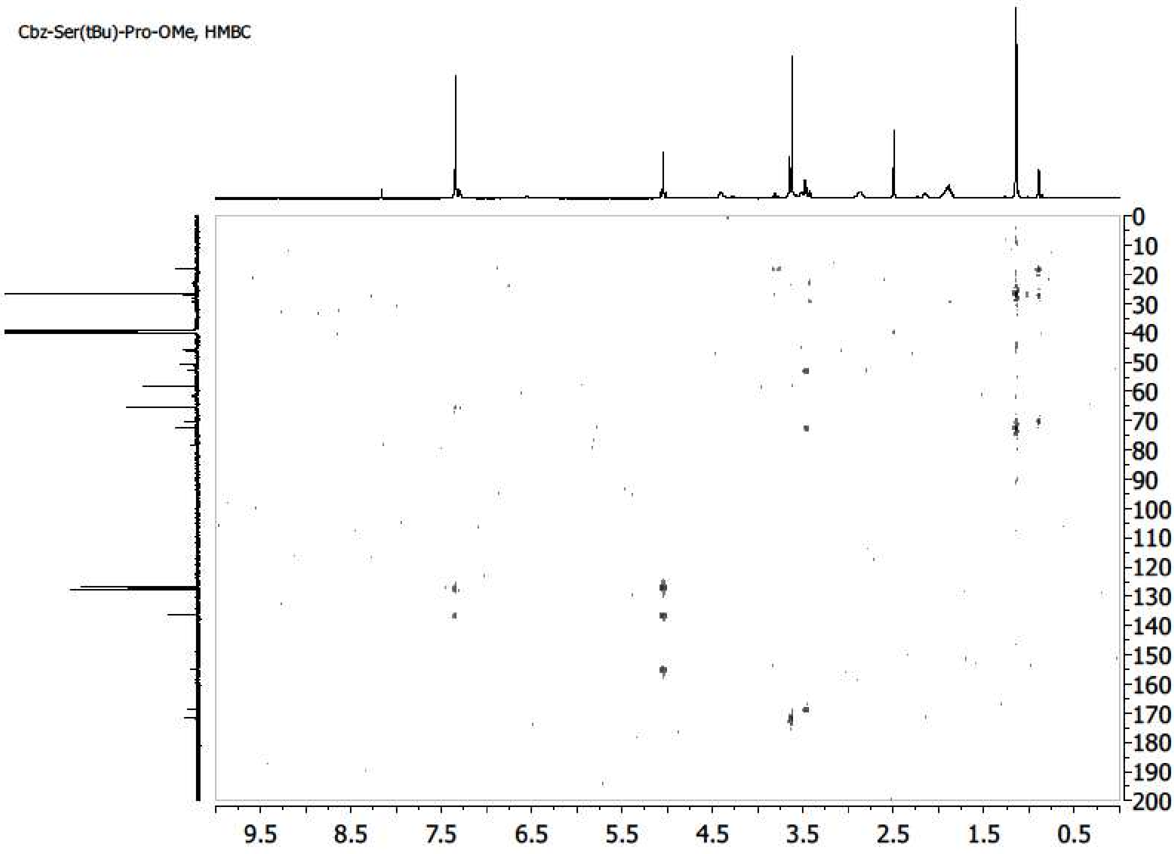

Cbz-Thr-Phe-OMe (28)

Cbz-Thr-Phe-OMe, ^{1}H NMR, $CDCl_3$, 500 MHz

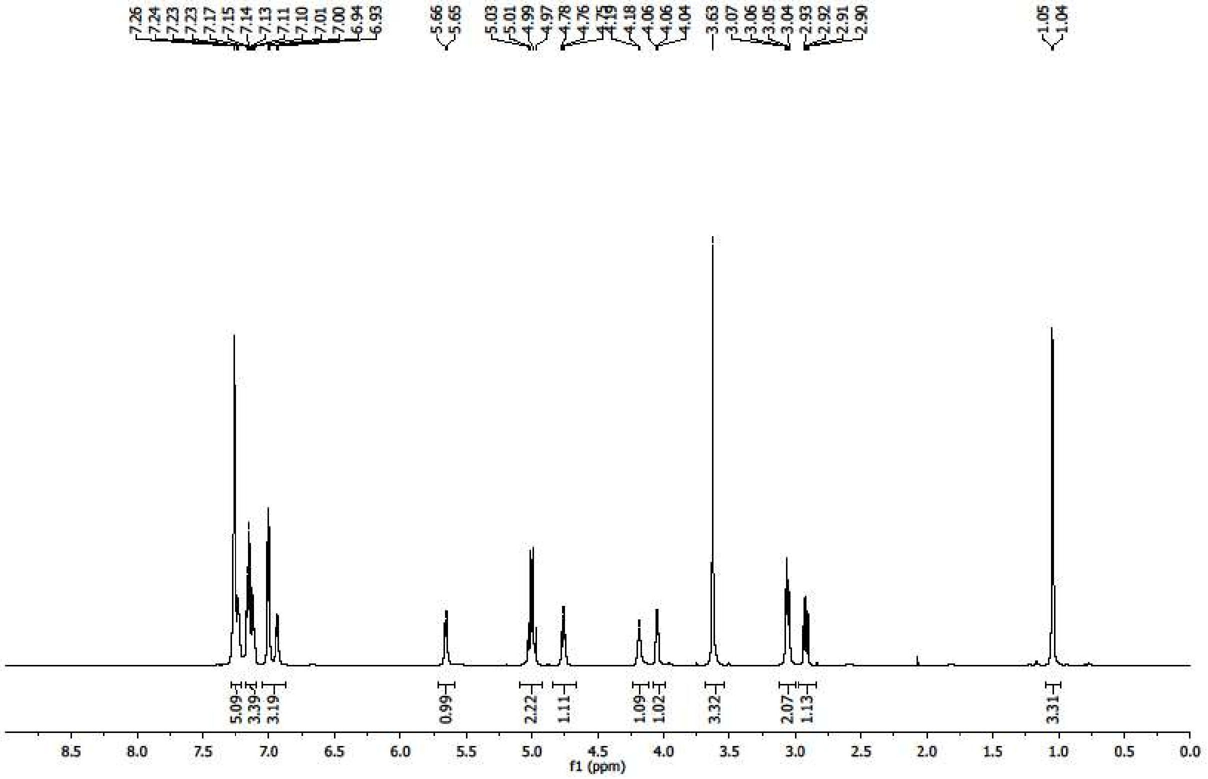

Cbz-Thr(Bzl)-Phe-OMe (30)

Cbz-Thr(Bzl)-Phe-OMe, ^{1}H NMR, $CDCl_3$, 500 MHz

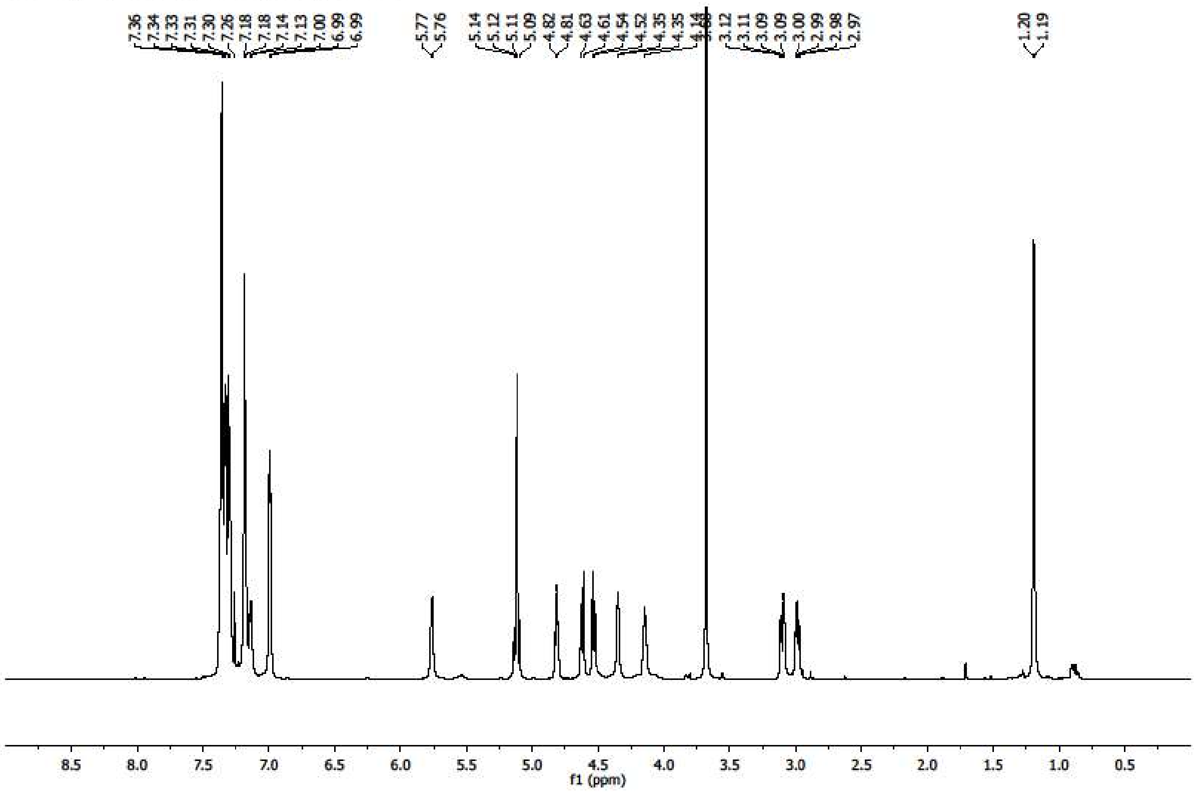

Cbz-Thr(Bzl)-Phe-OMe, ^{13}C NMR, $CDCl_3$, 500 MHz

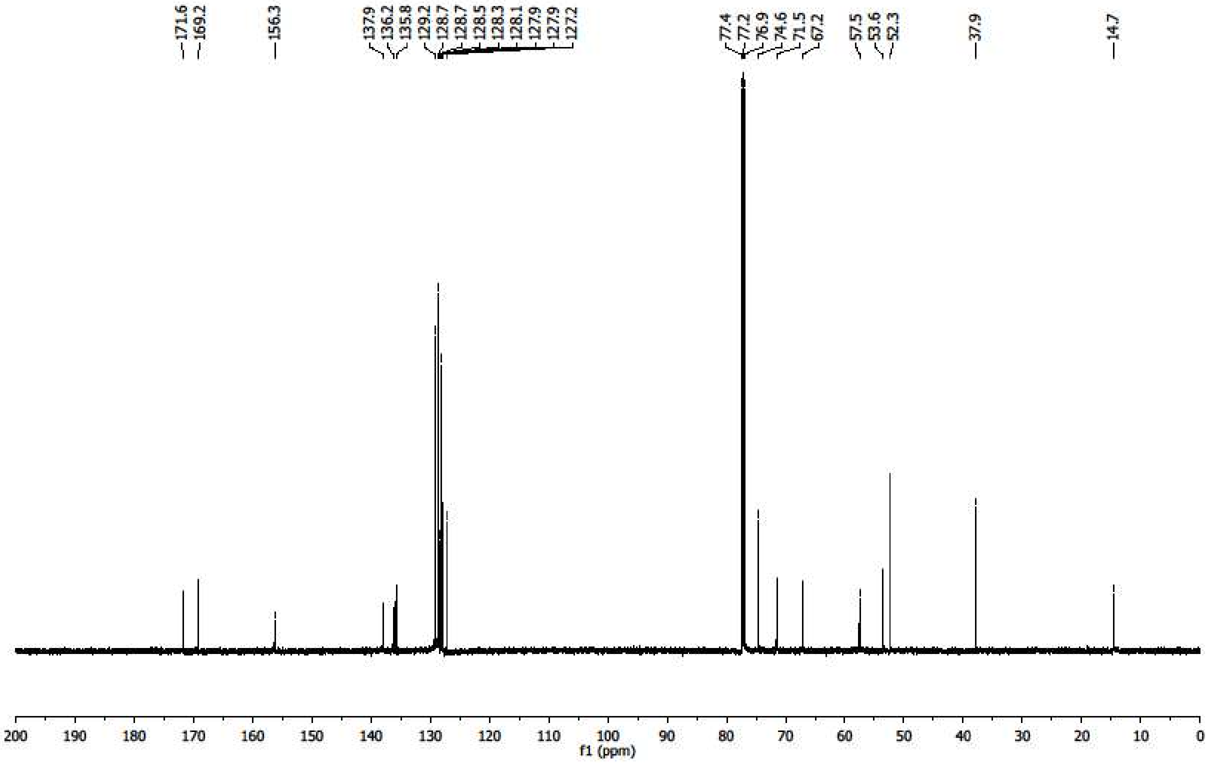

Cbz-Thr(Bzl)-Phe-OMe, COSY

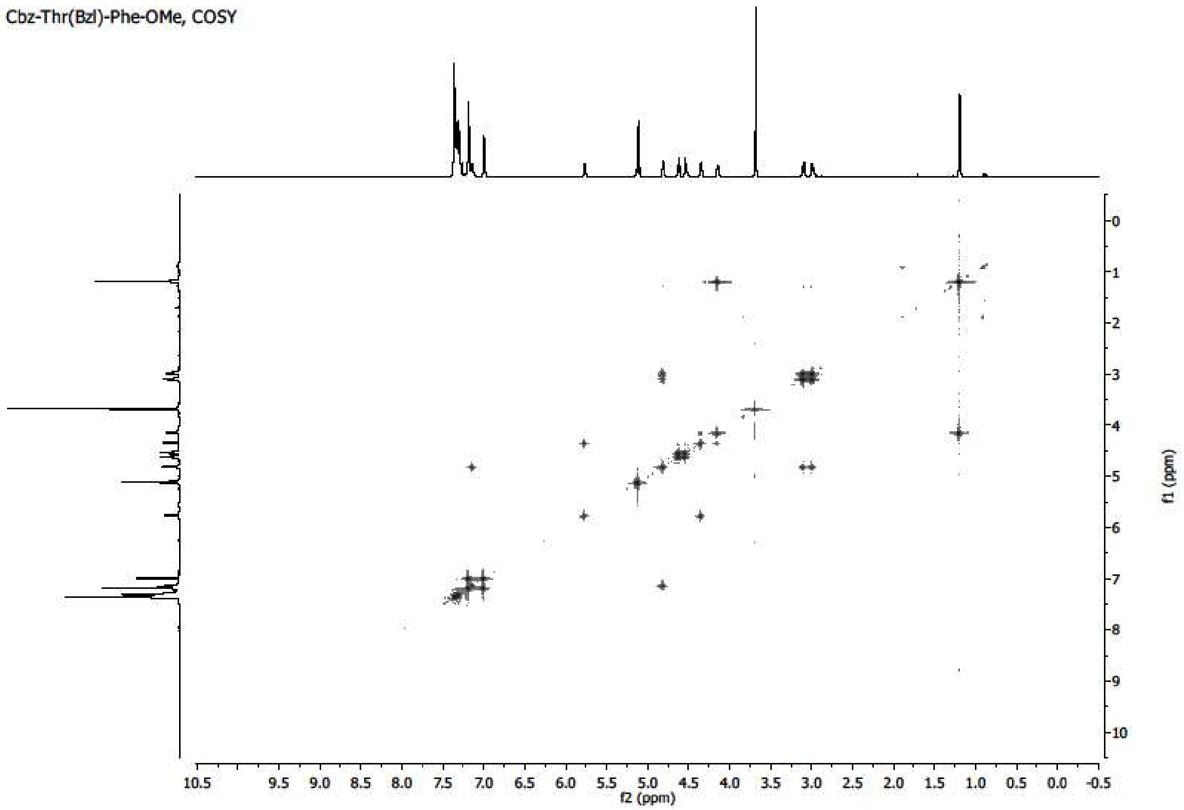

Cbz-Thr(Bzl)-Phe-OMe, HSQC

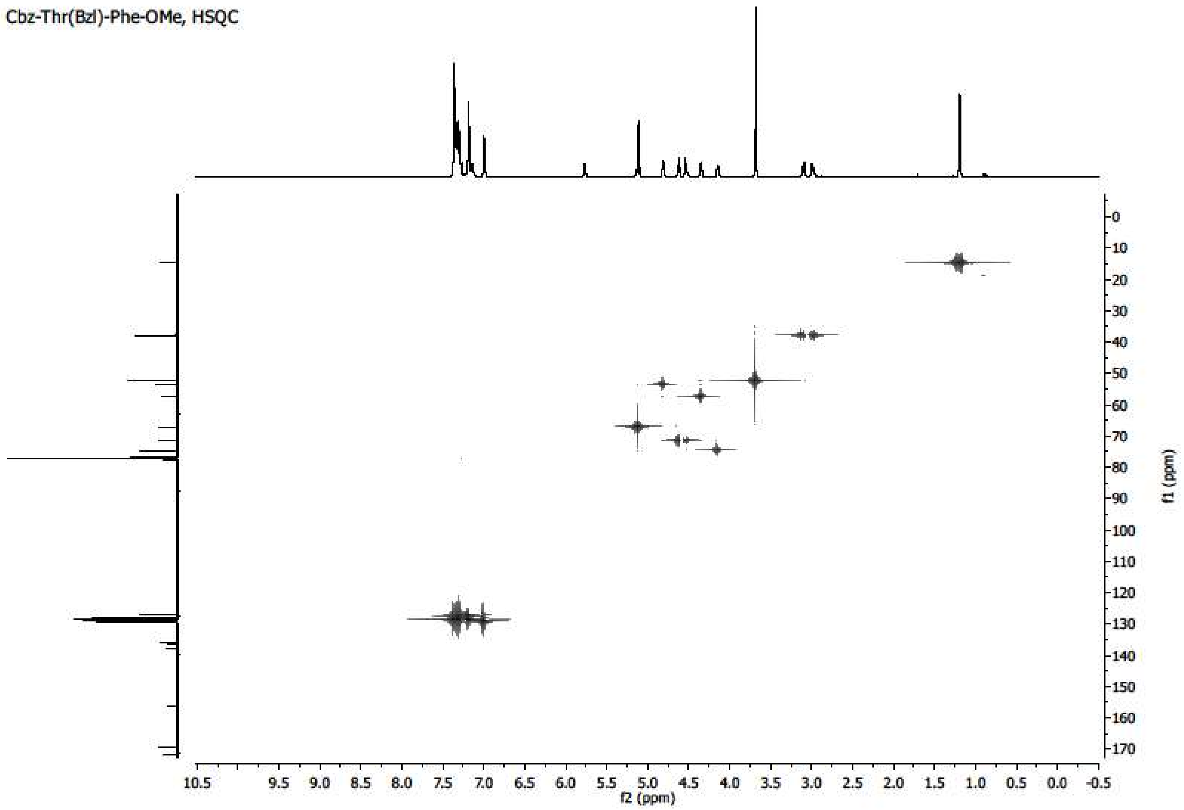

Fmoc-Ala-Ile-OMe (32)

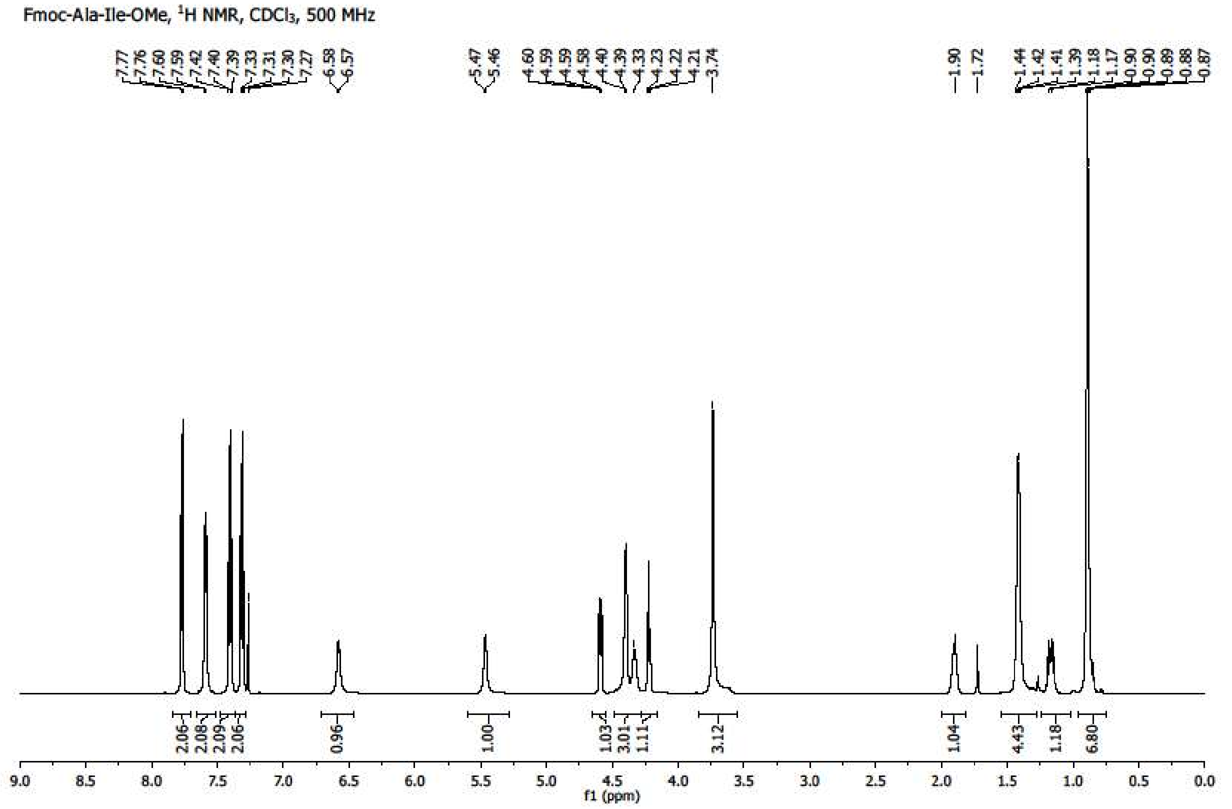

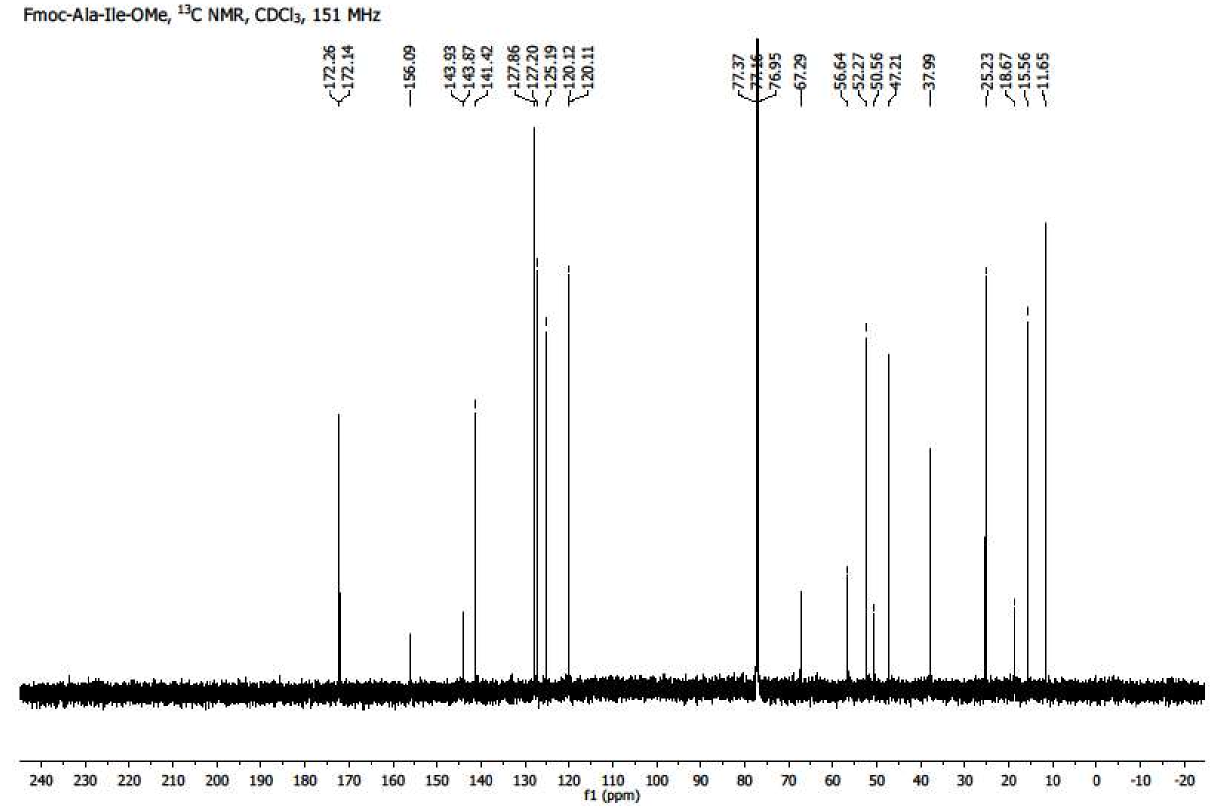

Fmoc-Ala-Ile-OMe, COSY

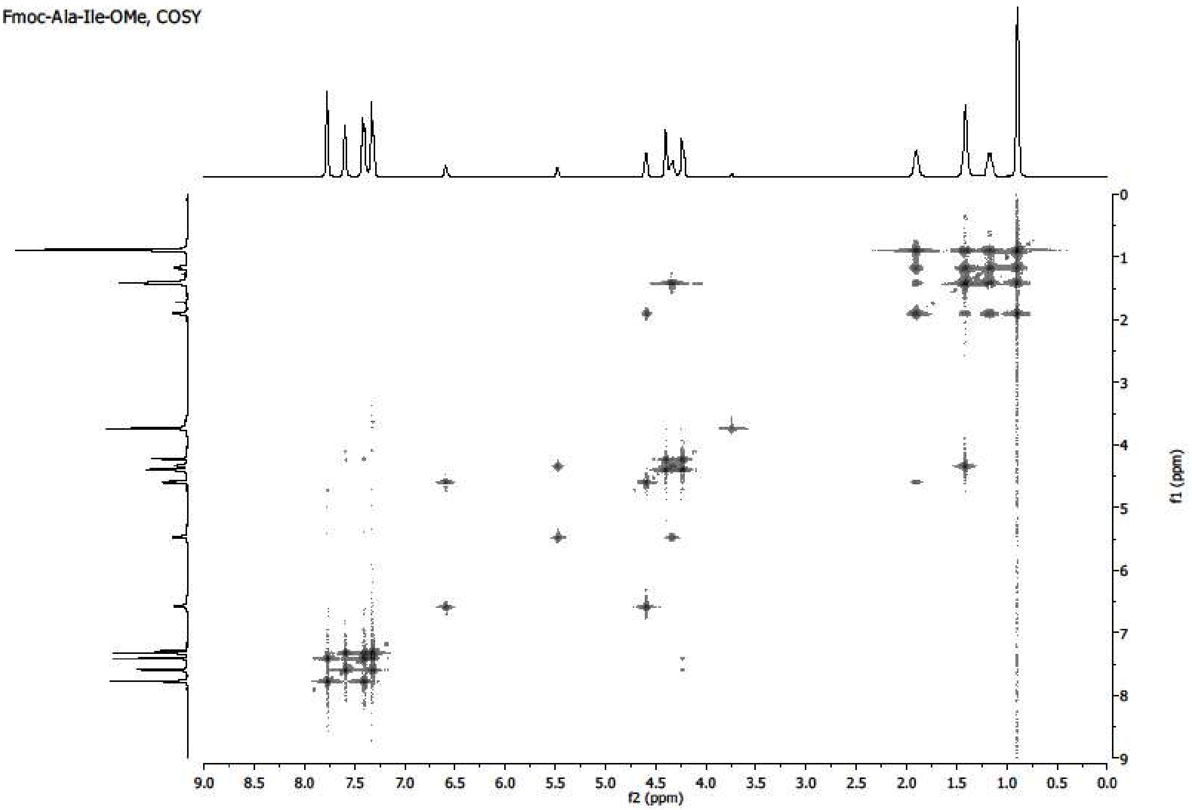

Fmoc-Ala-Ile-OMe, HSQC

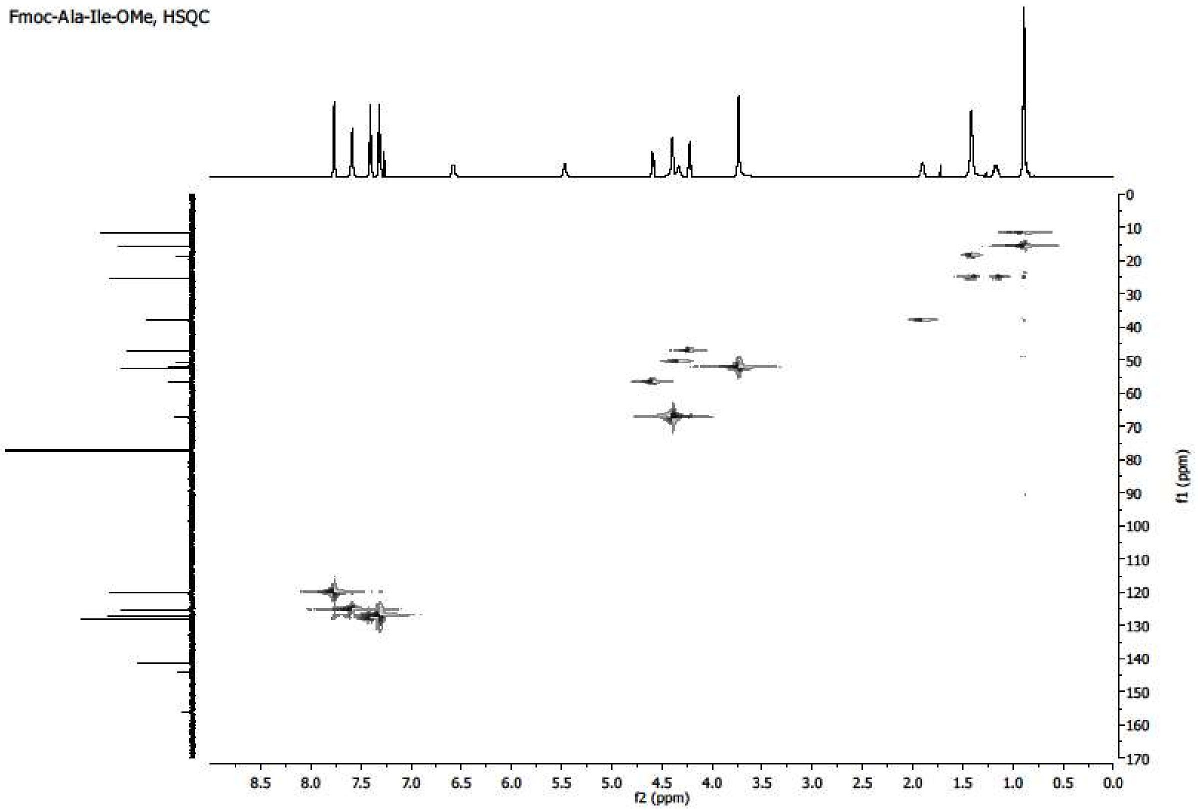

Fmoc-Leu-Ile-OMe (34)

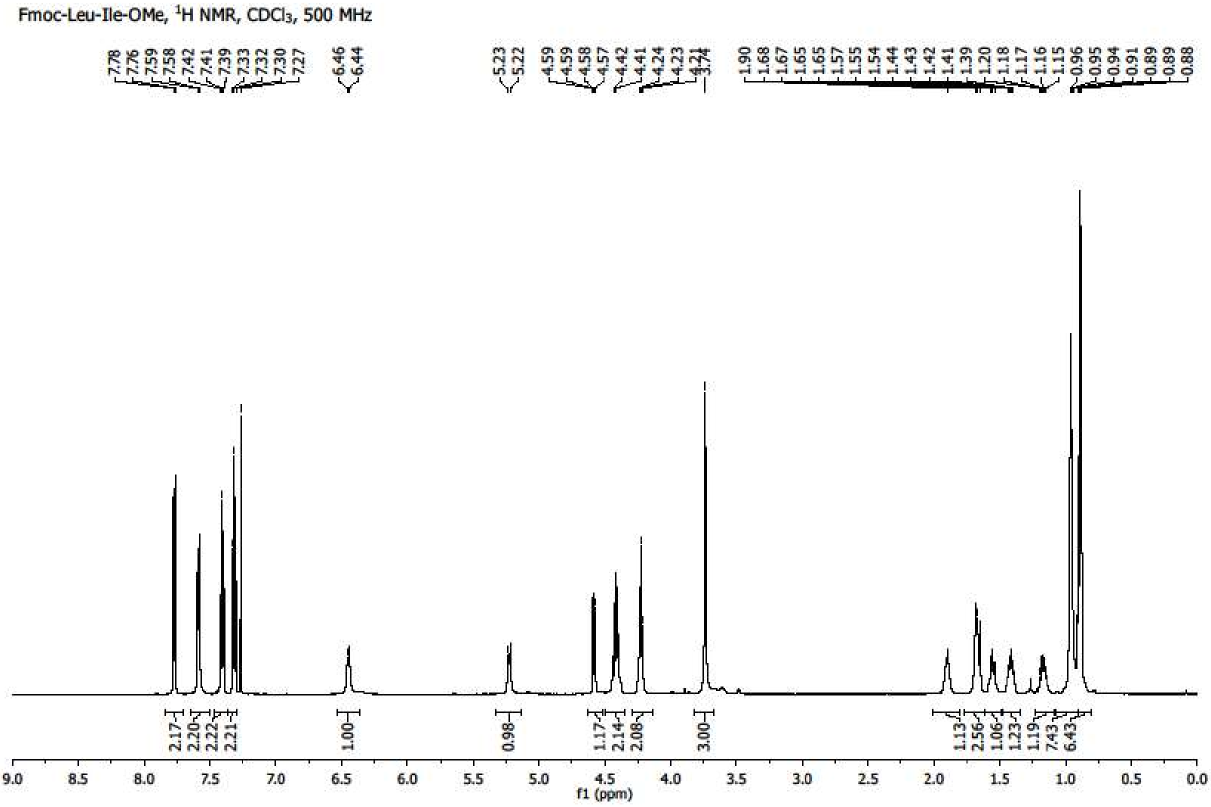

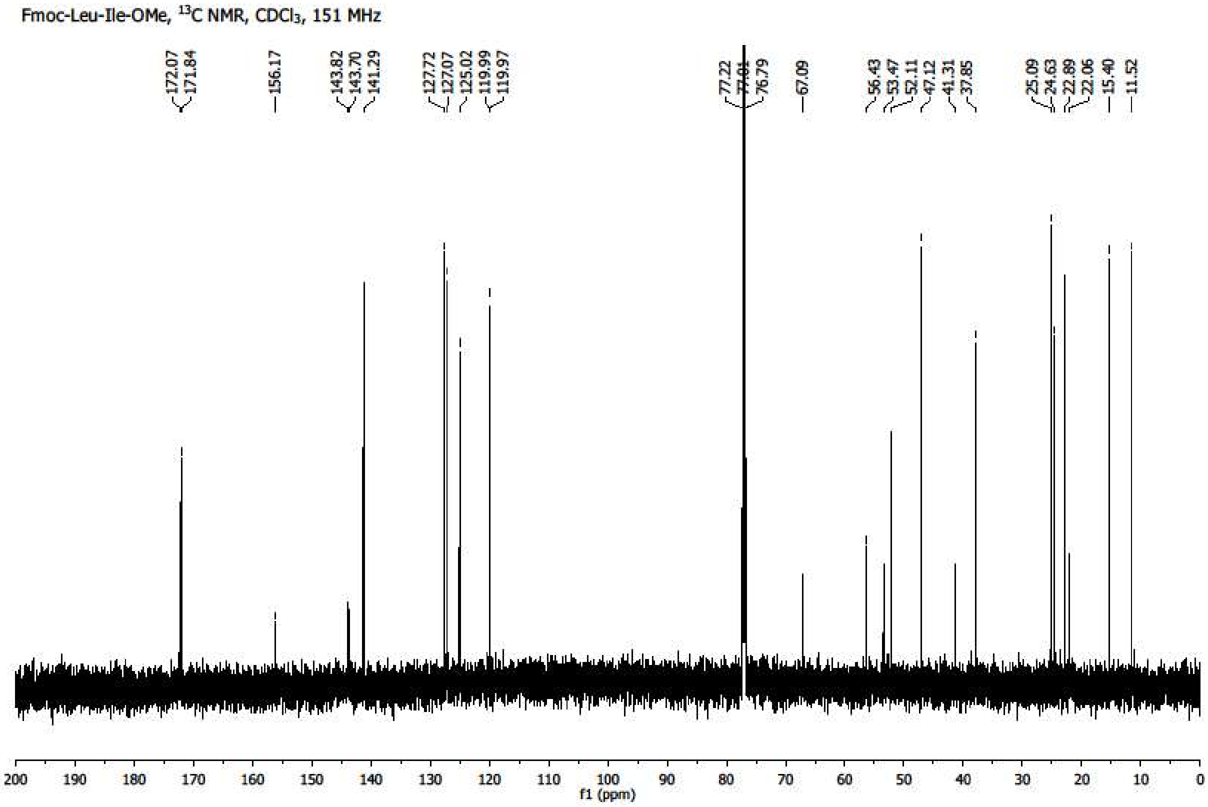

Fmoc-Leu-Ile-OMe, COSY

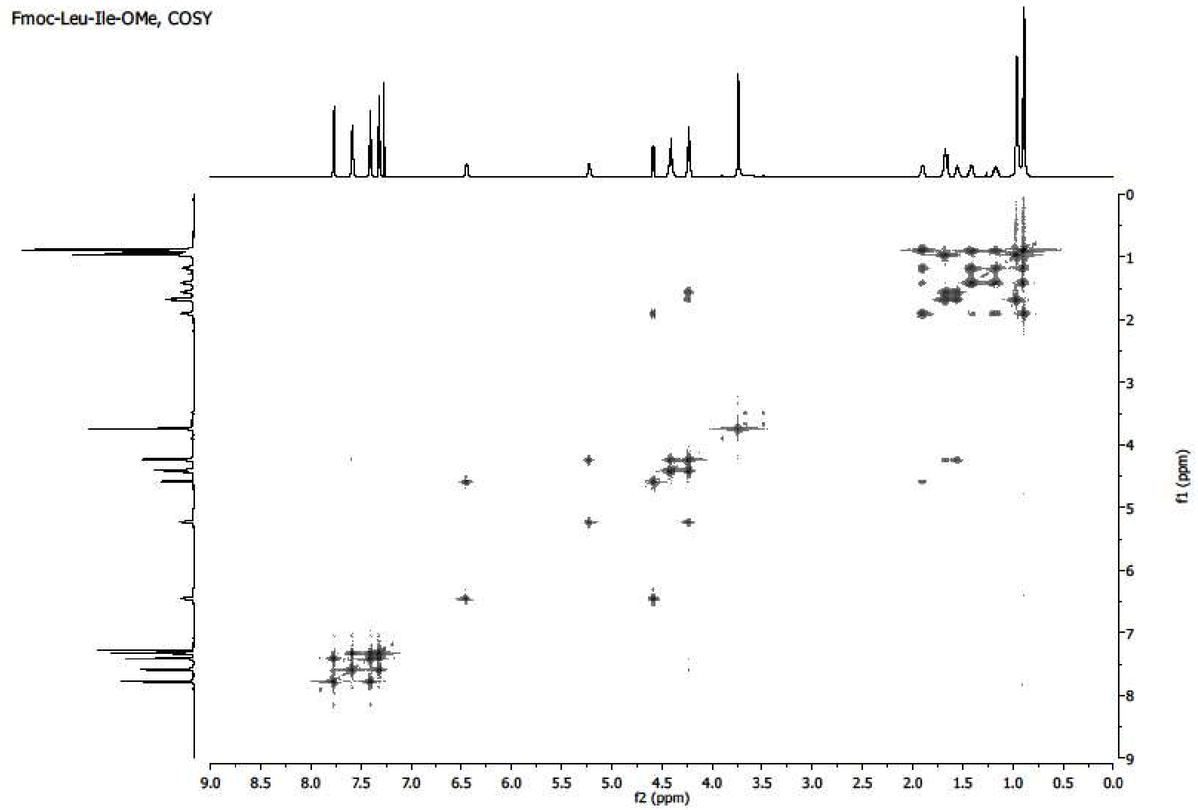

Fmoc-Leu-Ile-OMe, HSQC

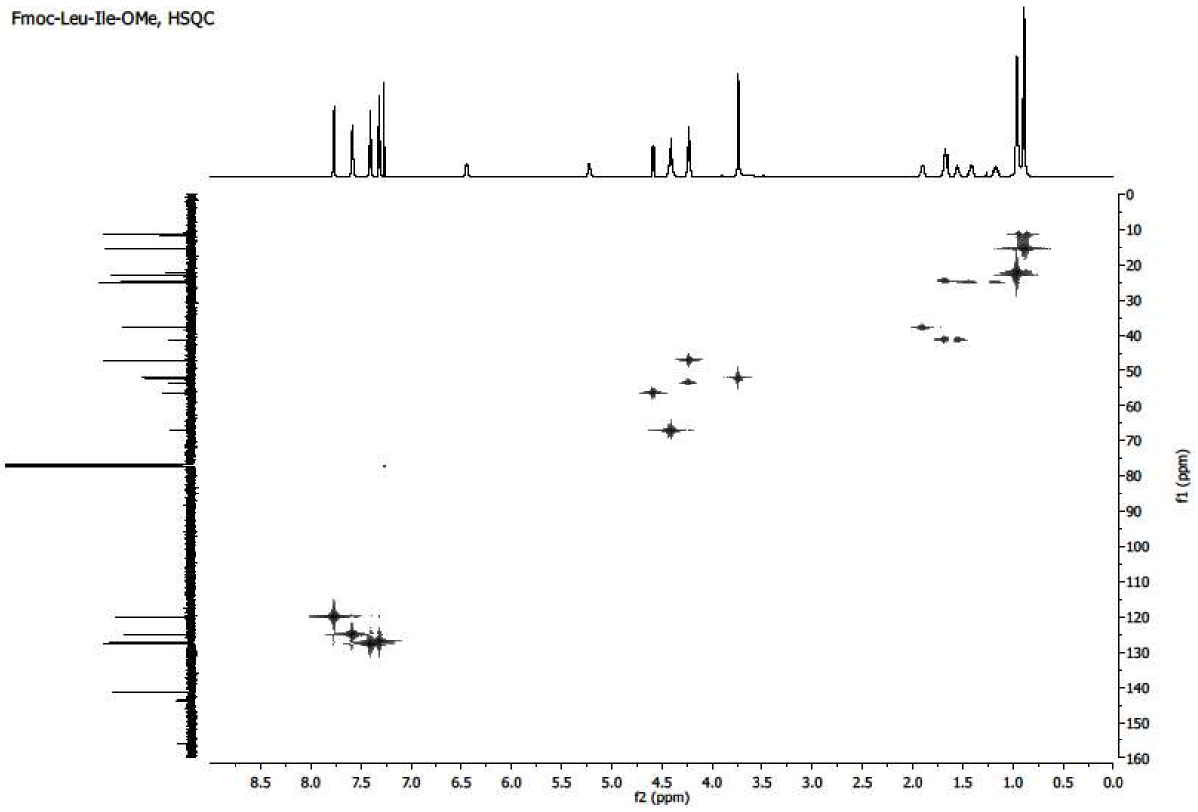

Fmoc-Tyr(OtBu)-Ala-OBn (38)

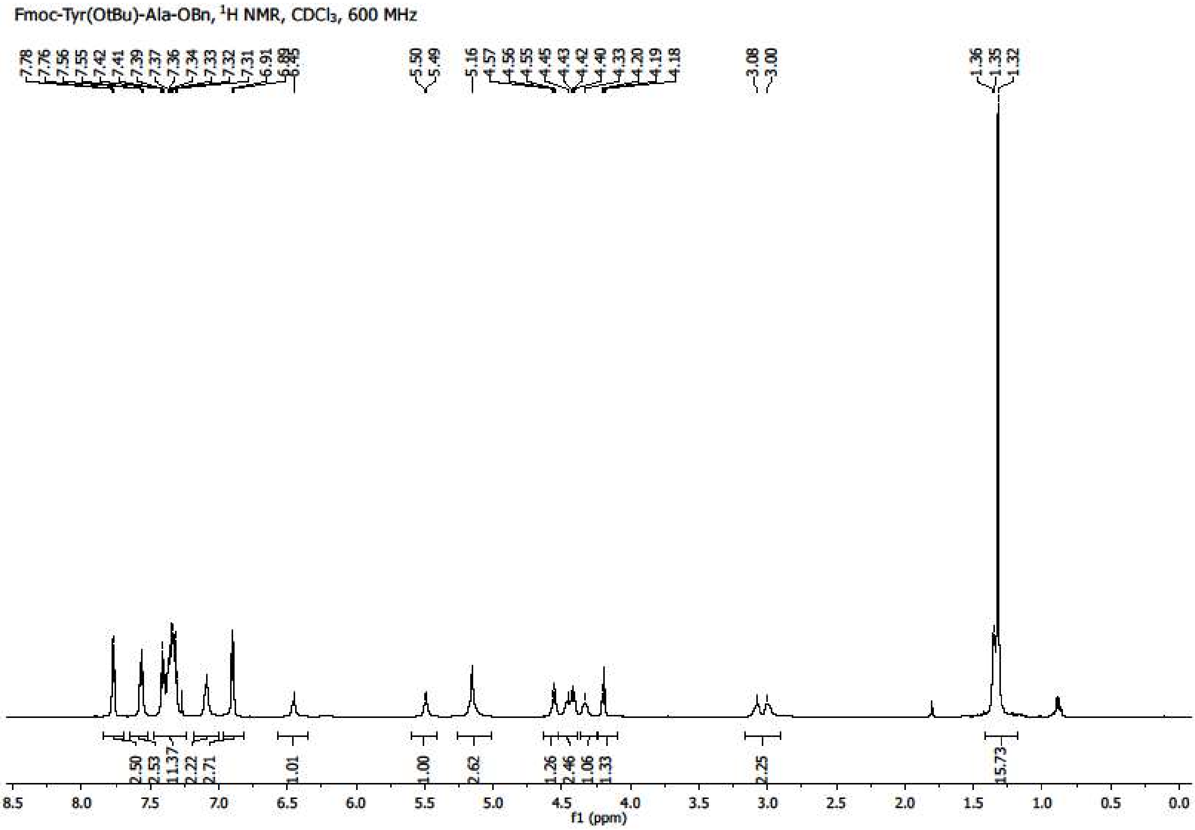

Boc-D-Ala-Val-OBn (16)

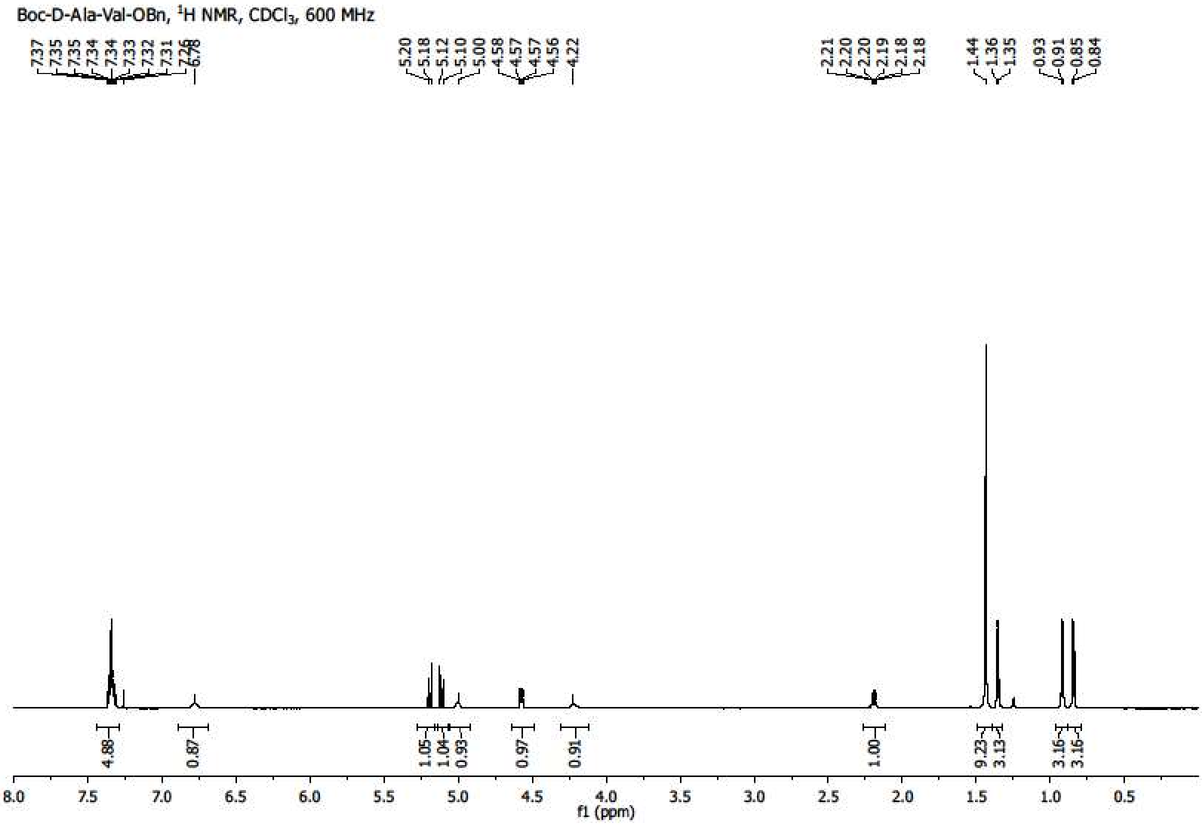

Boc-Ala-Val-OBn (17)

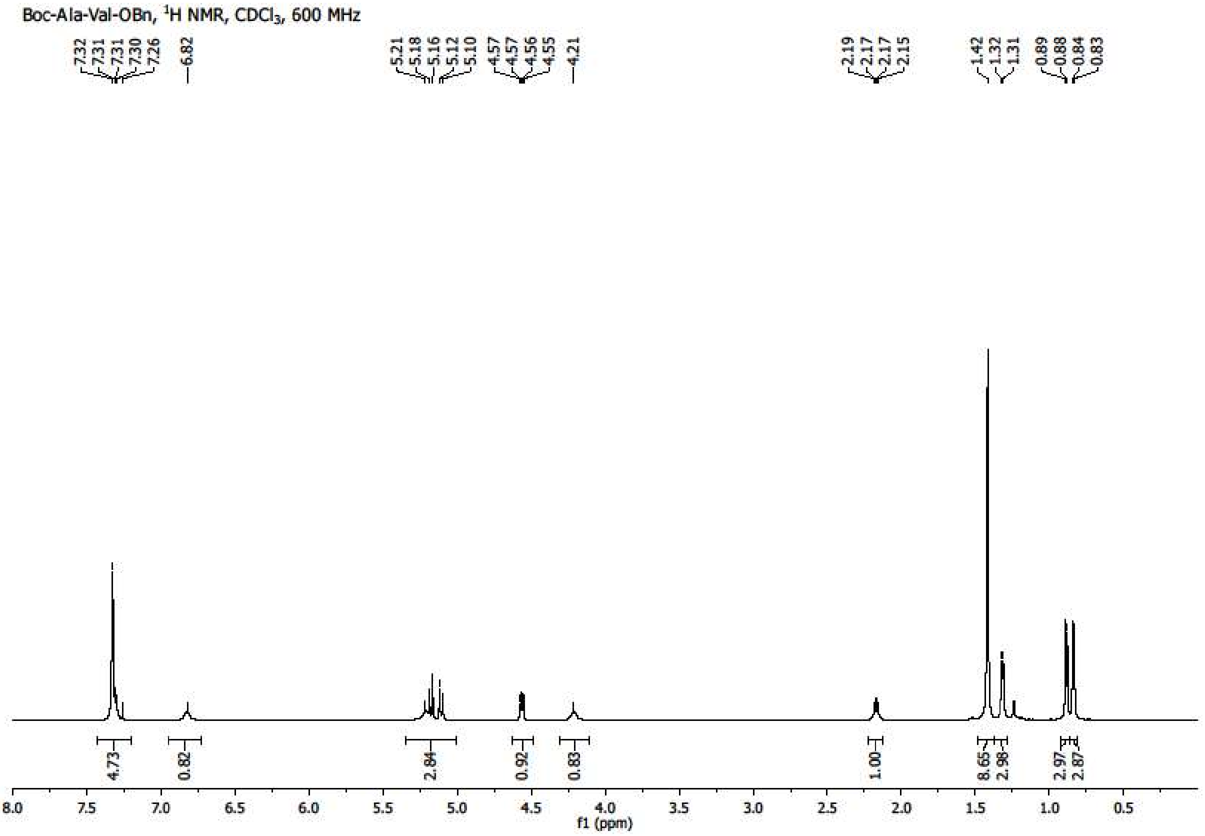

Boc-Gly-Val-OBn (43)

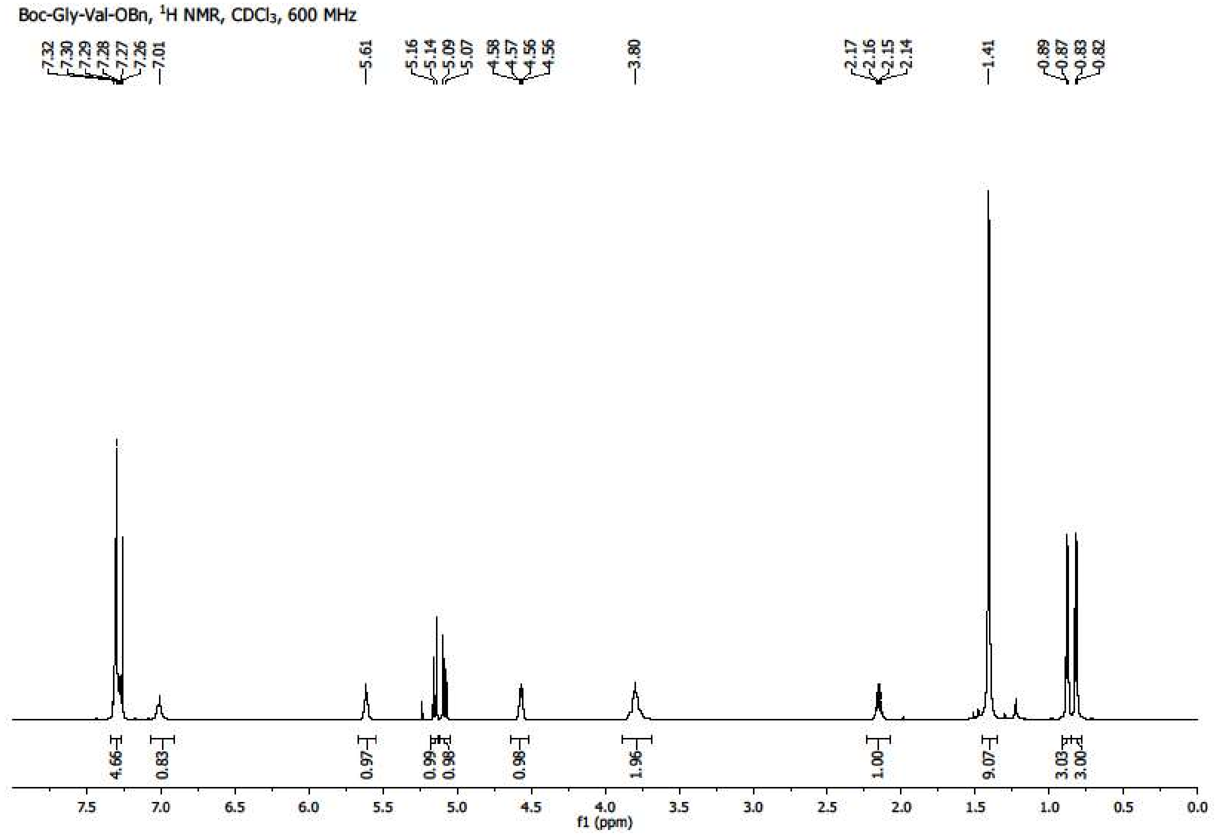

Boc-Pro-Val-OBn (45)

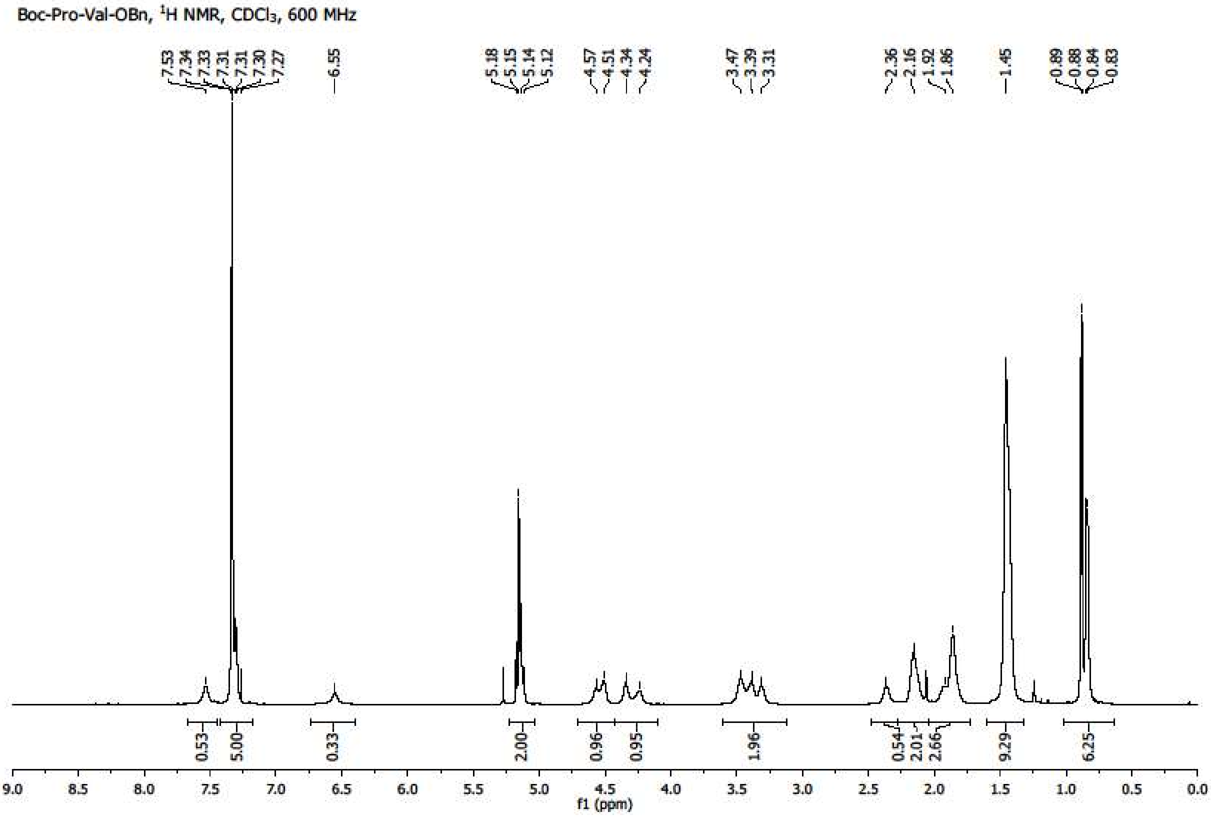

Boc-Trp(H)-Ala-OBn (47)

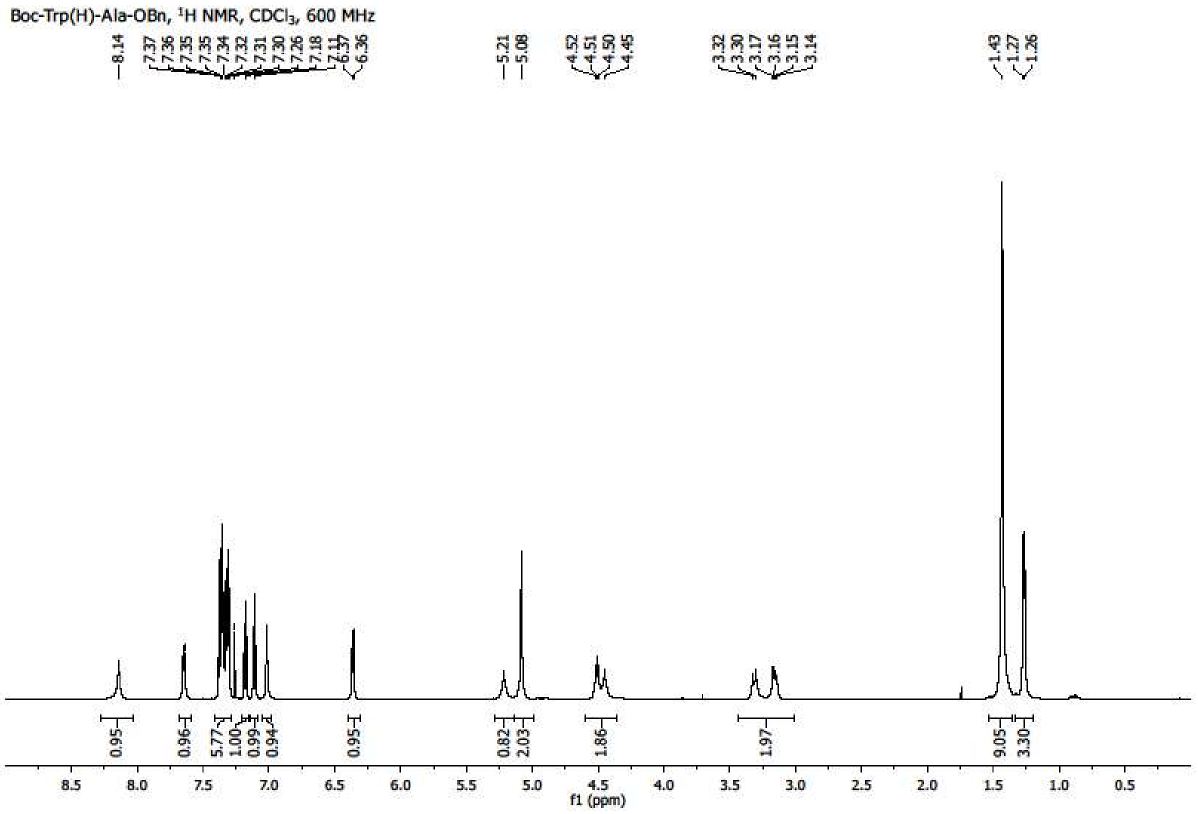

Boc-Trp(H)-Ala-OBn, ^{1}H NMR, $CDCl_3$, 151 MHz

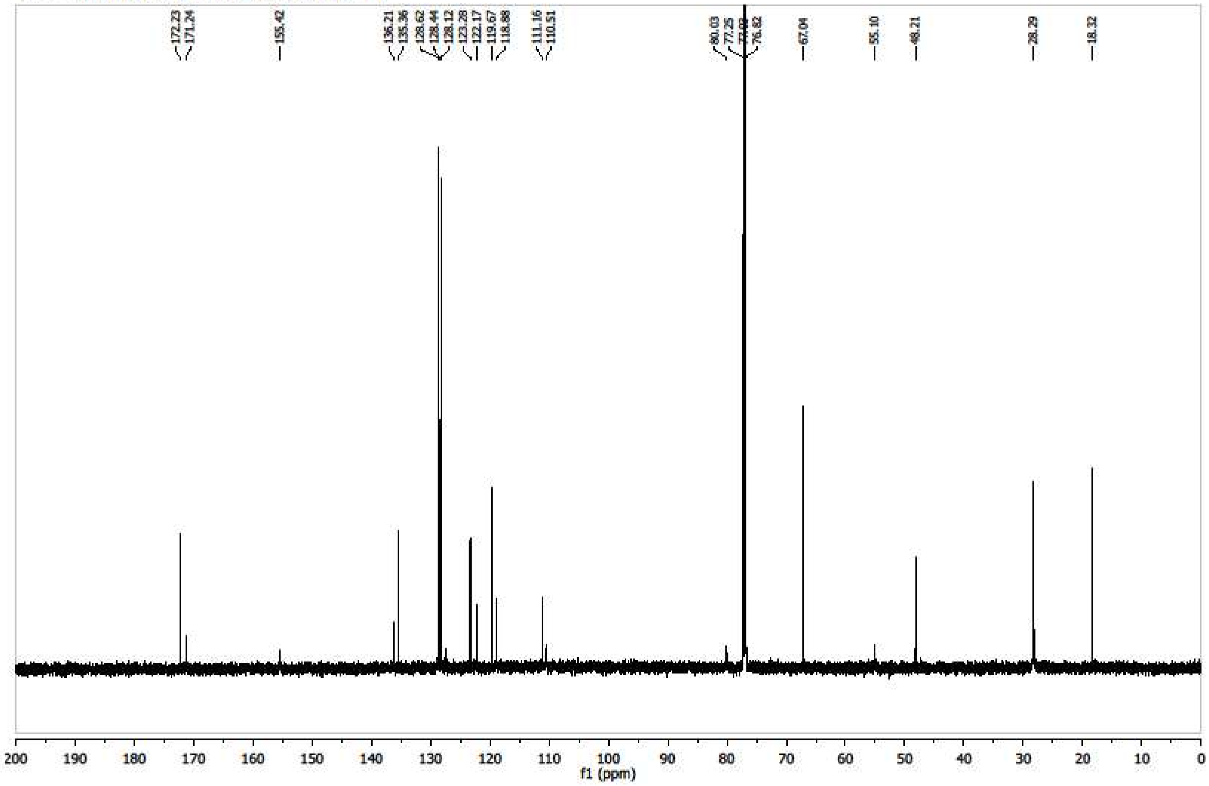

Boc-Trp(H)-Ala-OBn, HSQC

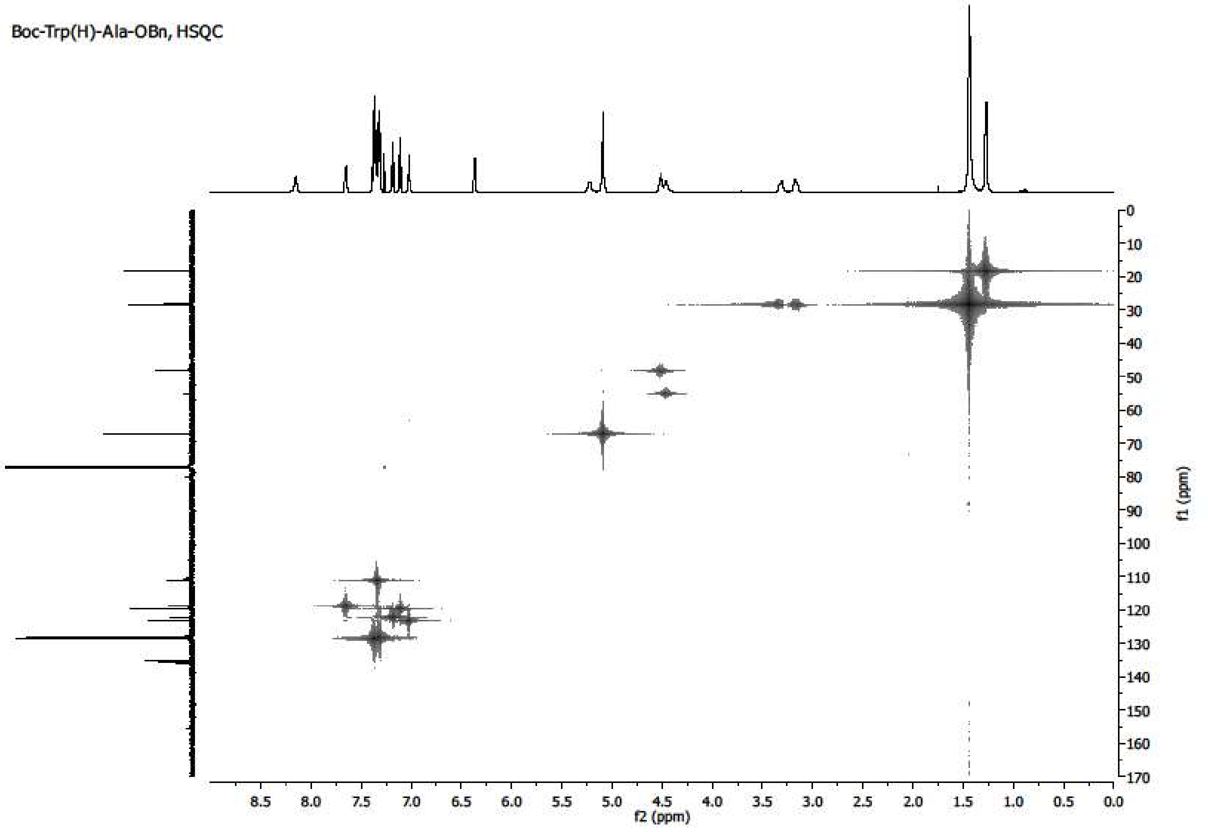

Boc-Trp(H)-Ala-Gly-Val-OBn (50)

Boc-Trp(H)-Ala-Gly-Val-OBn, ^{1}H NMR, DMSO, 500 MHz, 373 K

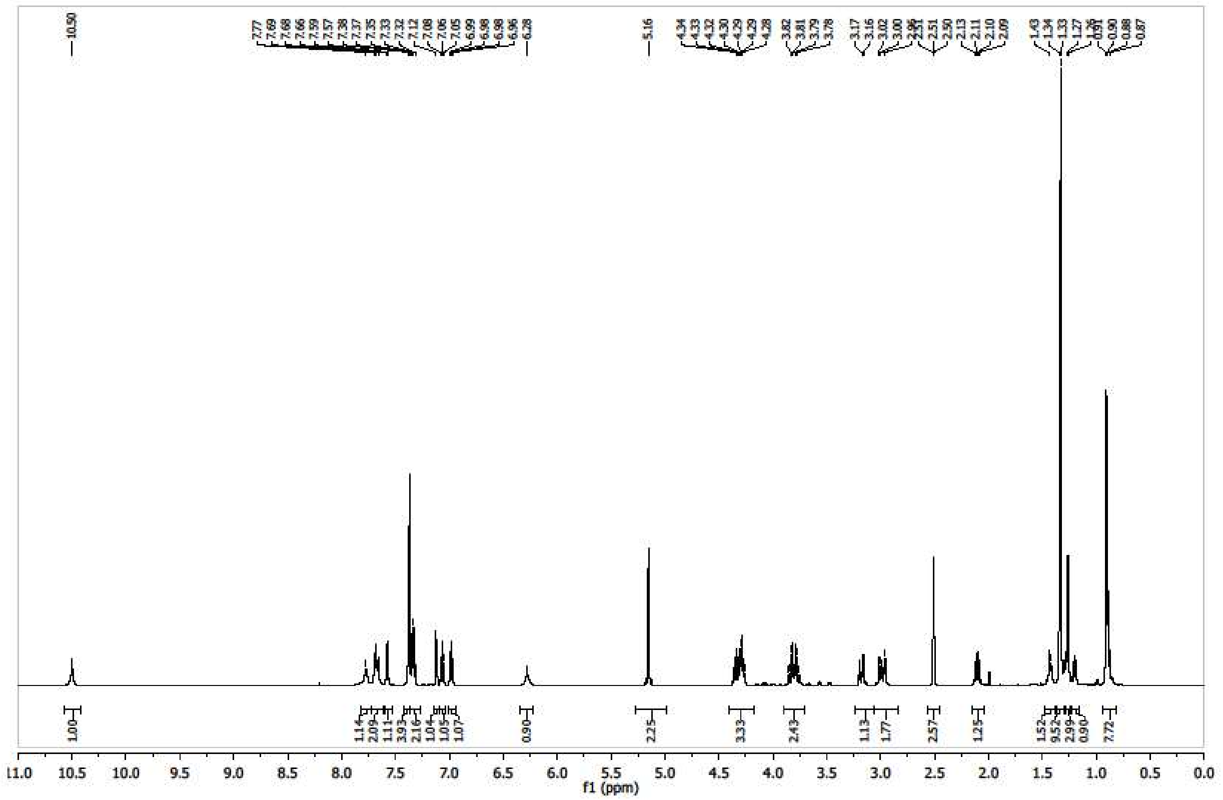

Boc-Trp(H)-Ala-Gly-Val-OBn, ^{1}H NMR, DMSO, 126 MHz, 373 K

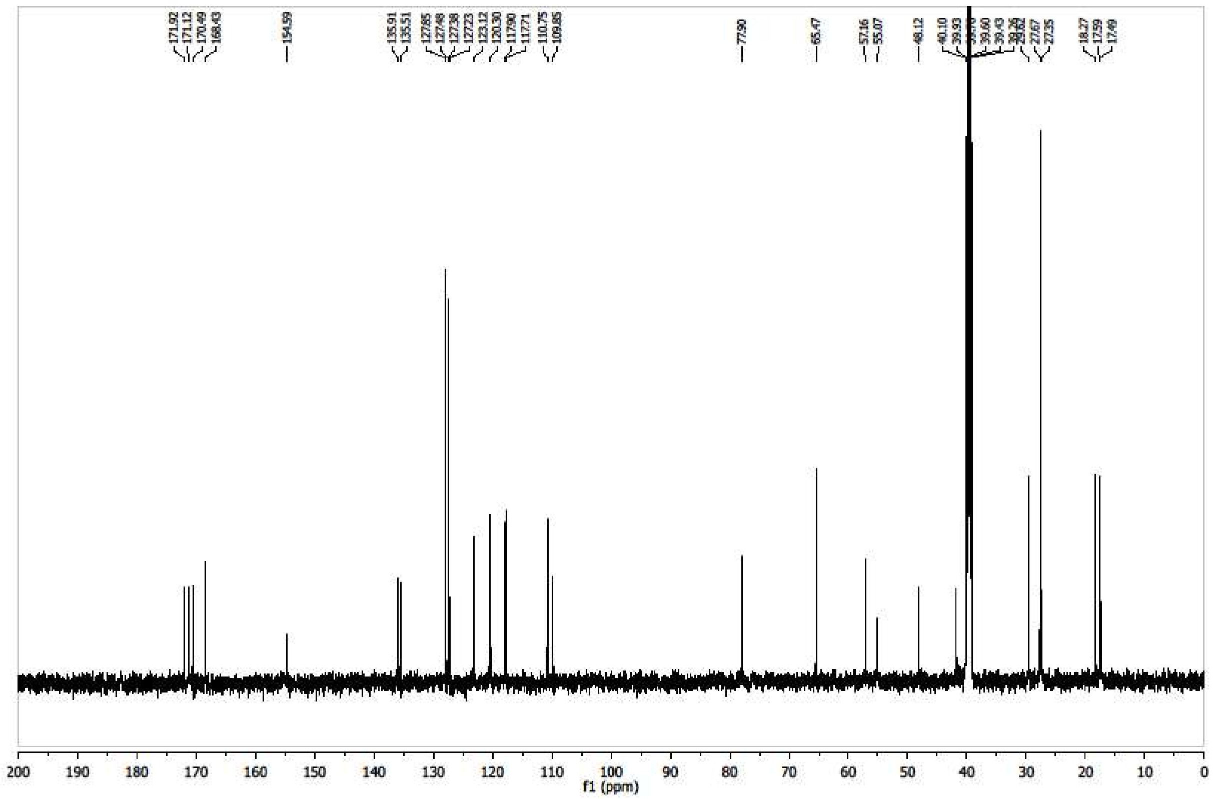

Boc-Trp(H)-Ala-Gly-Val-OBn, COSY

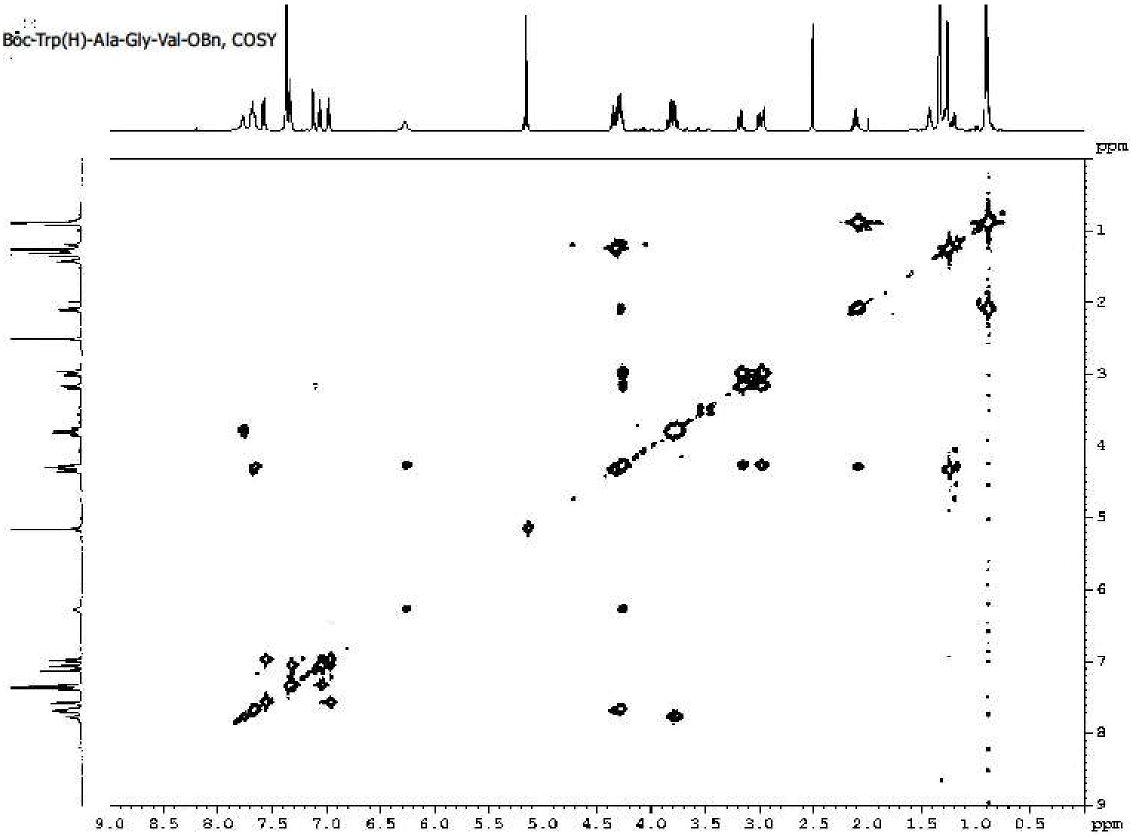

Boc-Trp(H)-Ala-Gly-Val-OBn, HSQC

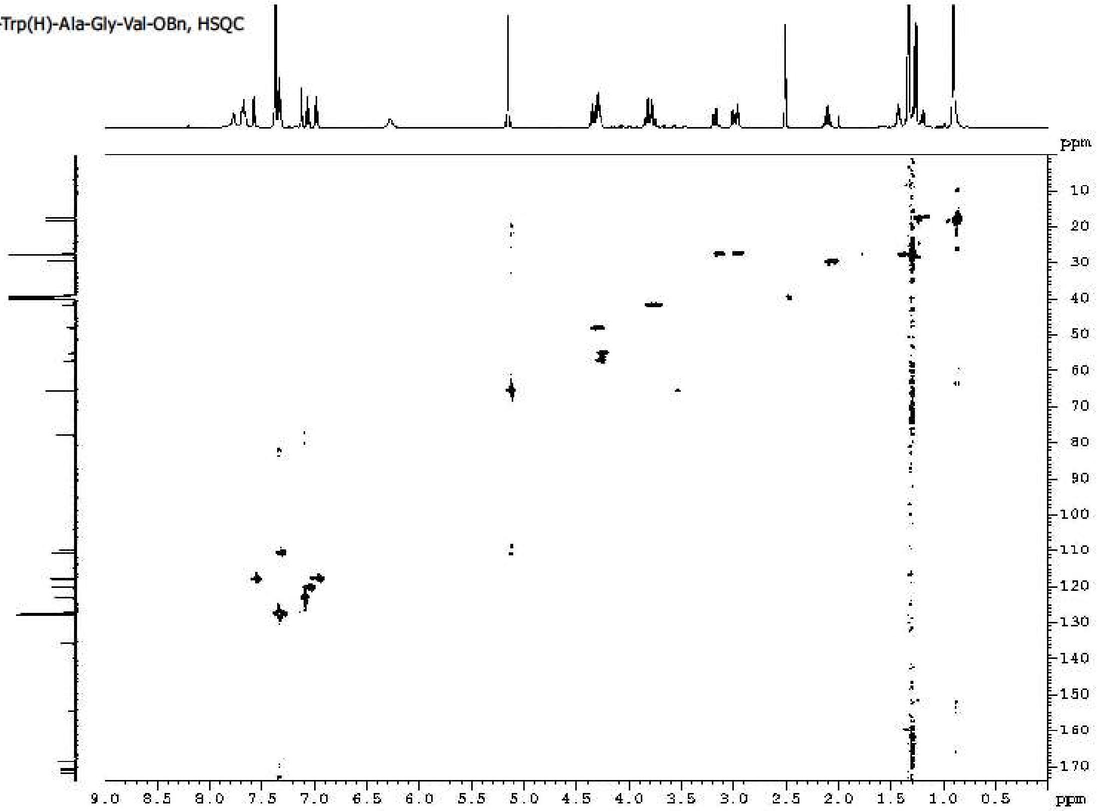

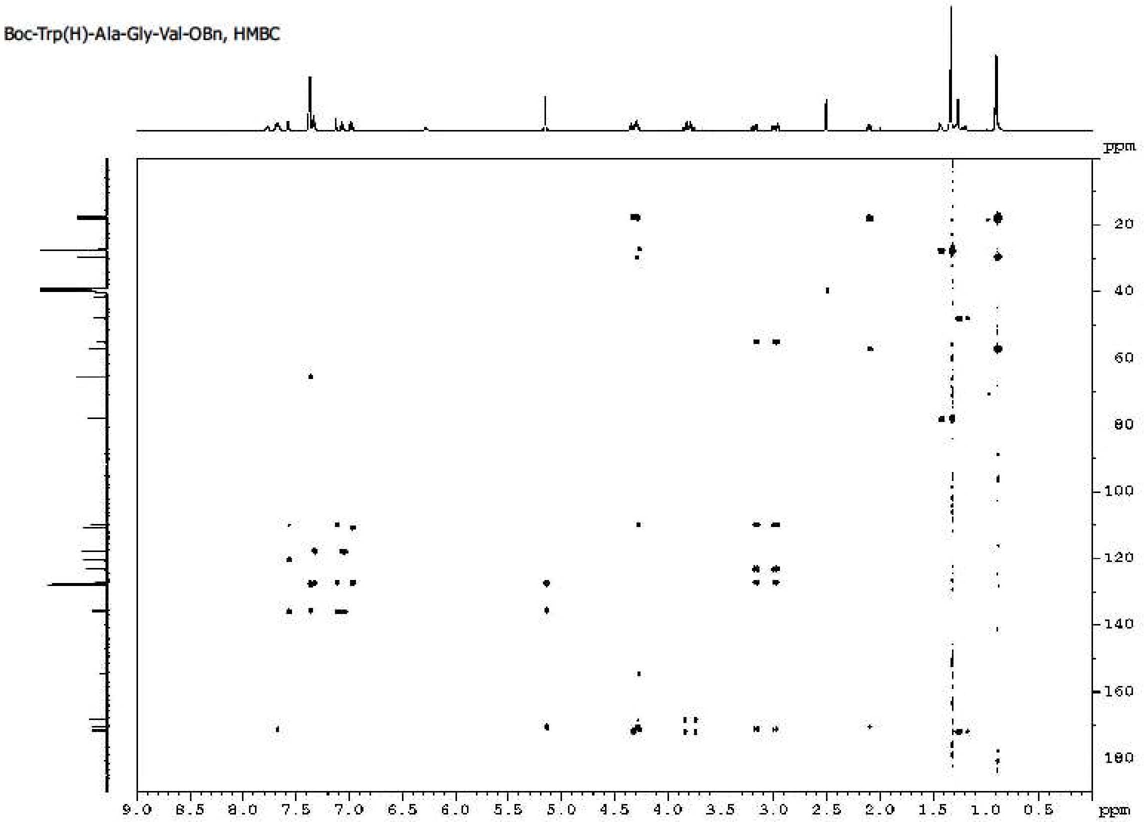

Boc-Pro-Val-Trp(H)-Ala-OBn (53)

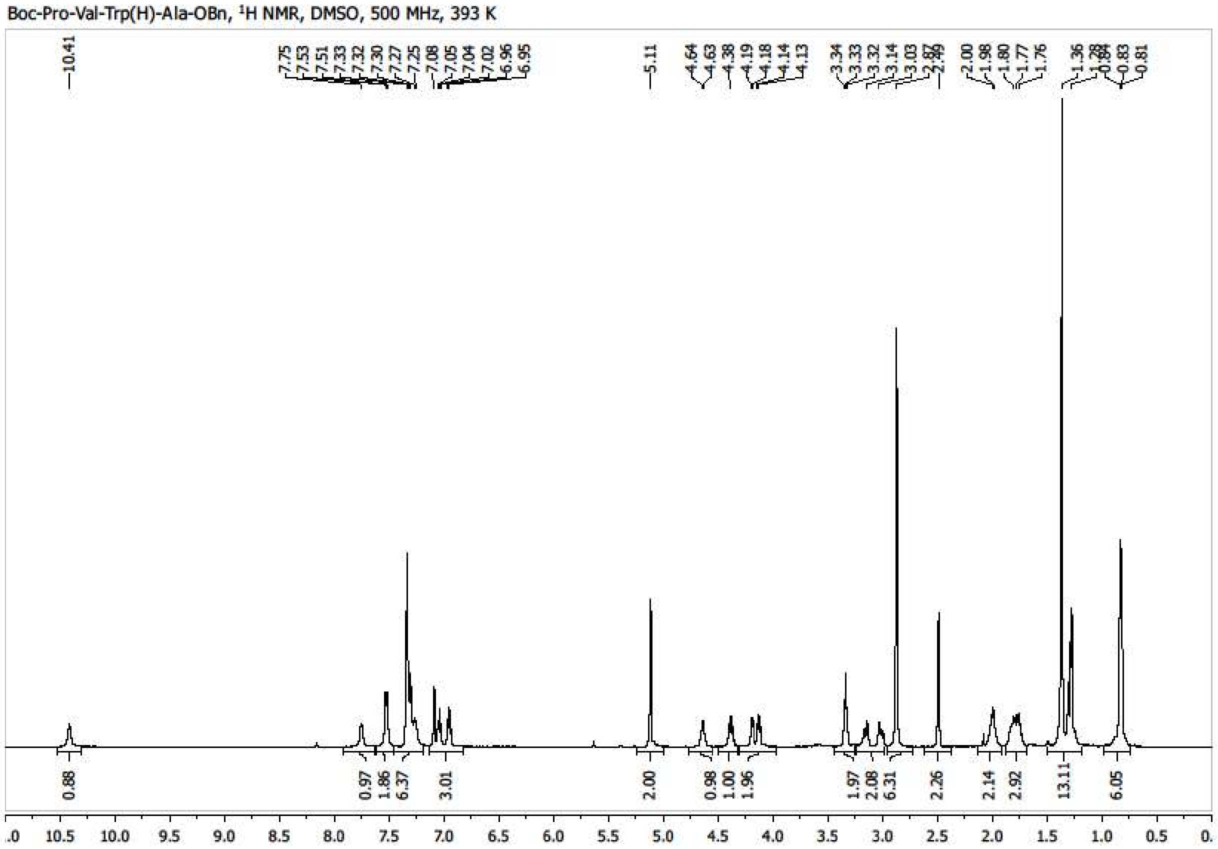

Boc-Pro-Val-Trp(H)-Ala-OBn, ^{13}C NMR, DMSO, 126 MHz, 393 K

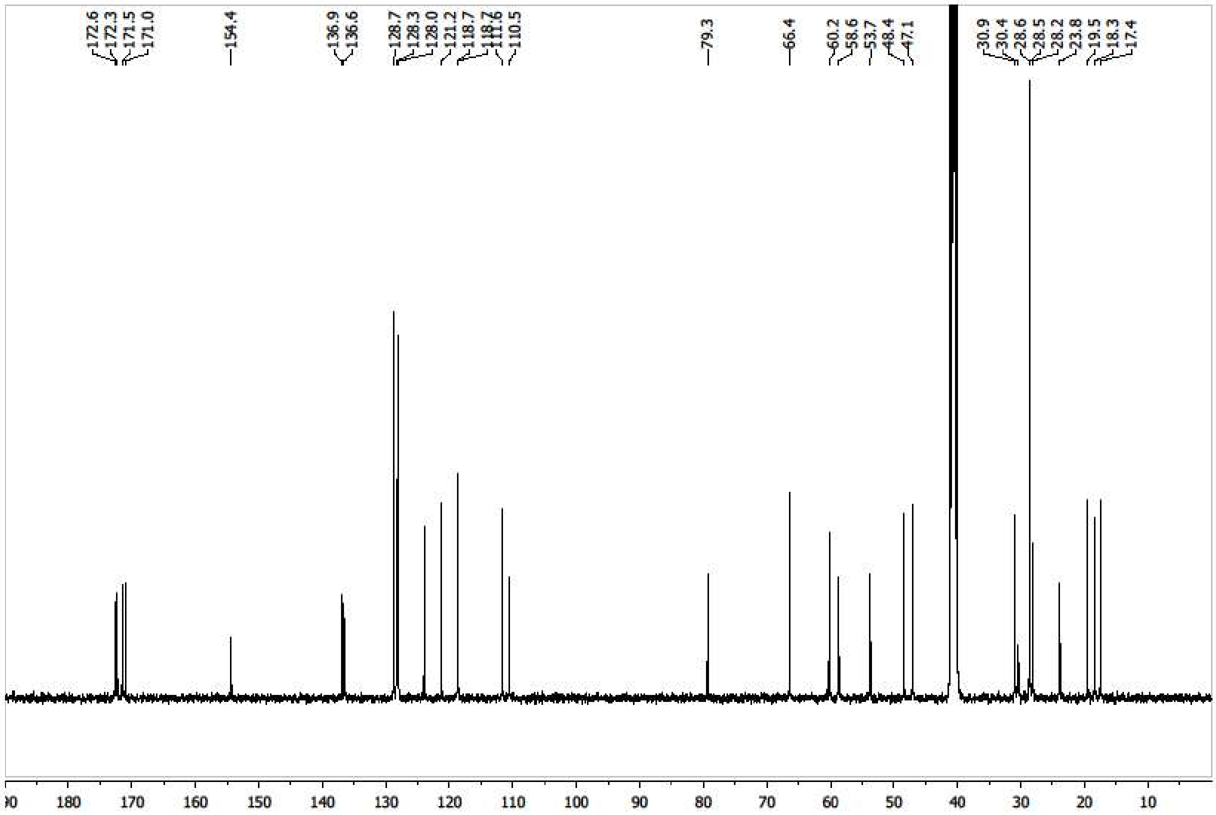

Boc-Pro-Val-Trp(H)-Ala-OBn, COSY

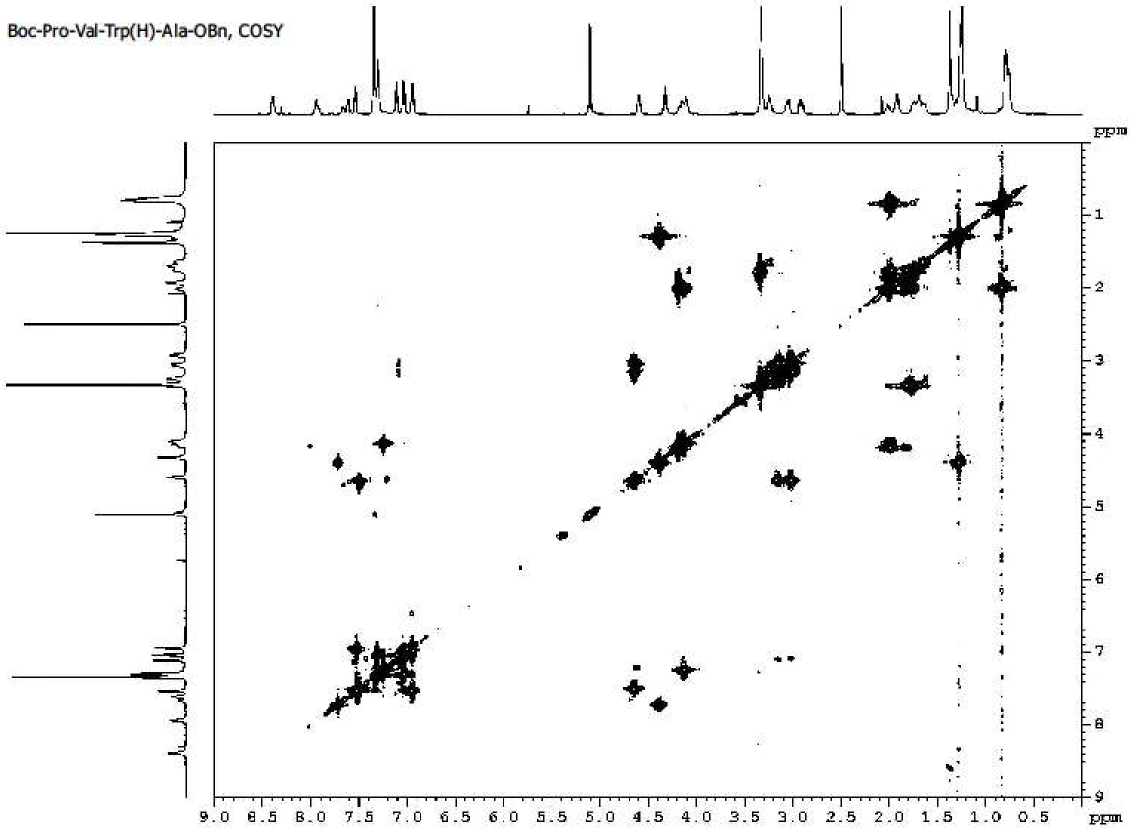

Boc-Pro-Val-Trp(H)-Ala-OBn, HSQC

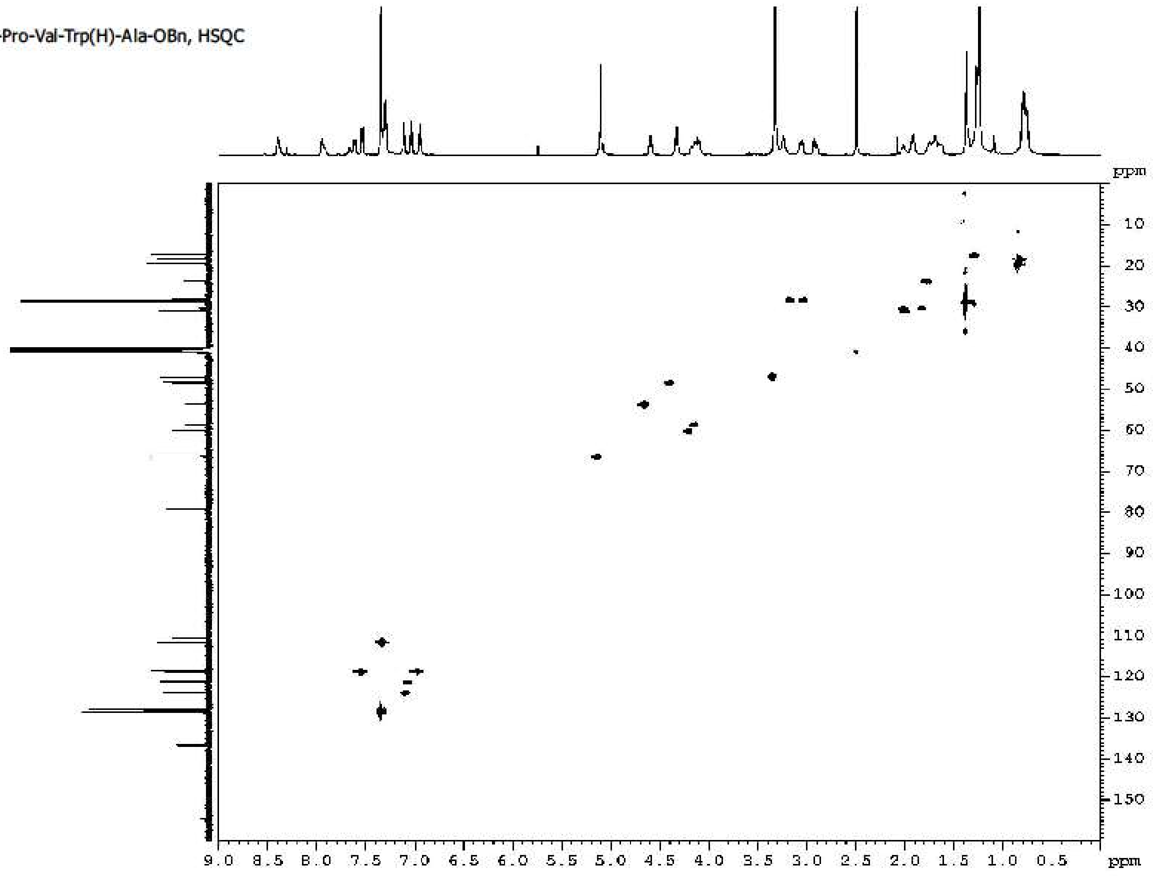

Boc-Pro-Val-Trp(H)-Ala-OBn, HMBC

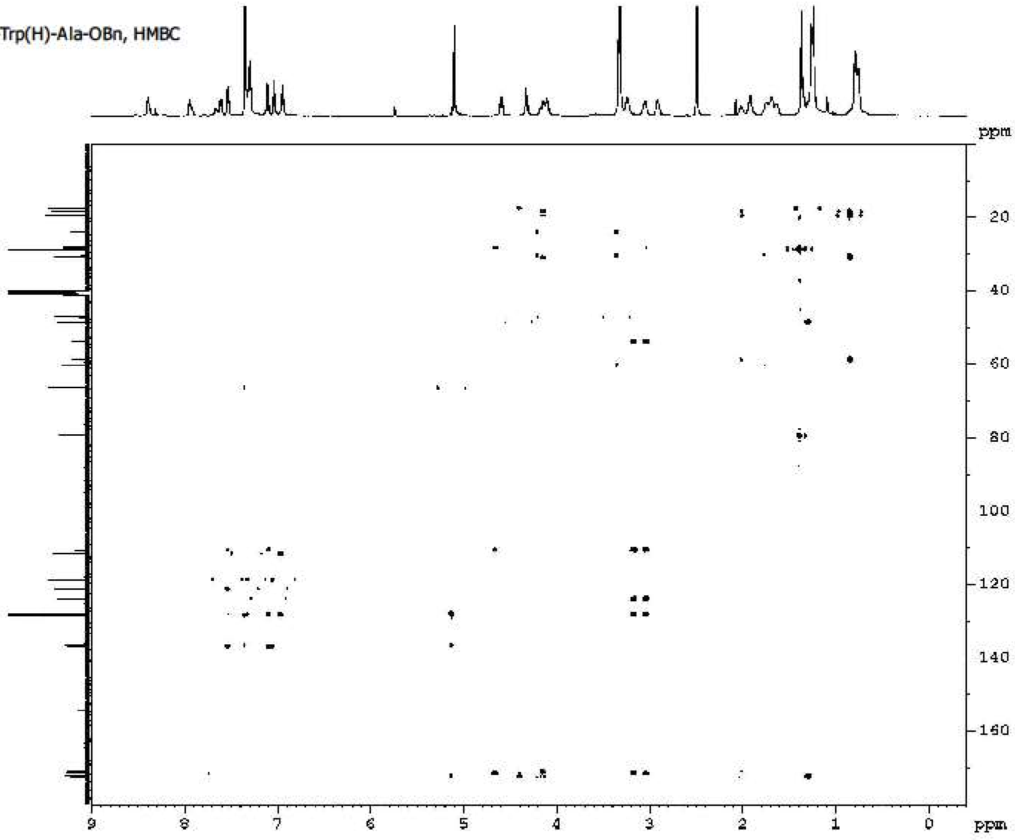

Boc-Gly-Val-Pro-Val-OBn (56)

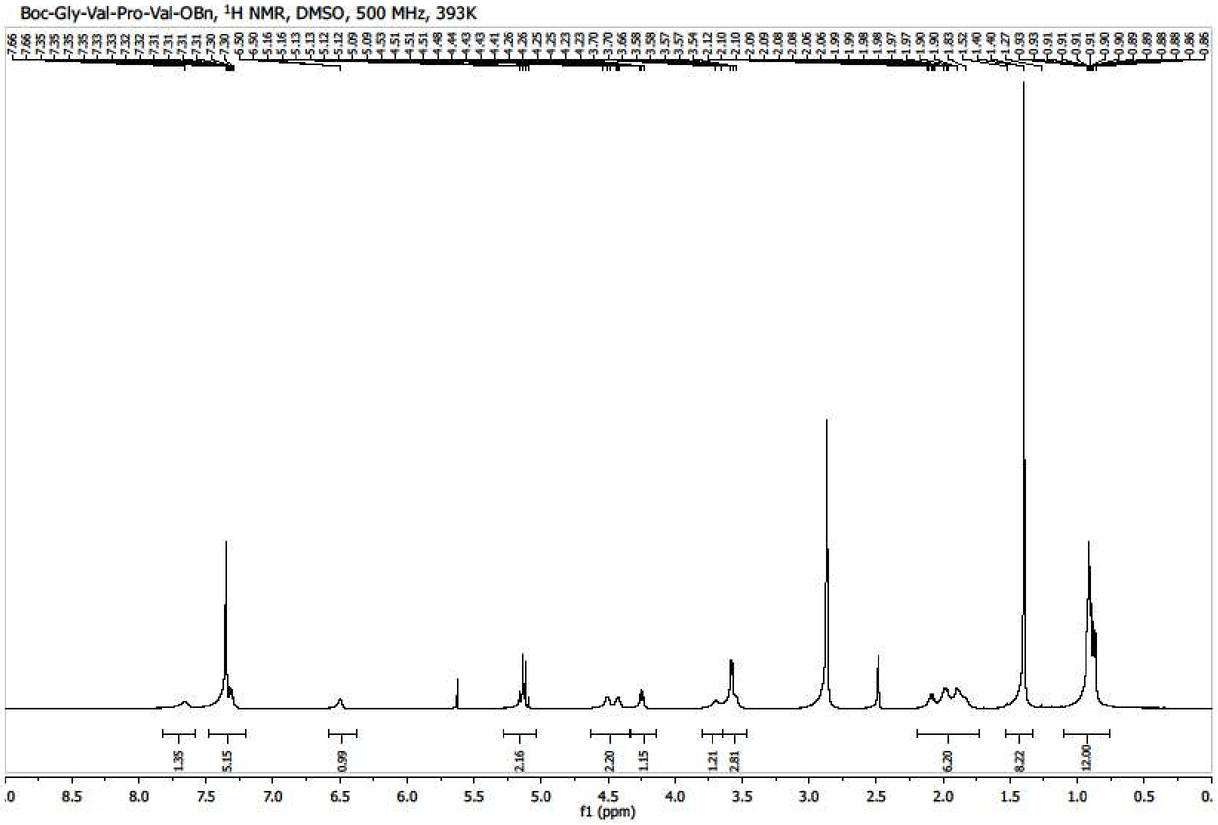

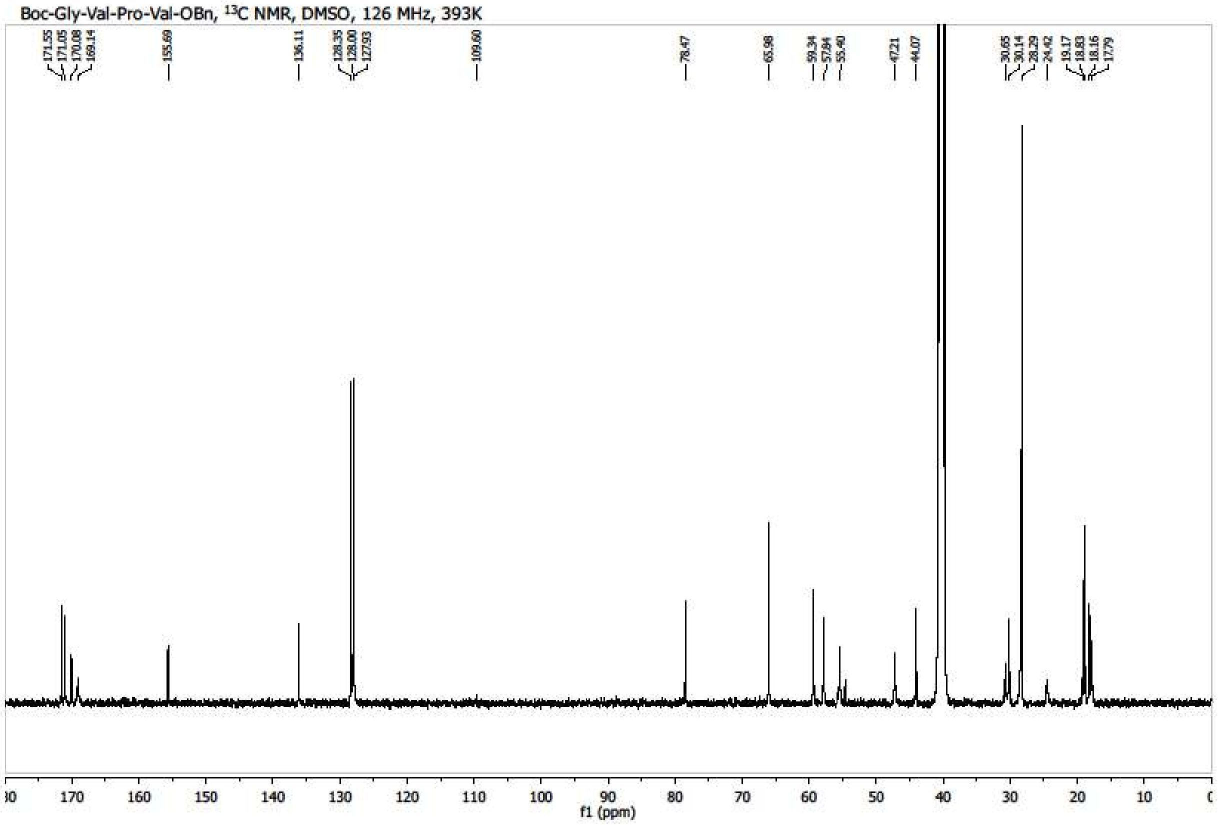

Boc-Gly-Val-Pro-Val-OBn, COSY

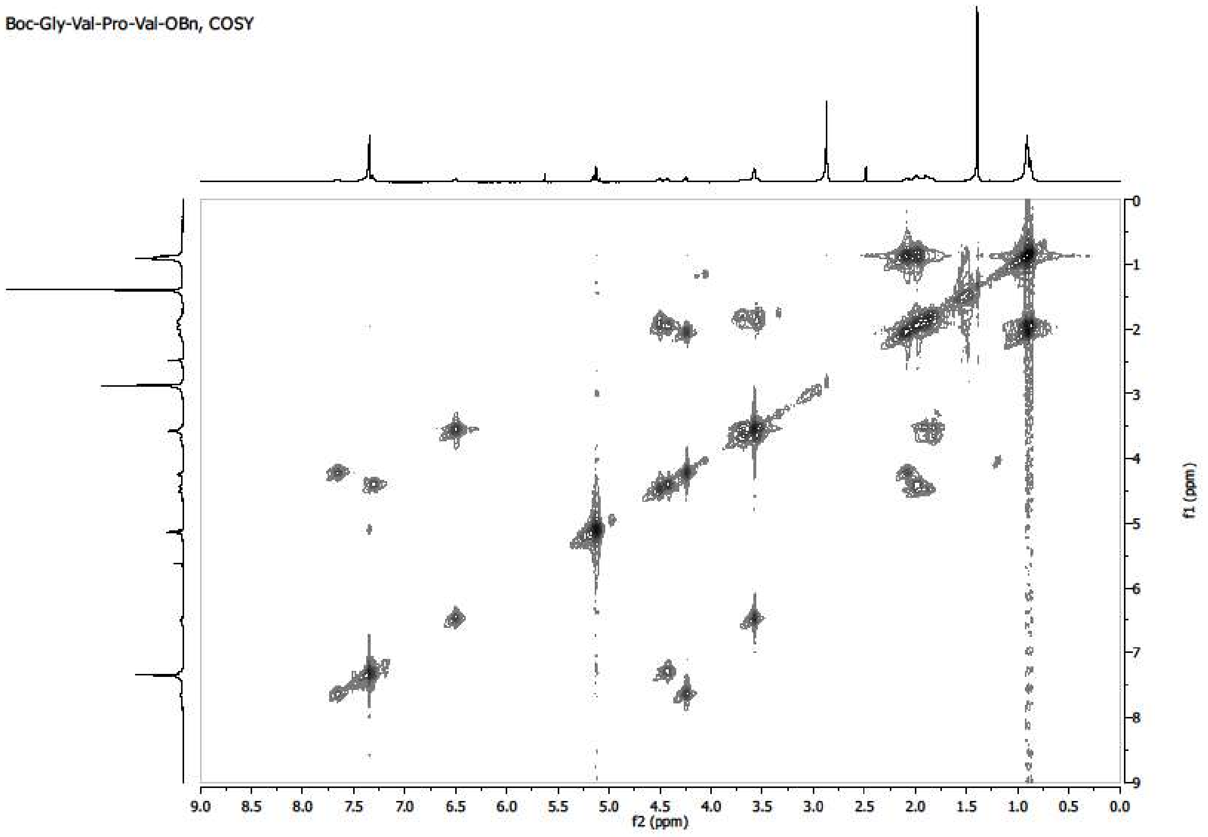

Boc-Gly-Val-Pro-Val-OBn, HSQC

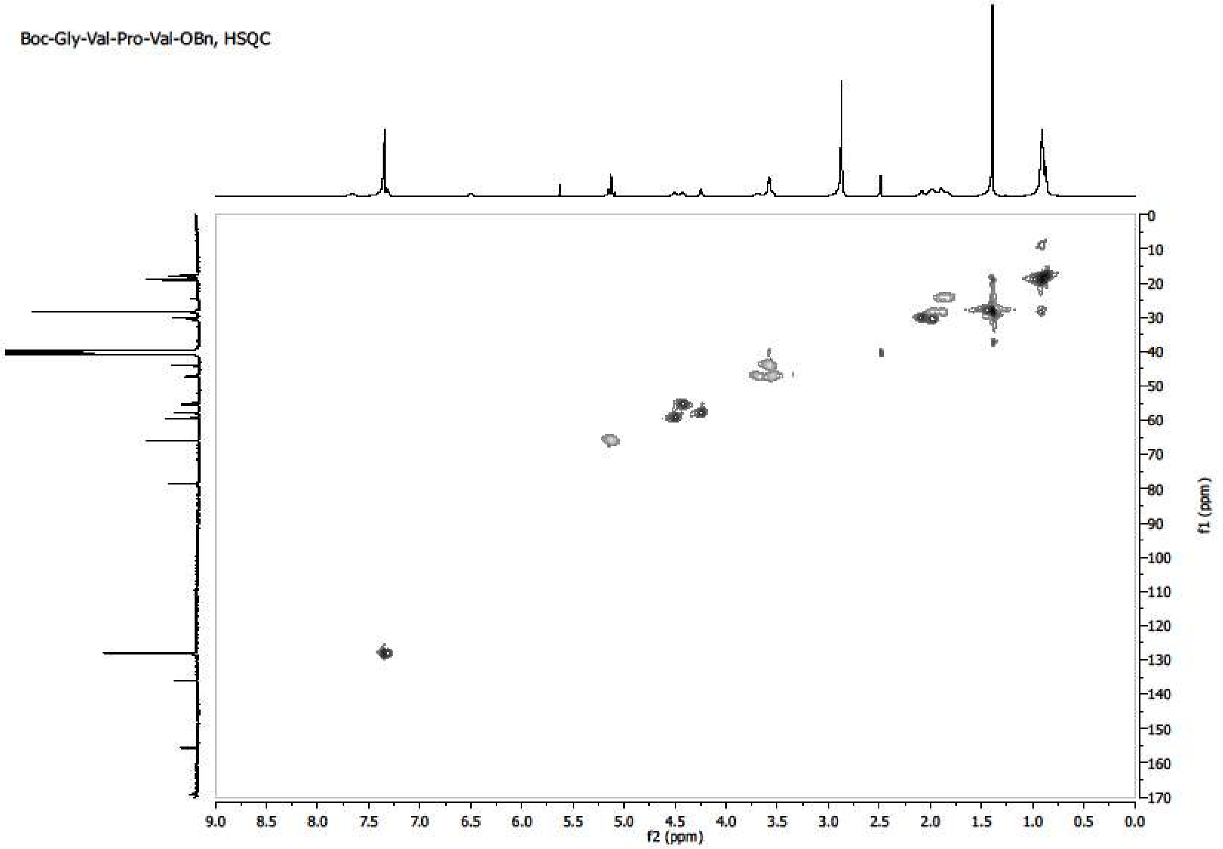

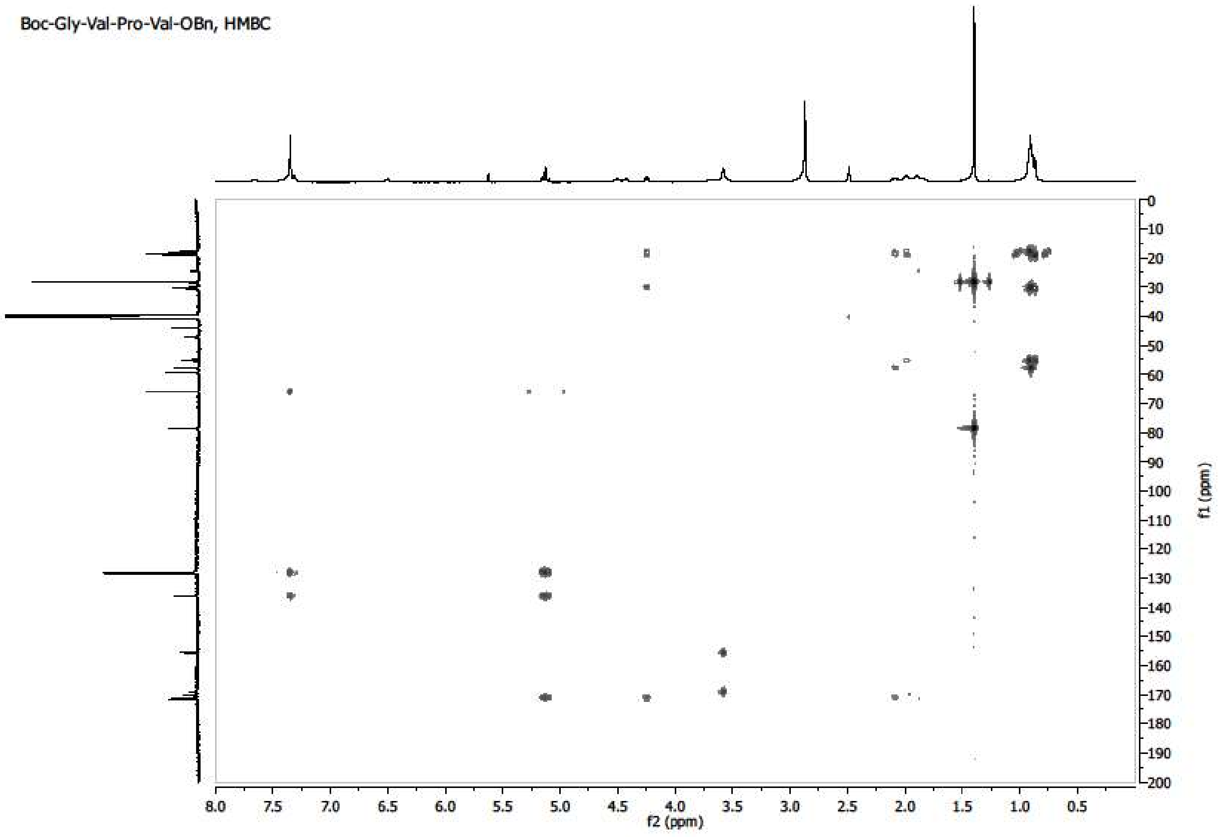

Boc-Trp(H)-Ala-Gly-Val-Pro-Val-OBn (5)

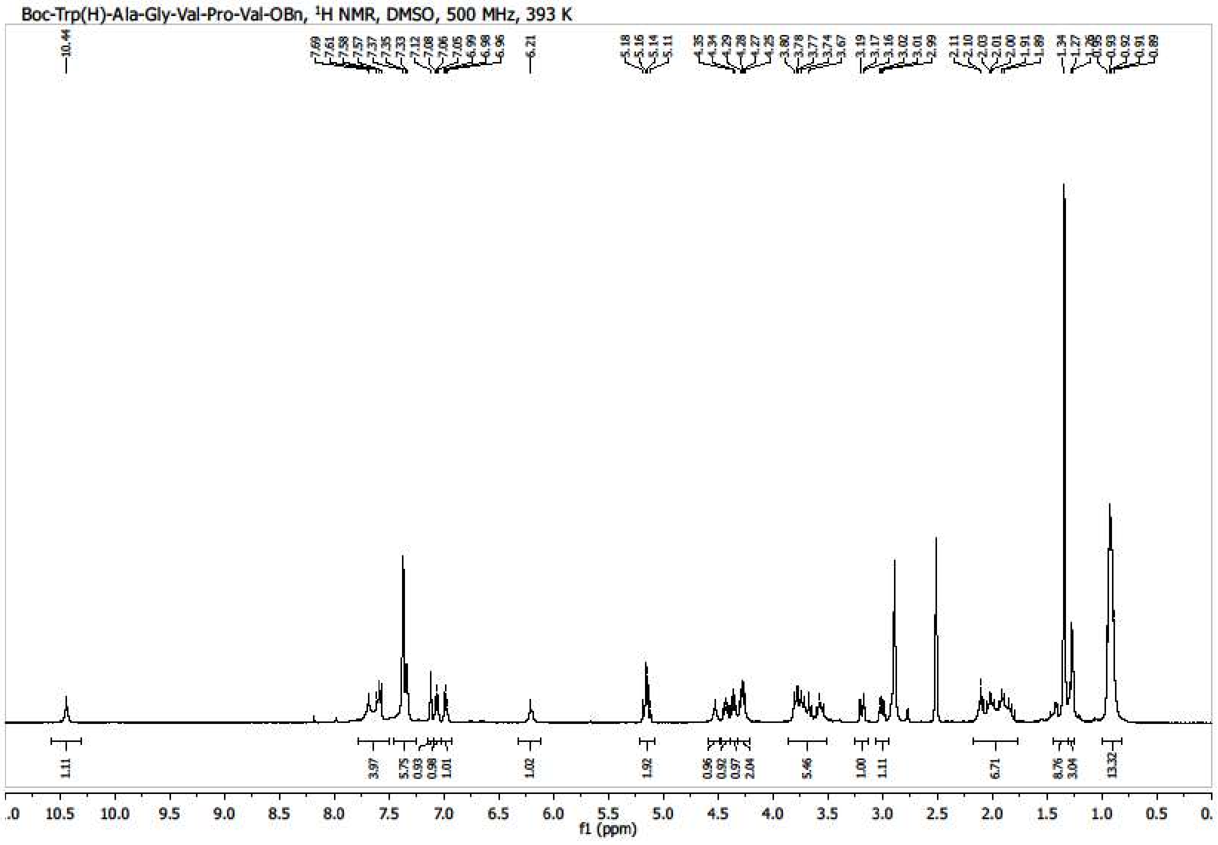

Boc-Trp(H)-Ala-Gly-Val-Pro-Val-OBn, ^{13}C NMR, DMSO, 126 MHz, 393 K

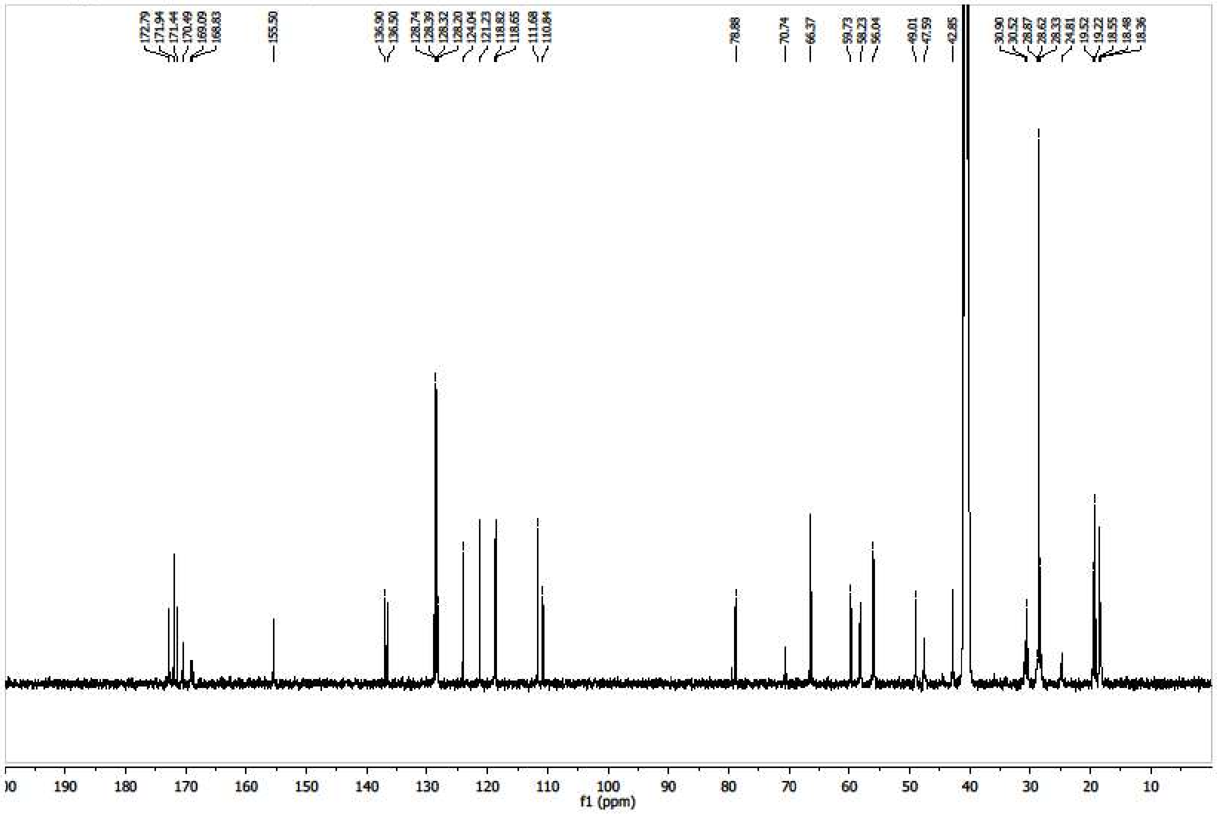

Boc-Trp(H)-Ala-Gly-Val-Pro-Val-OBn, COSY

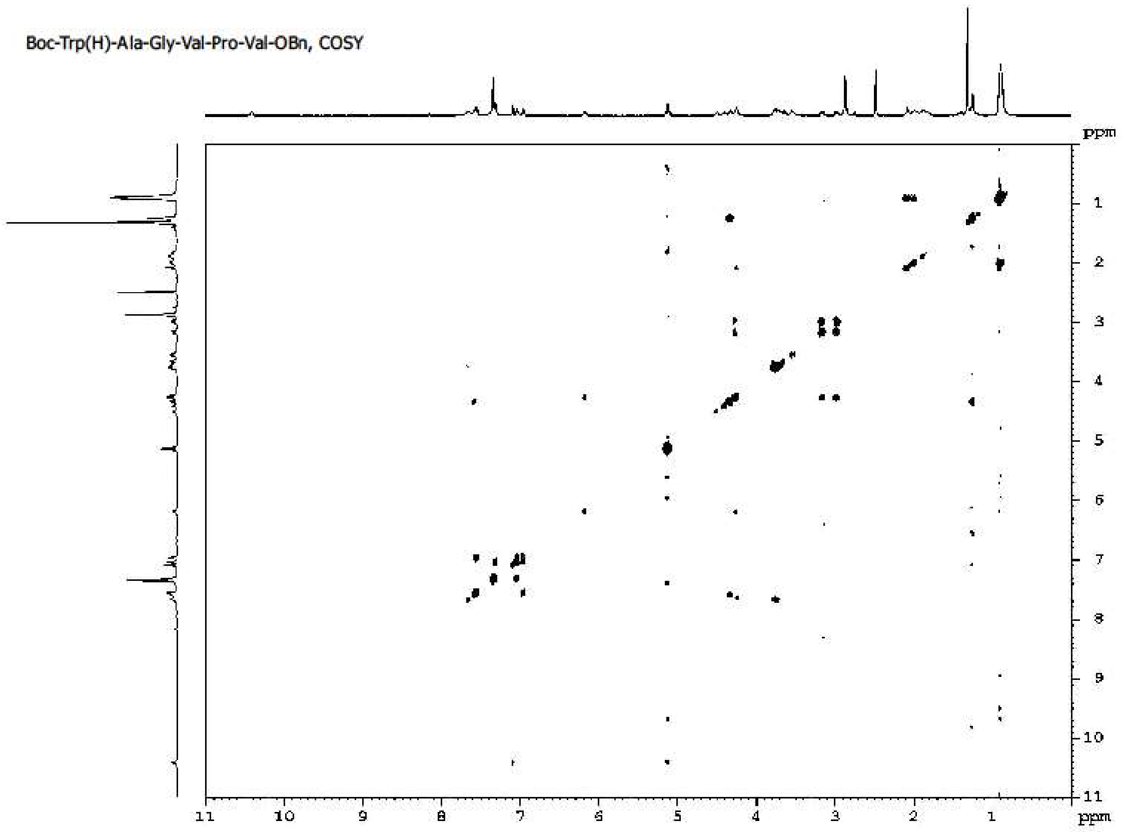

Boc-Trp(H)-Ala-Gly-Val-Pro-Val-OBn, HSQC

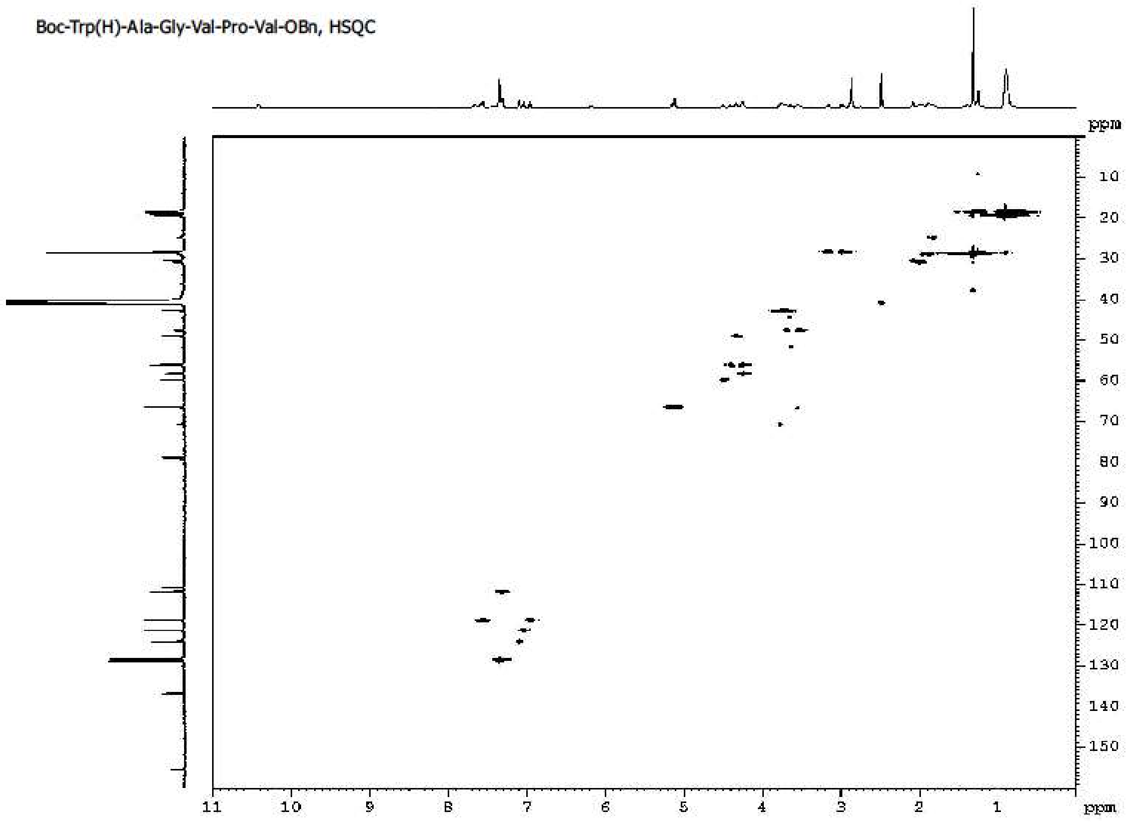

Boc-Trp(H)-Ala-Gly-Val-Pro-Val-OBn, HMBC

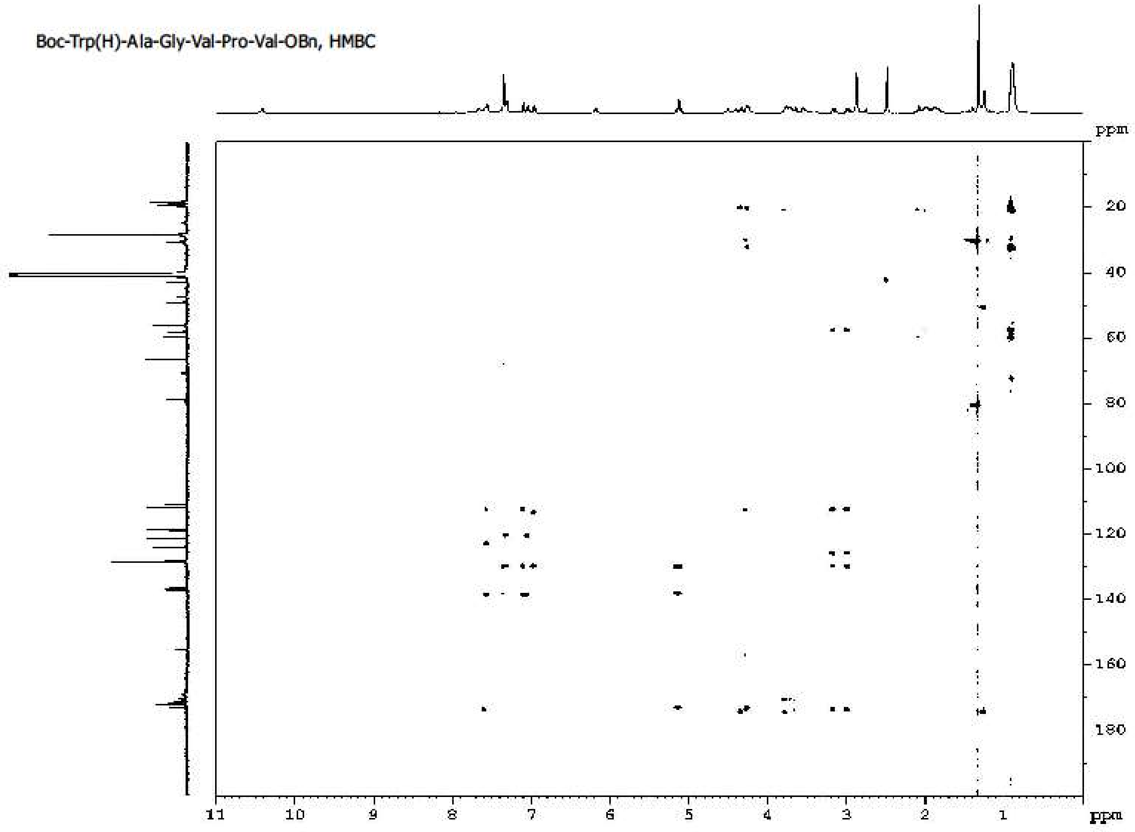

References

1. Vapourtec Ltd (2025) https://www.vapourtec.com/products/r-series-flow-chemistry-system-overview/

2. Vapourtec Ltd (2025) https://www.vapourtec.com/products/e-series-flow-chemistry-system-overview/

3. Cambridge Reactor Design Ltd (2025) https://www.polarbearplus.com/polar-bear-family/polar-bear-plus/

4. Mettler Toledo (2025) https://www.mt.com/us/en/home/phased_out_products/L1_AutochemProducts/ReactIR/FlowIR-continuous-flow-system.html

5. Newby JA, Blaylock DW, Witt PM, Pastre JC, Zacharova MK, Ley S V., Browne DL (2014) Design and application of a low-temperature continuous flow chemistry platform. Org Process Res Dev 18:1211–1220. https://doi.org/10.1021/op500213j

6. Lücke D, Dalton T, Ley S V., Wilson ZE (2016) Synthesis of Natural and Unnatural Cyclooligomeric Depsipeptides Enabled by Flow Chemistry. Chem Eur J 22:4206–4217. https://doi.org/10.1002/chem.201504457

7. Chandra J, Manne SR, Mondal S, Mandal B (2018) (E)-Ethyl-2-cyano-2-(((2,4,6-trichlorobenzoyl)oxy)imino)acetate: A Modified Yamaguchi Reagent for Enantioselective Esterification, Thioesterification, Amidation, and Peptide Synthesis. ACS Omega 3:6120–6133. https://doi.org/10.1021/acsomega.8b00732

8. Liu J, West KR, Bondy CR, Sanders JKM (2007) Dynamic combinatorial libraries of hydrazone-linked pseudo-peptides: Dependence of diversity on building block structure and chirality. Org Biomol Chem 5:778–786. https://doi.org/10.1039/b617217b

9. Ranganathan D, Vaish NK, Shah1 K (1994) Protein Backbone Modification by Novel Ca-C Side-Chain Scission. J Am Chem Soc 116:6545–6557

10. Tilak MA, Hoffmann JA (1977) Excess Azide Method of Peptide Synthesis. J Org Chem 42:2098–2100

11. Obeyesekere NU, La Croix J, Budde RJA, Dyckes DF, McMurray JS (1994) Solid-phase synthesis of (tyrosyl-alanyl-glutamyl)n, by segment condensation. Int J Pept Protein Res 43:118–126. https://doi.org/10.1111/j.1399-3011.1994.tb00512.x

12. Hänchen A, Rausch S, Landmann B, Toti L, Nusser A, Süssmuth RD (2013) Alanine Scan of the Peptide Antibiotic Feglymycin: Assessment of Amino Acid Side Chains Contributing to Antimicrobial Activity. ChemBioChem 14:625–632. https://doi.org/10.1002/cbic.201300032

13. Wu FC, Da CS, Du ZX, Guo QP, Li WP, Yi L, Jia YN, Ma X (2009) N-primary-amine-terminal β-turn tetrapeptides as organocatalysts for highly enantioselective aldol reaction. J Org Chem 74:4812–4818. https://doi.org/10.1021/jo9005766

14. Maji SK, Haldar S (2012) Effect of peptide architecture on the self-assembly properties of tripeptide based anionic surfactants issued from two different peptide sequences:

Ala-Ala-Val and Ala-Pro-Val in aqueous media (pH 7.4). Colloids Surf A Physicochem Eng Asp 414:422–432. https://doi.org/10.1016/j.colsurfa.2012.07.017
